# Boosting homogeneous chemoselective hydrogenation of olefins mediated by a bis(silylenyl)terphenyl-nickel(0) pre-catalyst†

**DOI:** 10.1039/d0sc06471h

**Published:** 2021-01-08

**Authors:** Marcel-Philip Lücke, Shenglai Yao, Matthias Driess

**Affiliations:** Department of Chemistry: Metalorganics and Inorganic Materials Technische Universität Berlin Strasse des 17. Juni 115, Sekr. C2 D-10623 Berlin Germany matthias.driess@tu-berlin.de

## Abstract

The isolable chelating bis(N-heterocyclic silylenyl)-substituted terphenyl ligand [Si^II^(Terp)Si^II^] as well as its bis(phosphine) analogue [P^III^(Terp)P^III^] have been synthesised and fully characterised. Their reaction with Ni(cod)_2_ (cod = cycloocta-1,5-diene) affords the corresponding 16 VE nickel(0) complexes with an intramolecular *η*^2^-arene coordination of Ni, [E(Terp)E]Ni(*η*^2^-arene) (E = P^III^, Si^II^; arene = phenylene spacer). Due to a strong cooperativity of the Si and Ni sites in H_2_ activation and H atom transfer, [Si^II^(Terp)Si^II^]Ni(*η*^2^-arene) mediates very effectively and chemoselectively the homogeneously catalysed hydrogenation of olefins bearing functional groups at 1 bar H_2_ pressure and room temperature; in contrast, the bis(phosphine) analogous complex shows only poor activity. Catalytic and stoichiometric experiments revealed the important role of the η^2^-coordination of the Ni(0) site by the intramolecular phenylene with respect to the hydrogenation activity of [Si^II^(Terp)Si^II^]Ni(*η*^2^-arene). The mechanism has been established by kinetic measurements, including kinetic isotope effect (KIE) and Hammet-plot correlation. With this system, the currently highest performance of a homogeneous nickel-based hydrogenation catalyst of olefins (TON = 9800, TOF = 6800 h^−1^) could be realised.

## Abstract 2

The isolable chelating bis(N-heterocyclic silylenyl)-substituted terphenyl ligand [Si^II^(Terp)Si^II^] as well as its bis(phosphine) analogue [P^III^(Terp)P^III^] have been synthesised and fully characterised.

## Graphical abstract

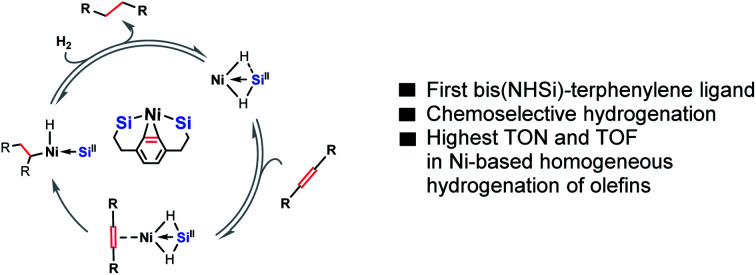

## Introduction

**Fig. 1:**
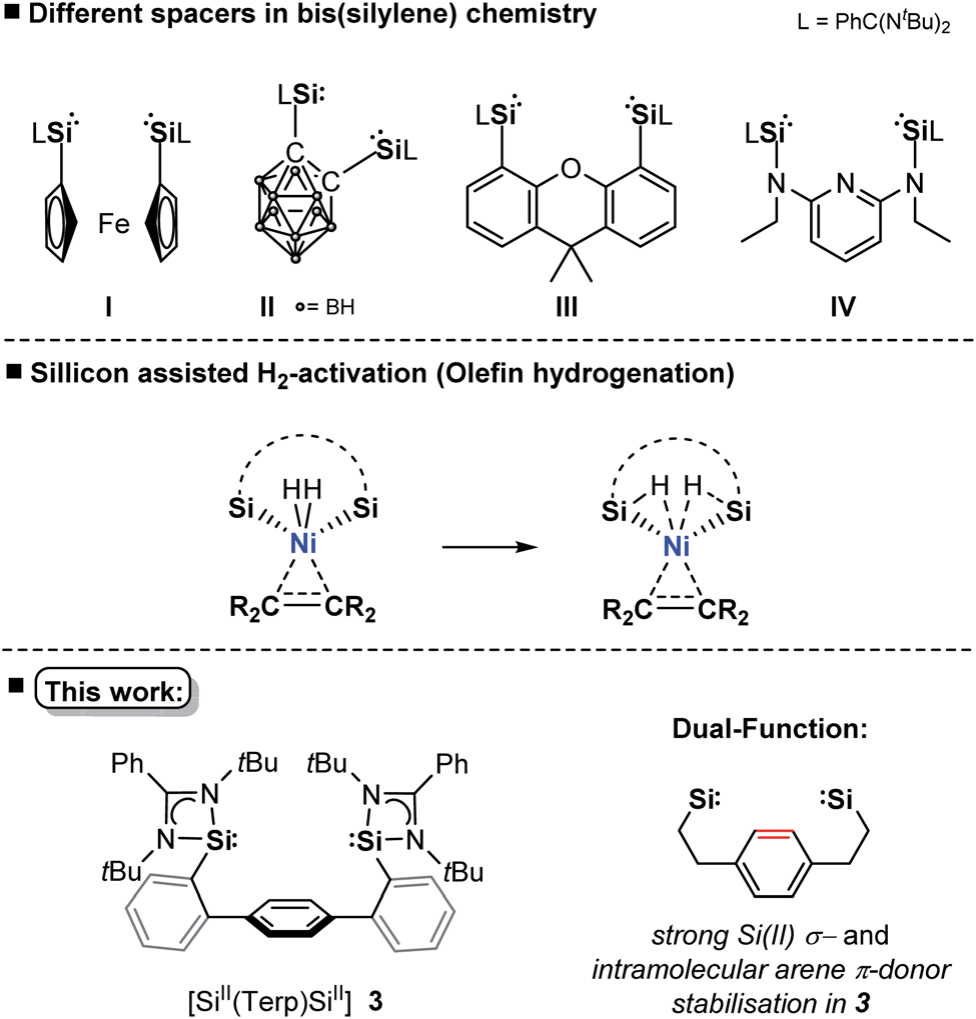
A phenylene-bridged bis(NHSi) ligand.

The transition-metal (TM) catalysed hydrogenation of unsaturated organic compounds is one of the most important reactions, where precious metals (Rh, Ir, Ru, Pd) proved to be particularly suitable.^1^ Current efforts are devoted to the utilisation of TM complexes of the first-row TMs as effective hydrogenation catalysts due to their higher natural abundance and reduced toxicity.^2^ However, the performance and selectivity of homogeneous TM catalysts greatly depend upon the development of suitable ligands which significantly influence the electronic and geometric properties of the pre-catalyst.^3^ Since 2001, isolable silylenes in particular N-heterocyclic silylenes (NHSis)^4*a*^ have been demonstrated to act as effective steering ligands in various TM-mediated catalytic transformations.^4*b*^ NHSis, the heavier analogues of N-heterocyclic carbenes (NHCs), exhibit a singlet electronic ground state with a strong σ-donor and π-acceptor character of the Si(ii) center towards TM sites. Experimental data indicate that the σ-donor strength of NHSi ligands greatly depends on the nature of the heterocyclic backbone.^4*b*,5^ DFT calculations revealed that NHSis can compete or even surpass the electronic properties of commonly used NHCs or phosphine ligands with respect to their σ-donor and π-acceptor strength and ligand-to-metal charge transfer ability.^5^ Various bis(NHSi) ligands were introduced (Fig. 1I–IV), whose TM complexes (Fe, Co, Ni, Mn, Ir, Rh) have shown high catalytic performance in hydrogenation of alkenes, ketones and other homogeneous catalytic transformations.^4*c*,6^ Compared with the application of ‘classical’ heterogeneous nickel catalysts in hydrogenation of olefins,^7^ homogeneous hydrogenations catalysed by nickel are far less explored.^8^ To facilitate the activation of dihydrogen, as one of the key steps in hydrogenation, it was shown that ligand–metal cooperativity is a powerful strategy to achieve heterolytic H_2_ splitting by introducing a Lewis acid (*e.g.* borane ligand) or Lewis base (*e.g.* amine ligand) coordinated to the active TM site.^8*e*,9^ Hanson *et al.* reported in 2012 a cationic nickel hydride complex bearing a polydentate bis(phosphine)-amine ligand which catalyses the hydrogenation of olefins under 4 bar H_2_ pressure at 80 °C.^10^ More recently, the Peters group introduced a bis(phosphine)-borane pincer-type ligand enabling the hydrogenation of olefins under ambient conditions (1 bar, rt).^8*e*,*f*^ After full conversion, the formation of a dinuclear nickel-hydride complex was observed, leading to a decreased hydrogenation activity. Alternatively, heterobimetallic Ni^0^ → M complexes (M = lanthanoid, group 13 metal) were also successfully employed to tune catalytic activity for the hydrogenation of olefins.^11^

In comparison to TM complexes with Ir, Ru, and Rh with turnover frequencies (TOFs) up to 15.000 h^−1^ and turnover numbers (TONs) of 4.55 × 10^6^, Ni-based homogeneous (pre)catalysts are still less active and selective.^12^ The current benchmark nickel pre-catalyst is [(dcpe)Ni(OAc)_2_] (dcpe = bis(dicyclohexylphosphanyl)ethane; OAc = acetate) introduced by Bouwman *et al.*, achieving TONs of up to 3000 in the hydrogenation of 1-octene within one hour at 50 bar H_2_ pressure.^13^

Based on aforementioned achievements in Ni-mediated hydrogenation, we wondered whether an intramolecular arene-Ni(0) coordination in bis(NHSi)Ni(0) complexes could boost the catalytic performance. Inspired by the work of Agapie and co-workers, a *para*-terphenyl-based bis(NHSi) ligand scaffold was targeted containing an intramolecular phenylene as an additional donor (Fig. 1, bottom).^14^ Herein, we report the synthesis of the new terphenyl-based chelating bis(NHSi) ligand [Si^II^(Terp)Si^II^] (**3**) and its 16 VE Ni(0)-complex [Si(Terp)Si]Ni(*η*^2^-arene) **5** which acts as an efficient and chemoselective catalyst for the hydrogenation of even functionalised olefins under ambient reaction conditions (1 bar, rt). In fact, with this system, the currently highest performance of a nickel-based hydrogenation of olefins (TON = 9800, TOF = 6800 h^−1^) could be realised.

## Results and discussion

### Synthesis and characterisation

**Scheme 1:**
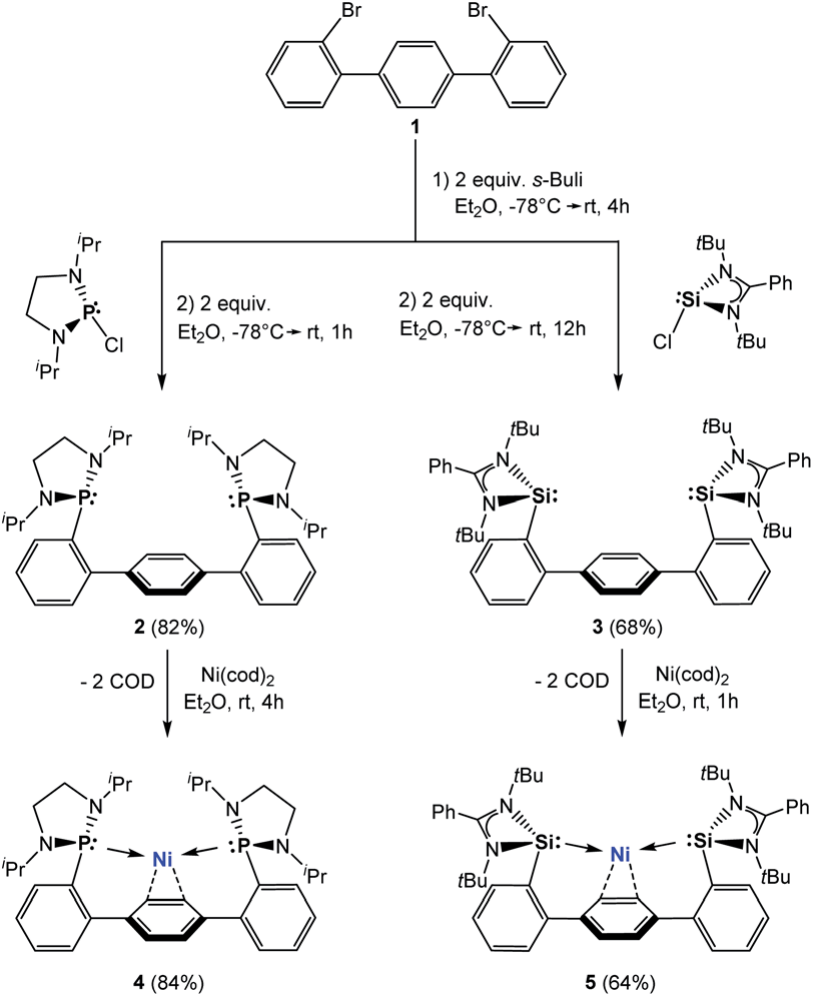
Synthesis of **2** and **3** and their nickel(0) complexes **4** and **5**, respectively.

**Fig. 2:**
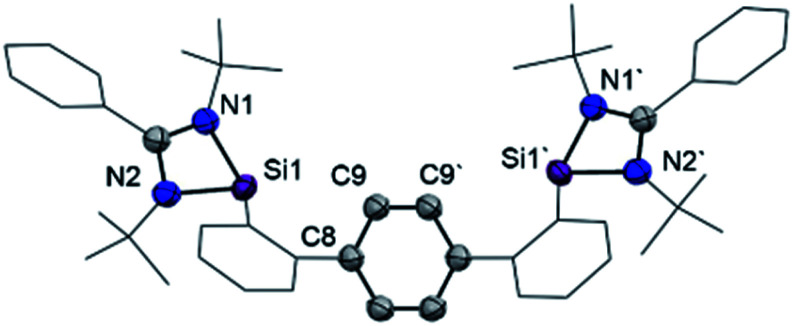
Molecular structure of bis(NHSi) **3** at 50% probability level. Hydrogen and solvent atoms are omitted for clarity. Selected distances [Å]: Si1–N1 1.887(3), Si1–N2 1.864(3), C9–C9′ 1.388(6), C8–C9 1.397(4).

Starting from 1,4-bis(2-bromophenyl)benzene (**1**, Scheme 1), both terphenyl-based chelating ligands [E(Terp)E] **2** (E = P^III^) and **3** (E = Si^II^) can be obtained upon lithiation with two molar equivalents of ^*sec*^BuLi followed by a salt-metathesis reaction with the [C_2_H_4_(N*i*Pr)_2_]PCl^15*a*^ and [PhC(N^*t*^Bu)_2_]SiCl,^15*b*^ respectively. Both products were isolated as pale yellow crystals in 82 (**2**) and 68% yields (**3**), respectively. Their molecular structures were unambiguously confirmed by NMR spectroscopic and X-ray diffraction analyses (Fig. 2; for **2**, see ESI†).

**Fig. 3:**
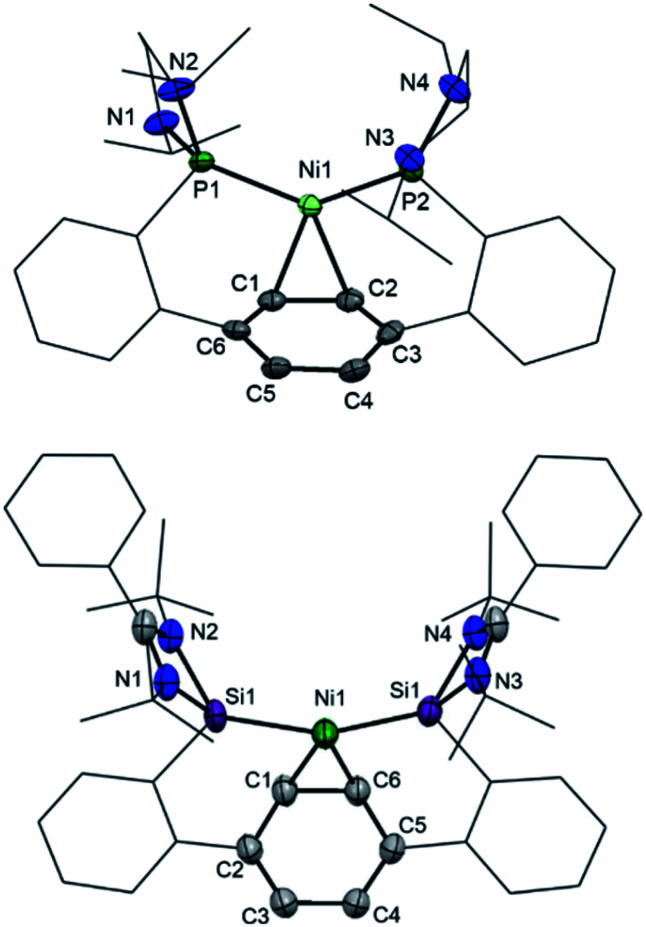
Molecular structures of **4** (top) and **5** (bottom) at 50% probability level. Hydrogen and solvent atoms are omitted for clarity. **4**: Selected bond lengths [Å]: P1–Ni1 2.1579(2), P2–Ni1 2.1461(4), Ni1–C1 1.9921(15), Ni1–C2 2.0041(15), C1–C6 1.423(2). Selected bond angle [°]: P1–Ni1–P1 131.004(18). **5**: Selected bond lengths [Å]: Si1–Ni1 2.20(4), Si2–Ni1 2.224(3), Ni1–C1 2.053(18), Ni1–C6 1.996(18), C1–C6 1.442(12), C1–C2 1.46(2), C2–C3 1.313(18). Selected bond angle [°]: Si1–Ni1–Si2 147.67(11).

Bis(phosphine) ligand **2** shows a singlet resonance signal in the ^31^P NMR spectrum at *δ* = 92.6 ppm, which is upfield-shifted compared to a carborane-based bis(N-heterocyclic phosphine) with *δ* = 114.3 ppm.^16^ The ^29^Si NMR spectrum of **3** shows a singlet at *δ* = 16.8 ppm similar to bis(NHSis) with carborane (*δ* = 18.9 ppm)- and xanthene (*δ* = 17.3 ppm) backbones.^6*a*,17^ Treatment of **2** and **3** with Ni(cod)_2_ (cod = cycloocta-1,5-diene) in Et_2_O at room temperature leads to the new 16 VE [E(Terp)E]Ni(*η*^2^-arene) complexes **4** (E = P^III^, 84%) and **5** (E = Si^II^, 64%), respectively, which were isolated as deep red crystals. Single-crystal X-ray diffraction analyses of **4** and **5** exhibit a trigonal-planar coordination geometry around the Ni^0^ center (Fig. 3). In both structures, the central phenylene ring is bound *η*^2^ to the Ni center (Ni1–C1: **4**: 1.992 Å, **5**: 2.053 Å), providing additional intramolecular stabilisation of the Ni^0^E_2_ (E = P^III^, Si^II^) subunit. The Si–Ni distances in **5** (2.20(4) and 2.224(3) Å) is similar to previously reported bis(NHSi)Ni^0^ complexes (Si–Ni: 2.15–2.23 Å).^6*a*,18^ Due to an enhanced σ-donor strength of Si^II^*vs.* P^III^, the C1–C6 bond is with 1.442(12) Å slightly elongated compared to bis(phosphine) nickel(0) complex **4** (d(C1–C2) = 1.423(2) Å).^4^

The ^29^Si NMR spectrum of **5** shows a singlet at *δ* = 52.0 ppm, which is strongly downfield-shifted with respect to the “free” bis(NHSi) ligand **3** (Δ^29^Si: 35.2 ppm) but upfield-shifted when compared to the xanthene based bis(NHSi)Ni(0) complex [**III**-Ni(*η*^2^-1,3-cod)] with *δ* = 61.4 ppm.^6*a*^ Additionally, **5** represents the first bis(NHSi) TM complex bearing a metal center exclusively stabilised by intramolecular donor ligands (Si^II^, bridging phenylene). The ^31^P chemical shift of **4** (*δ* = 103.6 ppm) is only slightly downfield-shifted compared to **2** (Δ^31^P: 11.7 ppm). Both **4** and **5** show line broadening in the ^1^H NMR spectra at room temperature (see ESI†). No reaction of **5** with an excess of PMe_3_ and even acetonitrile (MeCN) was observed based on multinuclear NMR analysis at room temperature. However, exposing the complexes **4** and **5** to CO furnishes a new diamagnetic Ni^0^-carbonyl species [E(Terp)E]Ni(CO)_2_ (E = P^III^, **4-CO**, Si^II^, **5-CO**) indicated by a fast color change from deep red to pale yellow. The IR stretching vibration frequencies of **5-CO** appear at *ν*_CO_ = 1970 and 1881 cm^−1^, which are bathochromically shifted compared to [**II**-Ni(CO)_2_] (*ν*_CO_ = 1982; 1934 cm^−1^),^16^ [Ni{(*t*BuNCH)_2_Si}_2_(CO)_2_] (*ν*_CO_ = 2000; 1941 cm^−1^),^19*a*^ [Ni(^*i*^Pr_3_P)_2_(CO)_2_] (*ν*_CO_ = 1987; 1926 cm^−1^)^19*b*^ and [P^III^(Terp)P^III^]Ni(CO)_2_ (**4-CO**, *ν*_CO_ = 1997; 1939 cm^−1^), respectively. The latter implies that the σ-donor strength of **3** is larger than that of **2**, and even exceeds that of **I** and **II** as chelating ligands, respectively.

### Catalyst screening and substrate scope

Choosing norbornene (Nbe, **9f**) as a well-known reference substrate for the hydrogenation of olefins, we compared the catalytic hydrogenation activity of the bis(phosphine)- and bis(silylene) stabilised Ni complexes **4** and **5**, respectively. The hydrogenation reactions were conducted in a sealed Young-NMR tube under 1 bar H_2_ pressure in C_6_D_6_ at ambient temperature. Using **5** as a pre-catalyst (Table 1, entry 1), a full conversion within 14 h under ambient conditions (C_6_D_6_,1 bar H_2_) to norbornane (**10f**) is achieved. After full conversion, no color change occurs, indicating the regeneration of the initial catalyst within the catalytic cycle. When **4** is used as a pre-catalyst (Table 1, entry 4), only 12% of norbornane (**10f**) is produced under the same conditions and 120 h are needed for completion. No hydrogenation occurs in the absence of **5** or using only the bis(NHSi) ligand **3** (6 mol%), which rules out any background activity (Table 1, entry 2). Employing **3** and Ni(cod)_2_ in a molar ratio of 1 : 1, full conversion is also achieved within 14 h (Table 1, entry 3). Quantitative hydrogenation occurs also in the presence of an excess of Hg in accordance with a homogeneous Ni catalyst (Table 1, entry 5). Prior exposure of **5** to air or CO results in complete loss of its hydrogenation activity (Table 1, entry 6). The substrate scope is further expanded to a variety of different substituted olefins (**9a–v**, 22 examples; Table 2).

**Table 1:** Catalyst screening for the hydrogenation of norbornene^a^

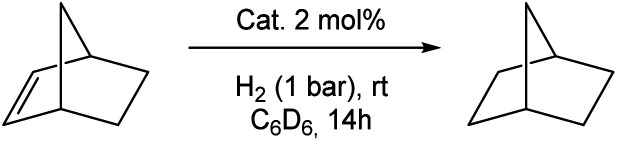		
Entry	Catalyst	Conv^a^
1	[Si^II^(Terp)Si^II^]Ni(*η*^2^-arene) **5**	>99%
2	[Si^II^(Terp)Si^II^](*η*^2^-arene) **3** (6 mol%)	0%
3	Ni(cod)_2_ + [Si^II^(Terp)Si^II^] **3** (1 : 1)	>99%
4	[P^III^(Terp) P^III^]Ni(*η*^2^-arene) **4**	12%
5	[Si^II^(Terp)Si^II^]Ni(*η*^2^-arene) **5** + Hg^b^	>99%
6	[Si^II^(Terp)Si^II^]Ni(*η*^2^-arene) **5** + air or CO	0%

a Reaction conditions: norbornene (0.054 mmol), ferrocene (0.022 mmol, int. standard) and 2 mol% Ni catalyst in 0.45 mL C_6_D_6_ under 1 bar H_2_ at RT for 14 h. Conversion was determined by ^1^H NMR.

b Hg to catalyst ratio 250 : 1.

**Table 2:** Substrates scope of **5**- catalysed olefin hydrogenation^a^^b^^c^

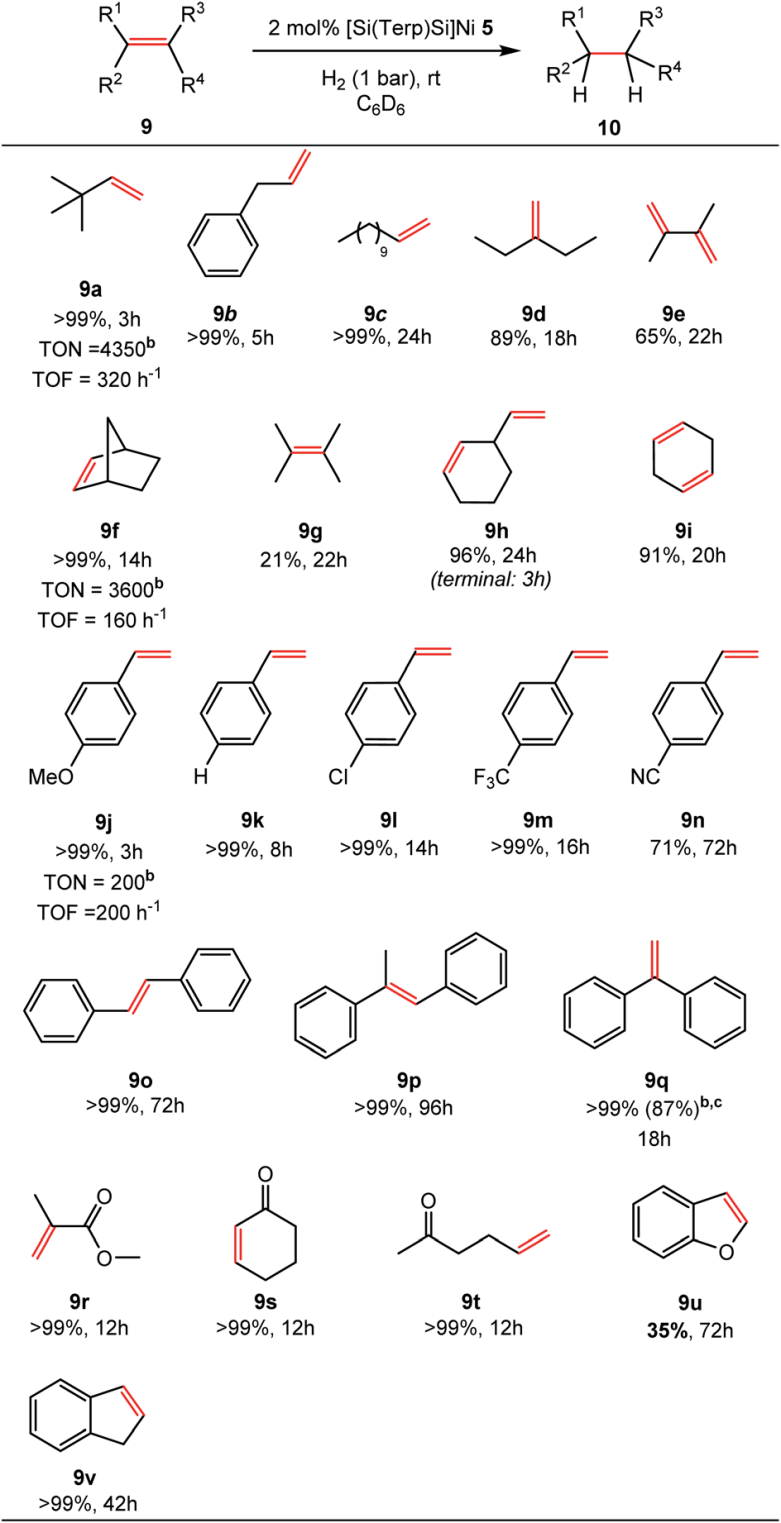

a Reaction conditions: olefin (0.054 mmol), ferrocene (0.022 mmol, int. standard) and 2 mol% catalyst **5** in 0.45 mL C_6_D_6_ under 1 bar H_2_ at RT without stirring. Conversion was determined by ^1^H NMR.

b Reaction conducted in a Schlenk tube containing a stir bar (1400 min^−1^).

c Isolated yields.

Quantitative hydrogenation was achieved for most substrates (16 examples, >99%). A fast hydrogenation was observed for a number of unactivated, alkyl-substituted alkenes (**9a–i**). Isomerisation of 2,3-dimethyl-1,3-butadiene (**9e**) to the bulky tetramethylethylene (**9g**) takes place after monohydrogenation yielding 65% of the corresponding alkane (**10e**) after 22 h, while isomerisation to 1-ethylcyclohex-1-ene (4%) is observed, affording 96% yields of ethylcyclohexane (**10h**) after 24 h. In the course of 1,4-cyclohexadiene (**9i**) hydrogenation, the formation of benzene is observed (9%, *via* dehydrogenation) leading to 91% yields of cyclohexane (**10i**). For the unactivated tetramethylethylene, 21% conversion was achieved within 22 h.

Olefins containing aromatic substituents such as styrene (**9k**), stilbene (**9o**, **p**) and 1,1-diphenylethylene (**9q**) are quantitatively hydrogenated. Functional groups such as methoxy, chlorine, and trifluoromethyl are tolerated (**9j**, **9l**, **9m**). However, 4-cyanostyrene (**9n**) is only partly converted (71%, *t* = 72 h) and decomposition of **5** occurs. Hydrogenation of unsaturated carbonyl compounds (**9r–t**) containing isolated (**9r** and **9t**) and internal C=C bonds (**9s**) are fully converted with excellent chemoselectivity to the respective ketones and carbonate within 12 h. The hydrogenation of an α,β-unsaturated ester **9r** could also achieved. Bicyclic 1*H*-indene (**9v**) and benzofuran (**9u**) have additionally been tested. Benzofuran was hydrogenated in 35% yields after 72 h, in contrast to 1*H*-indene (**9v**), which was quantitatively hydrogenated after 42 h.

In order to determine the catalytic activity of **5** with respect to turnover number (TON) and turnover frequency (TOF, h^−1^), hydrogenation reactions were performed in a Schlenk-tube containing a metal-free teflon-coated stir bar (1400 min^−1^). Using 0.026 mol% of **5** and 350 mg of norbornene (**9f**) results in a quantitative hydrogenation (TON = 3700) reaching a TOF value of 160 h^−1^. A similar TOF value is obtained using 0.1 mol% (TOF = 170 h^−1^). An even higher value of TOF = 320 h^−1^ can be realised using 3,3-dimethylbut-1-ene (**9a**) in C_6_D_6_ achieving a quantitative hydrogenation in the presence of 0.023 mol% (TON = 4350). To determine the maximum TON, the experiments were repeated employing 1.2 mL **9a** (9.3 mmol) in 1.0 mL d_8_-THF resulting in 98% conversion after 48 h (TON_MAX_ = 9800). The TOF_MAX_ is determined under the same conditions using only 0.2 mL THF as solvent which results in 68% conversion after 1 h (TOF_MAX_ = 6800 h^−1^). These results are excellent among Ni(0)-based homogeneous catalyst with the highest TON and TOF values previously reported.^6*a*,17^ In the presence of 0.5 mol% of catalyst **5**, 1,1-diphenylethylene (**9m**) is quantitatively hydrogenated within 24 h on a larger scale (0.27 mmol), affording pure **10m** in 87% isolated yields after filtration.

### Dihydrogen activation by pre-catalyst **5**

**Scheme 2:**
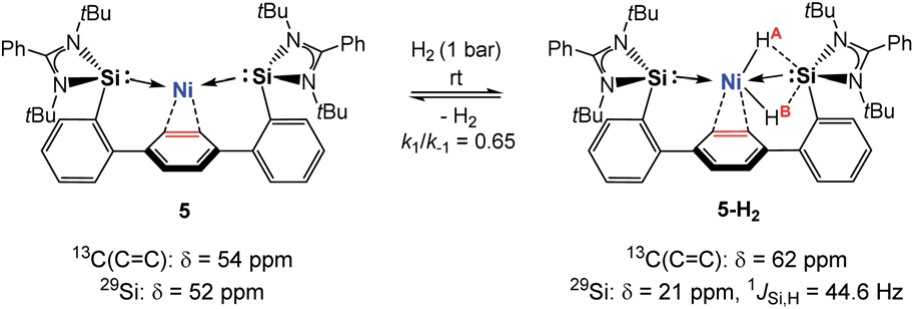
Proposed structure of the dihydrido Ni(ii) complex **5-H2**.

Hydrido TM complexes are important intermediates in catalytic hydrogenation reactions. They can be formed through the oxidative addition of H_2_ to a TM atom *via* formation of a side-on M-*η*^2^(H_2_) dihydrogen complex. A cornerstone in this context is the first isolation of such a dihydrogen complex in 1984 by Kubas *et al.*^20^ After the pioneering studies of Ni-*η*^2^(H_2_) complexes by Caulton and co-workers in 2010, a number of stable dihydrogen-TM complexes were isolated by the groups of Tsay, Peters, Heinekey and Lu.^21^ In 2017 our group reported the isolation of the first silylene-assisted dihydrido Ni complex showing an additional Ni–H → Si^II^ bonding interaction as confirmed by *in situ* NMR spectroscopy and a single-crystal X-ray diffraction analysis.^6*a*^ Further insights into this new Si–Ni cooperative activation mode were provided by DFT calculations suggesting that the dihydrogen activation is achieved by interaction of the Ni 3d_*xy*_ orbital and the σ(Ni–Si) with the σ*(H–H) orbital. Similarly, the Peters group found that (^Ph^DPB^*i*Pr^)Ni [^Ph^DPB^*i*Pr^ = PhB(*o-i*Pr_2_PC_6_H_4_)_2_)] can activate H_2_ affording a bridging hydrido-borohydrido Ni complex, where the B and Ni atoms cooperatively cleave H_2_.^11^ The reaction of **5** with dihydrogen under 1 bar in *d*_8_-THF at 298 K furnishes the new diamagnetic nickel complex **5-H2** as indicated by a singlet resonance signal in the ^1^H NMR spectrum at *δ* = – 1.50 ppm with ^29^Si-satellites (^1^*J*_Si,H_ = 44.6 Hz) and a singlet at *δ* = +5.77 ppm for the central phenylene protons (Scheme 2). The observed Si–H bonding interaction is in line with the previously described bis(NHSi)dihydrido Ni complex showing an additional Ni–H → Si^II^ bonding interaction with ^1^*J*_Si,H_ = 44.2 Hz.^6*a*^ A mixture of the starting material **5** and **5-H2** exists in an equilibrium ratio of ∼2 : 1 (**5 : 5-H2**, *k*_1_/*k*_−1_ ≈ 0.65). This process is reversible, and removal of H_2_ by successive freeze–pump–thaw cycles regenerates 5 quantitatively. Several attempts to crystallise the dihydrido Ni(ii) complex failed leading to the isolation of **5** under 1 bar H_2_ pressure.

**Fig. 4:**
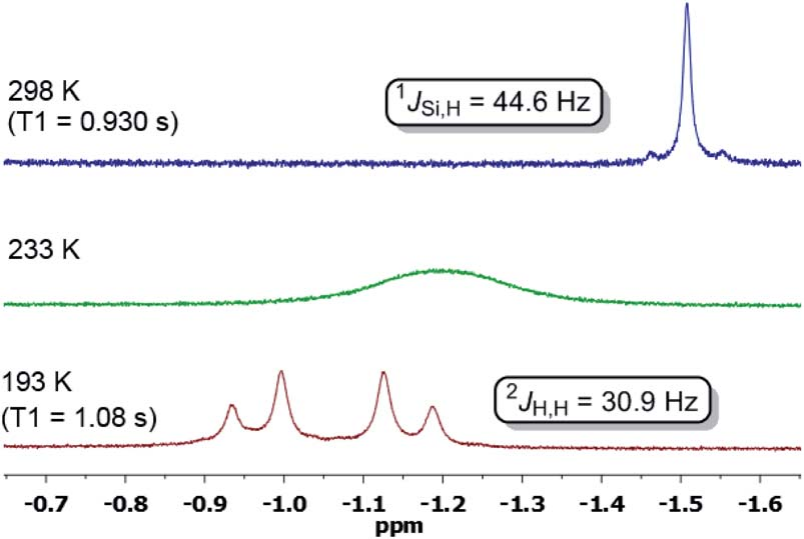
^1^H NMR spectra of the hydride region at variable temperature of a sample of **5** + H_2_ in a sealed Young NMR tube.

In contrast, dihydrogen activation was not obtained for the bis(phosphine)Ni(0) complex **4** under the same conditions. The assigned ^29^Si NMR resonance signal (*δ* = 21.0 ppm, by ^1^H,^29^Si-HMQC) is upfield-shifted by Δ*δ* = 31 ppm (**5**: *δ*(^29^Si) = 52 ppm) and splits into a doublet in the ^1^H-coupled ^29^Si NMR spectrum (^1^*J*_Si,H_ = 44.6 Hz) due to a significant bonding interaction between the Si and H atoms. Based on the ^29^Si shift, **5-H2** can be seen as a bis(NHSi)-supported dihydrido Ni(ii) complex which is additionally stabilised by the intramolecular phenylene ring as confirmed by a ^1^H,^13^C-HSQC NMR analysis of **5-H2** at 193 K. Variable temperature ^1^H NMR spectroscopy (VT-NMR, **5** + H_2_, 500 MHz) was performed in the range of 298 to 193 K revealing a coalescence temperature at *T*_c_ = 233 K showing one broad signal (*v*^1/2^ = 75 Hz, Fig. 4). At lower temperature (*T* < *T*_c_) a well-resolved spectrum is obtained, exhibiting a doublet of doublet pattern for two non-equivalent H atoms (H^A^,H^B^: ^2^*J*_HH_ = 30.9 Hz) as the inversion rate at nickel slows down (*k*_233_ = 275 s^−1^, Δ*v* = 99.5 Hz).^22^ The free activation energy Δ*G*193‡ for this process is calculated with Δ*G*193‡ = 10.9 kcal mol^−1^ comparable with reported *cis*-dihydrido complexes.^23^ The values of the spin-lattice proton relaxation time (*T*_1_, *d*_8_-THF, 500 MHz), measured at 298 K (*T*_1_ = 1080 ms) and 193 K (*T*_1_ = 930 ms, 500 MHz) are larger than typical values for TM-dihydrogen complexes (*T*_1_ < 35 ms).^24^ Addition of *D*_2_ at –80 °C to a sample of **5** + H_2_ (1 bar, rt) further confirmed the reversible H_2_ activation by HD-scrambling. The HD isotopomer **5-HD** shows a triplet resonance signal in the ^1^H NMR spectrum at *δ* = –0.92 ppm (^2^*J*_D,H_ = 4.66 Hz) due to ^2^*J* proton-deuterium (*I* = 1) coupling.^24^ For **5-HD**, only two singlet resonances are obtained in the ^1^H NMR spectra (*d*_8_-THF) at 193 K due to the absence of ^2^*J*_H,H_ coupling. The mono- and dihydrido signals coalesce into a broad signal at *T*_c_, indicating fast exchange between **5-HD**/**5-H2** and their structural similarity (see ESI†).

### Mechanistic investigation

Sub-stoichiometric reactions were performed to further study the catalytic hydrogenation reaction catalysed with **5** in *d*_8_-THF. Exposure of **5** to five molar equiv. of norbornene (Nbe, **9f**) led to the partial formation of the new diamagnetic Ni(0)-species **5-nbe**, suggesting a reversible nbe/phenylene exchange reaction observed in the ^1^H NMR spectra (see ESI†).^25^ Addition of H_2_ (1 bar) results in the partial formation of **5-H2**. However, the hydrogenation under these conditions with low substrate loading affords a very slow conversion, indicating that the **5-nbe** complex is the active catalytic species with an increasing concentration at higher substrate loadings. The ratio of **5-nbe** and **5-H2** is found to be 3 : 1 under these conditions suggesting **5-nbe** as catalytic active resting state. After full conversion, the hydride shift is found at *δ* = –1.49 ppm in line with the hydride-shift of **5-H2** with ^29^Si-satellites (^1^*J*_Si,H_ = 44.6 Hz). Upon addition and hydrogenation of an additional 20 equiv. of nbe (5 mol% Ni) the same results are obtained and no deactivated resting state formation occurs. Only a mixture of **5** and **5-H2** is obtained based on ^1^H NMR analysis. The same is true under lower catalytic loading of even 2 mol% of **5**. As one of the aims of this work, the nickel site is stabilised due to an intramolecular C=C coordination of the phenylene moiety in the terphenyl-scaffold after full conversion. This is further supported by a cycling experiment using alkene 3,3-dimethyl-1-butene (**9d**). After full conversion of the first cycle, another 50 equiv. alkene were added. Similarly, a full conversion is achieved within 3 hours under standard hydrogenation conditions (1 bar, rt).

To gain quantitative kinetic data on the alkene hydrogenation catalysed by **5**, kinetic studies were carried out using norbornene.^26^ Two solutions containing **5** (5.6 μM) and different amounts of norbornene (0.10; 0.20 mM) were exposed to H_2_ (1 bar) in Young NMR tubes. The progress of these reactions was then monitored by ^1^H NMR spectroscopy. The olefin consumption is found to be zero-order in olefin and a plot of *k*_obs_ against the concentration of the catalyst indicated that the reaction is first-order in **5**. Similarly, zero-order dependence on the substrate was previously observed for the hydrogenation of olefins in the presence of a cationic rhodium(i) or rhenium(iii) complex and imine hydrogenation assuming a pre-equilibrium leading to saturation behaviour.^27^ In line with a zero-order dependence in the substrate concentration, the hydrogenation of norbornene reveals a linear kinetic profile (conv. → *f*(*t*)) with no induction period in C_6_D_6_ using 0.1 mol% **5**. To determine the reaction order with respect to H_2_, three solutions of norbornene (0.14 M) and **5** (2.5 mM) were subjected to different H_2_ pressures (1.0, 2.0, 3.0 bar) inside a Schlenk tube containing a stir bar. The dependence on the H_2_-pressure is found to be first order.^28^

**Fig. 5:**
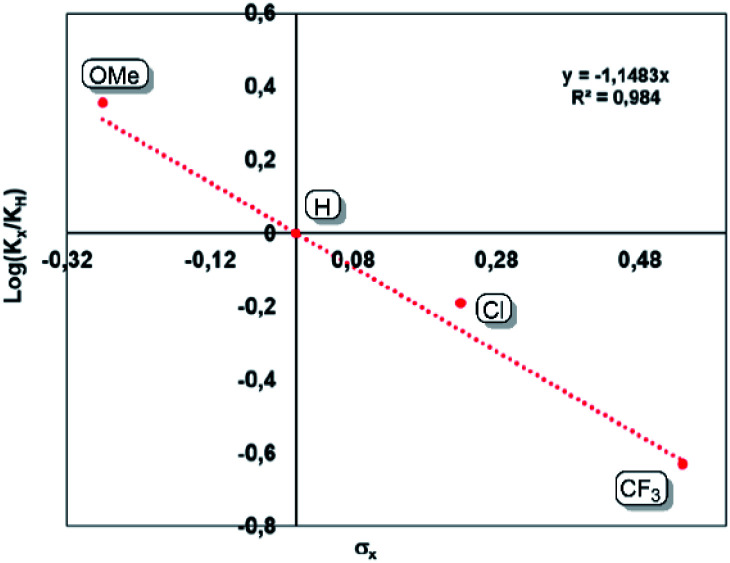
Generated Hammet-plot from the hydrogenation of *para* substituted styrene derivatives.

An inverse secondary kinetic isotope effect (SIKIE, *k*_H_/*k*_D_ = 0.83) is found for the hydrogenation of norbornene, indicating a late transition state, in which the H–H bond activation is not included in the rate-limiting step (RLS).^29^ Similarly, a KIE of 0.9 was reported for the Wilkinson's catalyst.^29*b*,*c*^ The SIKIE further supports a rate-limiting step in which hybridisation changes from sp^2^ → sp^3^ on carbon. To gain more information about the electronic nature of the rate limiting step, different *para*-substituted styrene derivatives (*p*-X-styrene, X = OMe, H, Cl, CF_3_) were hydrogenated inside a Schlenk tube containing a stir bar. The progress of these reactions was monitored by ^1^H NMR spectroscopy. A linear reaction profile is obtained using 0.5 mol% of bis(NHSi)Ni(0) catalyst **5** within initial 3 hours (Fig. 5). The kinetic data display a strong dependence between the reaction rate and the electronic nature of the *para*-substituent. For electron-withdrawing groups (EWG, X = Cl, CF_3_) lower reaction rates are obtained, while introducing an electron-donating group (EDG) *e.g.* a methoxy group reveals full conversion within one hour (TON = 200 h^−1^). The kinetic data are correlated with the standard Hammet *σ*_para_ values,^30^ resulting in a negative slope of *ρ* = −1.15 rationalised by the stabilisation of a partial positive charge which is built up at the benzylic carbon in the rate-limiting step (Fig. 5). This step includes the insertion of the olefin into the Ni–H bond by a sp^2^ → sp^3^ change of hybridisation on carbon yielding the Ni(ii)-alkyl-hydrido complex which is in line with a SKIE <1 (0.83). This step might also correspond to the turn-over-limiting step (TLS). In fact, using THF (*ε* = 7.58) as polar solvent increases the TOF to 334 h^−1^ on 4-methoxy styrene **10j** compared to benzene as a solvent (*ε* = 2.28, TOF = 200 h^−1^) using 0.5 mol% of **5**.

Similarly to the hydrogenation of olefins by the xanthene-supported bis(NHSi)-stabilised nickel complex,^6*a*^ an olefin pathway mechanism is proposed, starting with the reversible coordination of the olefin (*e.g.* norbornene) *via* an intramolecular phenylene/external substrate exchange reaction, yielding the catalytically active species **5-nbe**. Reaction with dihydrogen yields a Si^II^-assisted dihydrido–Ni complex (16 VE). Then, the olefin inserts into the Ni–H bond which is found to be rate-limiting based on KIE measurements and Hammet-plot analysis. Hydride-transfer results in the reductive elimination under liberation of the alkane, regenerating **5**. In line with the experimental findings, a mixture of **5** and **5-H2** is obtained and no deactivated resting-state formation occurs.

## Conclusions

In summary, the first chelating terphenyl-based bis(NHSi) ligand [Si^II^(Terp)Si^II^] **3** as well as its phosphine analogue [P^III^(Terp)P^III^] **2** have been isolated and fully characterised. Reaction of the latter with Ni(cod)_2_ yields the corresponding 16 VE nickel(0) complexes, [Si^II^(Terp)Si^II^]Ni(*η*^2^-arene) **5** and [P^III^(Terp)P^III^]Ni(*η*^2^-arene) **4**, respectively. The bis(NHSi)Ni^0^ complex **5** catalyses the homogeneous hydrogenation of olefins under 1 bar H_2_ pressure at room temperature with very good functional group tolerance and excellent chemoselectivity (scope of 22 olefins). In contrast, the bis(phosphine) analogue **4** is far less active. Pre-catalyst **5** is strikingly active because of a Si–Ni cooperativity in H_2_ activation and H atom transfer to the olefin which leads to the highest TON hitherto reported for Ni-based homogeneous hydrogenation of olefins (TON = 9800, TOF up to 6800 h^−1^). The mechanism of olefin hydrogenation could be established experimentally by kinetics, including KIE measurement and Hammet-plot correlation. Application of the pre-catalyst **5** as transfer-hydrogenation catalyst is currently ongoing in our laboratory.

## Conflicts of interest

There are no conflicts to declare.

## Supplementary Material

SC-012-D0SC06471H-s001

SC-012-D0SC06471H-s002
