# Supplementary material for: Boosting homogeneous chemoselective hydrogenation of olefins mediated by a bis(silylenyl)terphenyl-nickel(0) pre-catalyst
Source: Chem Sci. 2021 Jan 8;12(8):2909–15. doi: 10.1039/d0sc06471h (PMC8179395; doi:10.1039/d0sc06471h)
Supplement: SC-012-D0SC06471H-s001 [file SC-012-D0SC06471H-s001.pdf]

Electronic Supplementary Material (ESI) for Chemical Science.
This journal is © The Royal Society of Chemistry 2021

Electronic Supplementary Information

Boosting chemoselective hydrogention of olefins with molecular hydrogen mediated by a bis(silylenyl)terphenyl-nickel(0) pre-catalyst

Marcel-Philip Lücke, Shenglai Yao and Matthias Driess.

1)	General Consideration	S 2
2)	Synthesis and Characterization	S 4
3)	NMR spectra	S 9
4)	UV-VIS spectra	S 17
5)	FT-IR spectra	S 17
6)	ESI-MS spectra	S 19
7)	Cyclic Voltammetry Measurements	S 20
8)	Reversible H_2-activation by bis(silylene)Ni(η^2-arene) 5	S 22
9)	Hydrogenation of Olefins	S 31
10)	Mechanistic study / kinetic analysis	S 45
11)	Crystallographic Data for **2**, **3**, **4**, **5**	S 56
12)	References	S 60

1) General Considerations:

All experiments and manipulations were carried out under dry nitrogen using standard schlenk techniques or inside an MBraun glovebox. Solvents were dried by standard methods and freshly distilled prior to use. The NMR spectra were recorded on a Bruker spectrometers AV200 (^{1}H, 200 MHz; ^{13}C, 50.32 MHz) or AV400 (^{1}H, 400.13 MHz; ^{13}C {^{1}H}, 100.61 MHz; ^{29}Si, 79.49 MHz) spectrometer. The ^{1}H and ^{13}C {^{1}H} spectra were referenced to residual solvent signals as internal standards (^{1}H NMR: benzene-d_6, 7.16 ppm, THF-d_8, 1.72; 3.58 ppm and ^{13}C {^{1}H} NMR: benzene-d_6, 128.1 ppm, THF-d_8, 77.2 ppm). ^{29}Si {^{1}H} spectra were referenced by using $SiMe_4$ as external standard. Concentrated solutions of samples in deuterated solvent were sealed off in a NMR tube for measurements. Abbreviations: s = singlet; t = triplet; m = multiplet; br = broad. Signals were assigned by employing a combination of 2D NMR H,H-COSY, H,C-COSY (HMBC, HMQC) experiments and additional to DEPT experiments. High resolution APCI (atmospheric-pressure chemical ionization) or ESI (electrospray ionization) mass spectra were recorded on an Orbitrap LTQ XL of Thermo Scientific mass spectrometer. Elemental analyses were recorded in a Thermo FlashEA 1112 Organic elemental analyzer. The ATR/IR-spectroscopic Measurements were recorded on a Firma Thermo Fisher Scientific spectrometer inside a glovebox. Vibration Modes are given in wavenumbers (cm^{-1}). Abbreviations: (vs) very strong, (s) strong, (m) middle, (w) weak and (br) broad. Commercially available reagents were purchased from SIGMA-Aldrich, Acros, Alfa-Assar or ABCR and used as received. 1,4-bis(2-bromophenyl)benzene (**1**) was synthesized according to reported procedures.[S1]

Single crystal X-ray structure analyses: Crystals were mounted on a glass capillary in perfluorinated oil and measured in a cold N_2 flow. The data for all compounds were collected on an Agilent Technologies SuperNova (single source) at 150 K (Cu-K_α radiation, λ = 1.5418 Å). All structures were solved by direct methods and refined on F^2 with the SHELX-97 software [S2]. The positions of the H atoms were calculated and considered isotopically according to a riding model. The CCDC numbers 2025191 compound **2**), 2025192 (compound **3**), 2025193 (compound **4**) and 2025194 (compound

5) contain the supplementary crystallographic data for this paper. These data can be obtained free of charge by contacting The Cambridge Crystallographic Data Centre, 12, Union Road, Cambridge CB2 1EZ, UK; fax: +44 1223 336033.

Cyclic Voltammetry Measurement: Cyclic Voltammetry (CV) measurements were carried out at 295 K by using a Biologic SP-150 potentiostat and a three electrode set-up. Pt-wire was used as an auxiliary electrode. A freshly polished glassy carbon disc (3 mm diameter) as a working electrode and a pseudo reference electrode Ag/Ag^+ was used. All cyclic voltammograms were referenced against the Cp_2Fe/Cp_2Fe^+ redox couple which was used as an internal standard. As an electrolyte, 0.3 M solutions of $TBAPF_6$ in THF was used. The iR-drop was determined and compensated by using the impedance measurement technique implemented in the EC-Lab Software V10.

Molecule-Index:

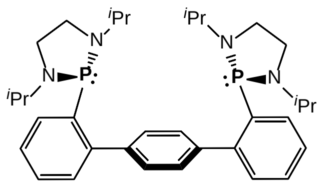

2

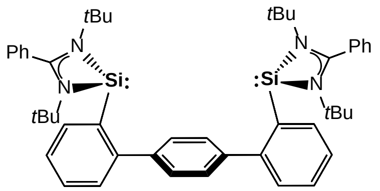

3

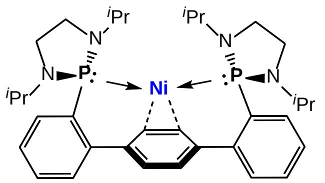

4

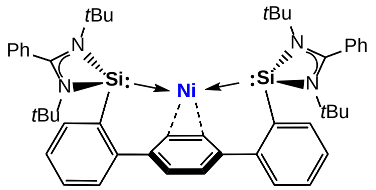

5

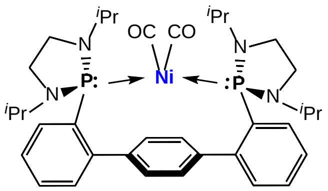

4-CO

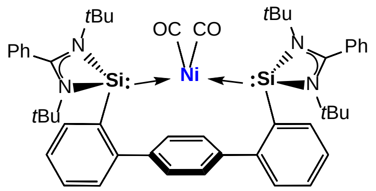

5-CO

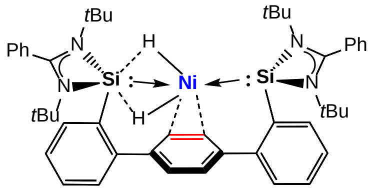

5-H_2

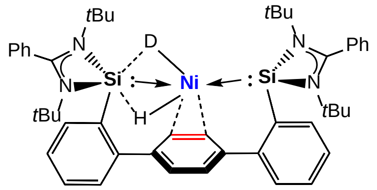

5-HD

2) Synthesis and Characterisation:

Synthesis of 1,4-bis(2-(phosphine)phenyl)benzene (**2**). A mixture of 1,4-bis(2-bromophenyl)benzene (**1**, 200 mg, 0.515 mmol, 1.0 equiv.) and ether (10 mL) was cooled to - 78 °C and a solution of ^{sec}Buli (1.3 M in cyclohexane, 0.9 mL, 1.17 mmol, 2.2 equiv.) was added via syringe. After warming up to room temperature, the solution turned from slight yellow to a suspension containing a white precipitate. After stirring for 4 h at room temperature, the suspension was cooled to - 78 °C and 2-chloro-1,3-diisopropyl-1,3,2-diazaphospholidine (215 mg, 1.03 mmol, 2.0 equiv.) was added via syringe. After stirring for 1 h at room temperature, all volatiles were removed under reduced pressure and dissolved in 10 mL Et_2O. After the solution was filtered and reduced to a volume of 1.0 ml, the solution was kept at -30 °C over night affording **2** as colorless crystals. After removal of Et_2O, crystals were washed with 0.5 mL cold Et_2O (-78 °C) to give **2** in 82 % (242 mg, 0.40 mmol) isolated yields. Single crystals suitable for an x-ray diffraction analysis were obtained from a concentrated hexane solution at room temperature overnight. Compound **2** is air stable and is well soluble in general organic solvents (Et_2O, hexane, THF, toluene).

^{1}H NMR (200.13 MHz, C_6D_6, 298 K): δ/ppm = 1.00 (d, $^3J_{HH}$ = 6.5 Hz, 12 H, 2 x CH(CH_3)$_2$, 1.20 (d, $^3J_{HH}$ = 6.5 Hz, 12 H, 2 x CH(CH_3)$_2$, 2.62 – 2.82 (mbr, 4 H, N(CH_2)$_2$N), 2.85 – 3.01 (mbr, 4 H, N(CH_2)$_2$N), 3.02 – 3.29 (m, 4 H, 4 x C*H*(CH$_3$)),7.20 – 7.38 (m, 6 H, Ar*H*), 7.71 (s, 4 H, Ar*H*), 7.86 – 7.96 (m, 2 H, Ar*H*). **^{1}H{^{31}P } NMR** (200.13 MHz, C_6D_6, 298 K): δ/ppm = 1.00 (d, $^3J_{HH}$ = 6.5 Hz, 12 H, 2 x CH(CH_3)$_2$, 1.20 (d, $^3J_{HH}$ = 6.5 Hz, 12 H, 2 x CH(CH_3)$_2$, 2.83 (dm, 8 H, 4 x N(CH_2)$_2$N), 3.17 (sept, $^3J_{HH}$ = 6.4 Hz, 4 H, 4 x C*H*(CH$_3$)), 7.20 – 7.38 (m, 6 H, Ar*H*), 7.71 (s, 4 H, Ar*H*), 7.86 – 7.96 (d, 2 H, $^3J_{H,H}$ = 6.9 Hz, Ar*H*). **^{13}C NMR** (125 MHz, C_6D_6, 298 K): δ/ppm = 22.8 (s, 2 x CH(CH$_3$)$_2$), 22.9 (s, CH(CH$_3$)$_2$), 22.9 (s, CH(CH$_3$)$_2$), 47.9 (d, $^2J_{C,P}$ = 7.0 Hz, 4 x N(CH$_2$)$_2$N), 51.0 (d, $^2J_{C,P}$ = 25.3 Hz, 4 x CH(CH$_3$)$_2$), 126.2 (s, C_{Ar}), 128.4 (s, C_{Ar}), 130.3 (d, $^3J_{C,P}$ = 4.5 Hz, C_{Ar}), 130.7 (d, J = 3.6 Hz, C_{Ar}), 130.9 (brs, C_{Ar}), 141.4 (d, $^3J_{C,P}$ = 3.6 Hz, C_{quart}), 144.0 (d, $^1J_{C,P}$ = 35.3 Hz, C_{quart}), 146.3 (d, $^2J_{C,P}$ = 23.5 Hz, C_{quart}). **^{31}P{^{1}H} NMR** (81.00 MHz, C_6D_6, 298 K): δ/ppm = 92.55 (s, satellite: $J_{P,C}$ = 25.5 Hz). **FT-IR** (solid): $\tilde{\nu}$/cm^{-1} =3066 (w), 3047

(w), 2962 (s), 2920 (s), 2874 (s), 2841 (s), 1453 (s), 1301 (s), 1302 (s), 1210 (s), 1158 (s), 1041 (s), 1004 (s), 982 (s), 847 (s), 756 (vs). **ESI-MS** m/z (%): Calcd. for $[M+H]^+$ = $C_{34}H_{49}N_4P_2^+$: 575.3427, found. 575.3430. **Melting Point** T/°C: 102. **Elemental analysis**: Calcd. for $C_{34}H_{48}N_4P_2$: C, 71.05; H, 8.42; N, 9.75. Found: C, 71.23; H, 8.34; N, 9.43.

Synthesis of 1,4-bis(2-(silylene)phenyl)benzene (**3**). A diethylether suspension (20 mL) of 1,4-bis(2-bromophenyl)benzene (**1**, 500 mg, 1.29 mmol, 1.0 equiv.) was cooled to - 78 °C and a solution of ^{sec}Buli (1.3 M in cyclohexane, 2 mL, 2.58 mmol, 2.0 equiv.) was added via syringe. After warming up to room temperature, the solution turned from slight yellow to a suspension containing a white precipitate. After stirring for 4 h at room temperature, the suspension was cooled to - 78 °C and a solution (Et_2O, 10 mL) of N,N′-di-tert-butyl(phenylamidinato)-phenylsilylene (750 mg, 2.58 mmol, 2.0 equiv.) was added via syringe. The cooling bath was removed and the suspension was stirred at room temperature over night to give a yellow suspenstion. Volatiles were removed under reduced pressure and the residue was washed twice with 4 mL Et_20. The title compound was extracted using hot toluene (60°C, 3 x 50 ml) to give a yellow solution. After evaporation, **3** was isolated as pale yellow crystalline material in 68 % (650 mg, 0.87 mmol) isolated yields. Single crystals suitable for an xray diffraction analysis were obtained from a concentrated toluene solution at – 30 °C after 3 days. Compound **3** is not air stable and is generally not well soluble (THF > Et_2O, toluene).

^{1}H NMR (200.13 MHz, C_6D_6, 298 K): *δ*/ppm = 1.03 (s, 36 H, 4 x NC(C*H*$_3$)$_3$) 6.90 - 7.03 (m, 6 H, Ar*H*), 7.26 - 7.57 (m, 10 H, Ar*H*), 7.85 – 7.95 (m, 2 H, Ar*H*), 8.02 (s, 4H, Ar*H*$_{central}$). **^{13}C NMR** (125 MHz, C_6D_6, 298 K): δ/ppm = 31.6 (s, 4 x NC(*C*H$_3$)$_3$), 53.1 (s, 4 x N*C*$_{quart}$(CH$_3$)$_3$), 125.7 (s, 2 x Ar*C*), 127.4 (s, 2 x Ar*C*), 127.9 (s, 2 x Ar*C*), 128.1 (s, 2 x Ar*C*), 128.7 (s, 2 x Ar*C*), 128.9 (s, 2 x Ar*C*), 129.3 (s, 2 x Ar*C*), 130.7 (s, 2 x Ar*C*), 130.9 (s, 2 x Ar*C*), 135.1 (s, 2 x Ar*C*$_{quart}$), 142.6 (s, 2 x Ar*C*$_{quart}$), 145.2 (s, 2 x Ar*C*$_{quart}$), 152.3 (s, 2 x Ar*C*$_{quart}$), N*C*N not detected. **^{29}Si NMR** (79 MHz, C_6D_6, 298 K): +16.8 ppm (s). **FT-IR** (solid): $\tilde{\nu}$/cm^{-1} = 3058 (w), 3041 (w), 2970 (s), 2928 (w), 2901 (w), 2885 (w), 1645 (w), 1612 (w), 1579 (w), 1416 (vs), 1663 (s), 1236 (s), 757 (vs). 616 (vs). **APCI-MS**

m/z (%): Calcd. for $[M+OH]^+ = C_{48}H_{59}N_4Si_2O^+$: 763.4222, found. 763.4230. **Melting Point** T/°C: 240 (decomp.). **Elemental analysis:** Calcd. for $C_{48}H_{58}N_4Si_2$: C, 77.16; H, 7.82, N, 7.50. Found: C, 73.34; H, 7.36; N, 7.23. (Reduced C content due to SiC formation)

Synthesis of [1,4-bis(2-(phosphine)phenyl)benzene]nickel(0) (**4**). A mixture of 1,4-bis(2-(phosphine)phenyl)benzene (**2**, 100 mg, 0.17 mmol, 1.0 equiv) and $Ni(cod)_2$ (48 mg, 0.17 mmol, 1.0 equiv.) was dissolved in 10 mL Et_2O and stirred for 4 h at room temperature upon which a dark red solution is formed. Then, all volatiles were removed and the title compound was extracted with 5 ml hexane. After reduction of the volume to 0.5 ml **4** could be obtained as dark red crystals in 84 % isolated yields (92 mg, 0.14 mmol) after 3 days at – 30 °C. Single crystals suitable for an x-ray diffraction analysis were obtained from a concentrated hexane solution at – 30 °C. Compound **2** is not air stable and is well soluble in general organic solvents (Et_2O, hexane, THF, toluene, benzene). Complex **4** shows line-broadening in the ^{1}H-NMR at 298 K. ^{1}H, ^{13}C-NMR resonances of the coordinated arene were resolved at 233 K and assigned by a ^{1}H,^{13}C-HSQC NMR spectra.

^{1}H NMR (200.13 MHz, C_6D_6, 298 K): δ/ppm = 0.87 (d, $^3J_{HH}$ = 5.1 Hz, 12 H, 2 x $CH(CH_3)_2$, 1.07 (d, $^3J_{HH}$ = 6.5 Hz, 12 H, 2 x $CH(CH_3)_2$, 2.86 (mbr, 4 H, $N(CH_2)_2N$), 3.13 (mbr, 4 H, $N(CH_2)_2N$), 3.57 (m, 4 H, 4 x $CH(CH_3)$), 5.60 (br, 2H, central Aryl*H*), 7.17 – 7.36 (m, 6 H, Ar*H*), 7.47 – 7.60 (s, 2 H, Ar*H*), 7.67 – 7.78 (m, 2 H, Ar*H*). **^{13}C NMR** (175 MHz, C_6D_6, 298 K): δ/ppm = 21.2 (s, 2 x $CH(CH_3)_2$), 22.0 (s, 2 x $CH(CH_3)_2$), 42.9 (br , 4 x $N(CH_2)_2N$), 47.1 (br, 4 x $CH(CH_3)_2$), 126.2 (s, C_{Ar}), 127.0 (s, C_{Ar}), 129.1 (s, C_{Ar}), 128.2 (s, C_{Ar}), 128.2 (s, C_{Ar}), 130.6 (s, C_{Ar}), 132.1 (t, $^3J_{C,P}$ = 2.3 Hz, C_{quart}), 138.9 (t, $^2J_{C,P}$ = 7.4 Hz, C_{quart}), 148.7 (t, $^1J_{C,P}$ = 10.2 Hz, C_{quart}). **^{1}H,^{13}C-HSQC** (125 MHz, d_8-THF, 233 K): 4.7/ 62.4 ($Ni\text{-}C_2H_2$) 6.2/ 120.9 (HC_{Ar}). **^{31}P{^{1}H} NMR** (81.00 MHz, C_6D_6, 298 K): δ/ppm = 103.6 (s). **FT-IR** (solid): λ/cm^{-1} = 3043 (w), 2961 (s), 2925 (w), 2827 (s), 1583 (w), 1451 (s), 1357 (s), 1172 (vs), 1050 (vs), 955 (vs), 806 (vs), 742 (vs), 694 (vs). **UV/VIS** (THF): $\tilde{\nu}$/nm = 408. **CV:** $E^{1/2}$ (fc/fc$^+$) = -0.938 (Ni^0/Ni^I); -0.472 (Ni^I/Ni^{II}) V (revers.). **ESI-MS** m/z (%): Calcd. for $[C_{34}H_{48}N_4P_2Ni + H]^+$: 633.2780, found. 633.2781. **Melting Point** T/°C: 142. **Elemental**

analysis: Calcd. for $C_{34}H_{48}N_4P_2Ni$: C: 64.47, H: 7.64, N: 8.85. Found: C, 64.12; H, 7.33; N, 8.34.

Synthesis of [1,4-bis(2-(silylene)phenyl)benzene]nickel(0) (**5**). A mixture of 1,4-bis(2-(silylene)phenyl)benzene (**2**, 200 mg, 0.27 mmol, 1.0 equiv) and $Ni(cod)_2$ (73.6 mg, 0.27 mmol, 1.0 equiv.) was dissolved in 4 mL Et_2O and stirred for 4 h at room temperature upon which dark red crystals are formed. The suspension was cooled to -78 °C and the upper solution was removed via cannula filtration. After additional washing with 0.7 ml cold Et_2O (-78 °C) Ni(0) complex **5** was isolated in 64 % (140 mg, 0.17 mmol) as small dark-red crystals. Single crystals suitable for an x-ray diffraction analysis were obtained from a concentrated toluene solution at – 30 °C. Compound **2** is not air stable and smokes off to a black solid. **2** is highly soluble in THF and toluene > Et_2O > benzene. Complex **5** shows line-broadening in the ^{1}H-NMR at 298 K. ^{1}H,^{13}C-HSQC NMR resonances of the coordinated arene were resolved at 233 K and assigned by a ^{1}H,^{13}C-HSQC NMR spectra.

^{1}H NMR (500 MHz, d_8-THF, 298 K): *δ*/ppm = 1.11 (br s, 36 H, 4 x $NC(CH_3)_3$), 4.21 (br s, 2 H, Ar$H_{central}$), 5.91 (br s, 2 H, Ar$H_{central}$), 7.22 - 7.29 (m, 4 H, Ar*H*), 7.35 - 7.40 (m, 4 H, Ar*H*), 7.50 - 7.58 (m, 8 H, Ar*H*), 7.76 – 7.78 (s, 2H, Ar*H*). **^{13}C NMR** (125 MHz, d_8-THF, 298 K): δ/ppm = 32.2 (s, 4 x $NC(CH_3)_3$), 54.4 (s, 4 x $NC_{quart}(CH_3)_3$), 126.3 (s, 2 x Ar*C*), 126.5 (s, 2 x Ar*C*), 128.8 (s, 2 x Ar*C*), 128.8 (s, 2 x Ar*C*), 129.0 (s, 2 x Ar*C*), 129.1 (s, 2 x Ar*C*), 130.8 (s, 2 x Ar*C*), 131.6 (s, 2 x Ar*C*), 131.7 (s, 2 x Ar*C*), 135.1 (s, 2 x ArC_{quart}), 135.6 (s, 2 x ArC_{quart}), 137.0 (s, 2 x ArC_{quart}), 155.1 (s, 2 x ArC_{quart}), 166.5 (s, 2 x C_{quart}, N*C*N). **^{1}H,^{13}C-HSQC** (125 MHz, d_8-THF, 233 K): 4.14/ 54.5 (Ni-C_2H_2) 6.00/ 118.6 (HC_{Ar}). **^{29}Si NMR** (79 MHz, C_6D_6, 298 K): + 51.0 ppm (s). **FT-IR** (solid): λ/cm^{-1} = 3042 (w), 2971 (s), 2930 (w), 2867 (w), 1579 (s), 1417 (vs), 1389 (s), 1277 (s), 1236 (vs) 754 (vs), 707 (vs), 624 (vs). **UV/VIS** (THF): $\tilde{\nu}$ /nm = 442. **CV:** $E^{1/2}$ (fc/fc$^+$) = -1.341 (Ni^0/Ni^I); - 1.227 (Ni^I/Ni^{II}) V (revers.). **ESI-MS** m/z (%): Calcd. for $[M+H]^+$ = $C_{48}H_{59}N_4Si_2Ni^+$: 805.3627, found. 805.3626. **Melting Point** T/°C: > 300 (decomp.). **Elemental analysis:** Calcd. for $C_{48}H_{58}N_4Si_2Ni$: C, 71.54; H, 7.25; N, 6.95. Found: C, 68.20; H, 7.13; N, 6.63.

Synthesis of $[E(Terp)E]Ni(CO)_2$ (E = P,Si). A J. Young NMR tube was charged with 4 mg of the Ni(0) complex and 0.45 mL THF was added into the NMR tube. After three freeze-pump-thaw cycles, the solution was subjected to 1 bar CO gas at room temperature with an immediate color change from dark red to pale yellow. After 2 minutes at room temperature, volatiles were removed under reduced pressure and the obtained pale yellow solid was subjected to an FT-IR spectroscopic analysis revealing the formation of the corresponding $[Ni]CO_2$ complexes **4-CO** and **5-CO** as indicated by two very strong IR-absorption bands. The other minor absorption band, which is observed for both complexes (**4-CO** and **5-CO**) possibly corresponds to the related 18VE Tricarbonylcomplex which is formed in small quantities in the presence of an excess CO.

Characterization by FT-IR spectroscopy (solid, cm^{-1}, S1**):**

4-CO: $\tilde{\nu}$ **=** 1997, 1939 (vs, CO).

5-CO: $\tilde{\nu}$ **=** 1970, 1881 (vs, CO).

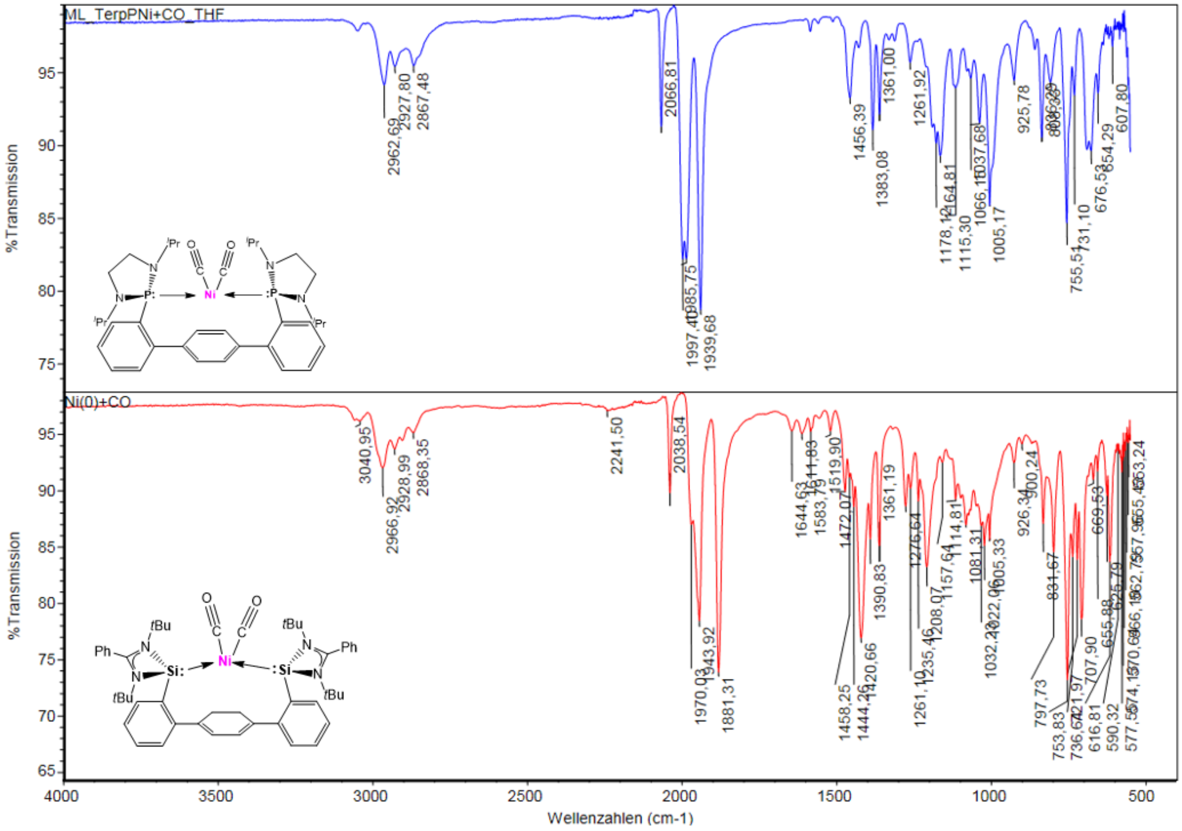

Fig. S1: FT-IR spectra of **4-CO** (top) and **5-CO** (bottom).

3) NMR spectra:

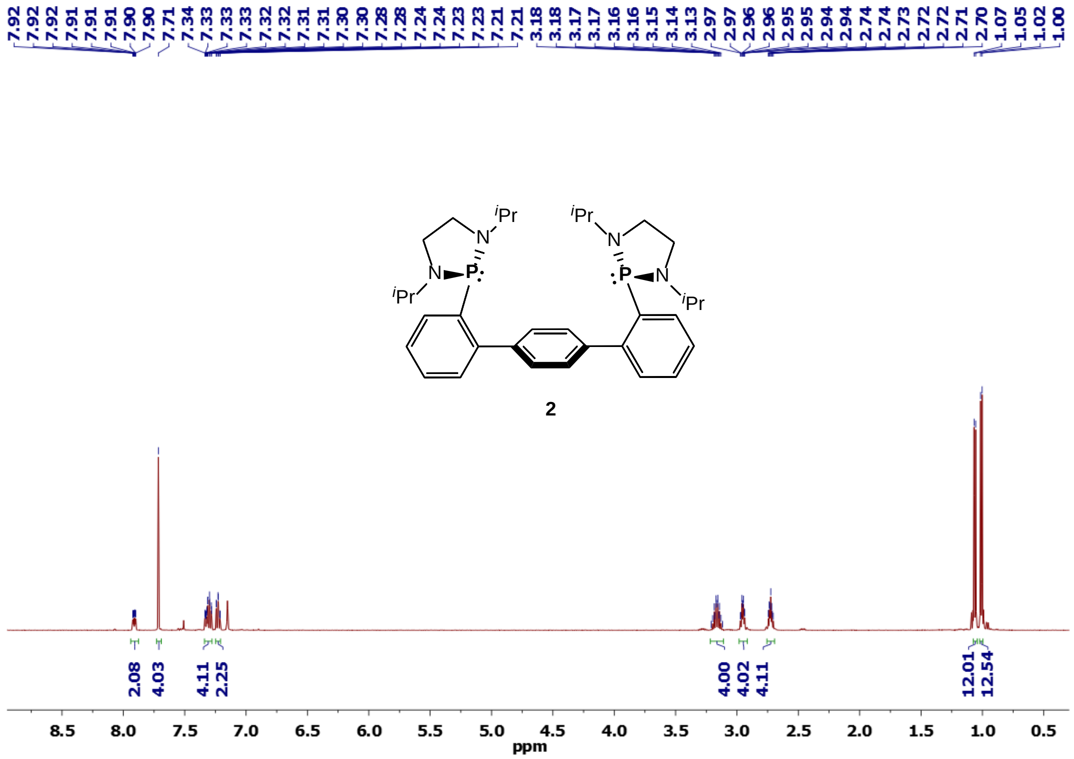

Fig. S2: ^{1}H NMR spectrum of bis(phosphine) **2** in C_6D_6 at 298 K.

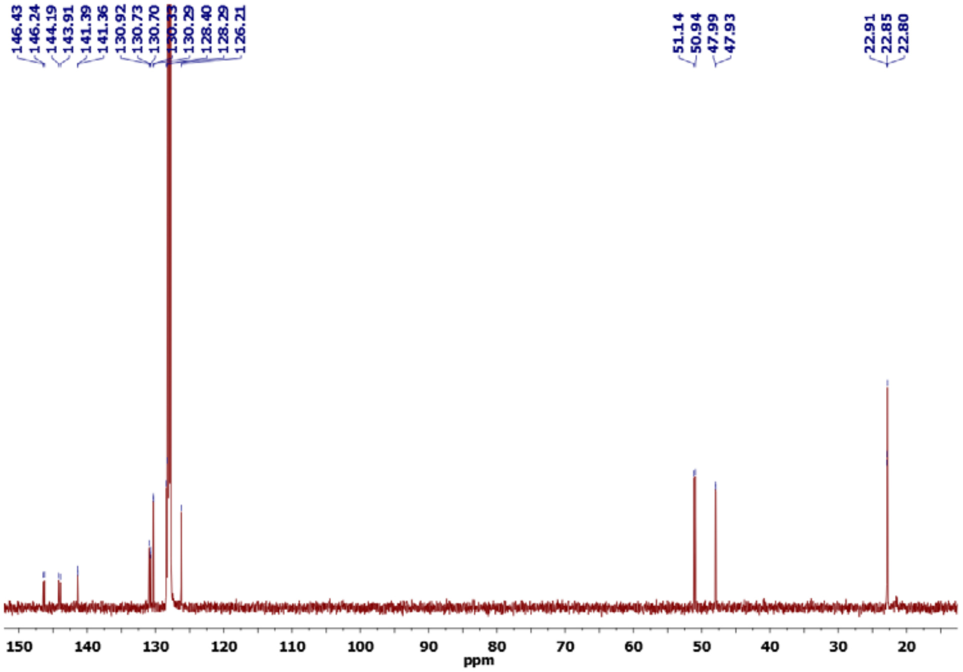

Fig. S3: ^{13}C NMR spectrum of bis(phosphine) **2** in C_6D_6 at 298 K.

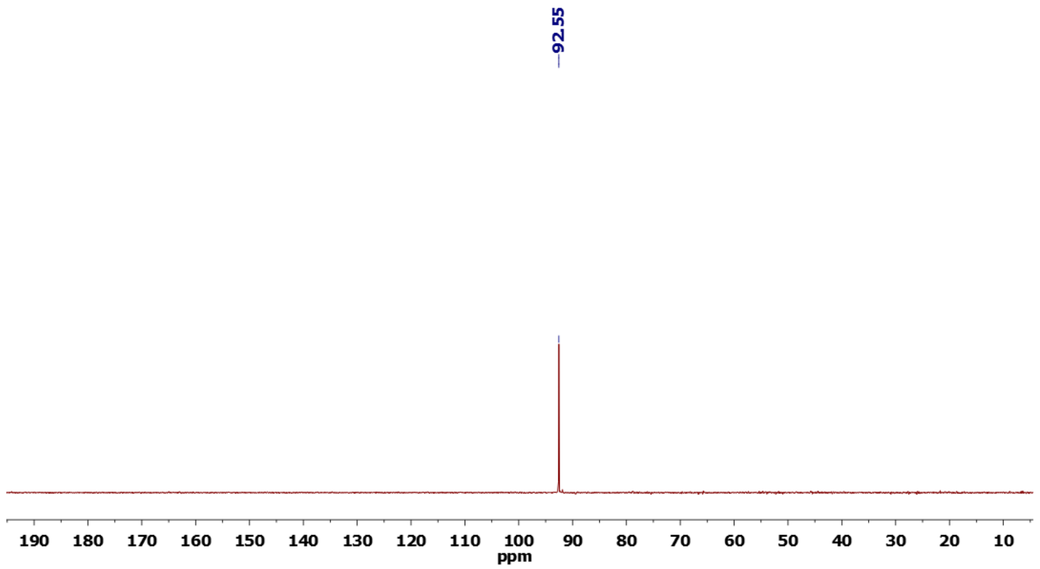

Fig. S4: ^{31}P NMR spectrum of bis(phosphine) **2** in C_6D_6 at 298 K.

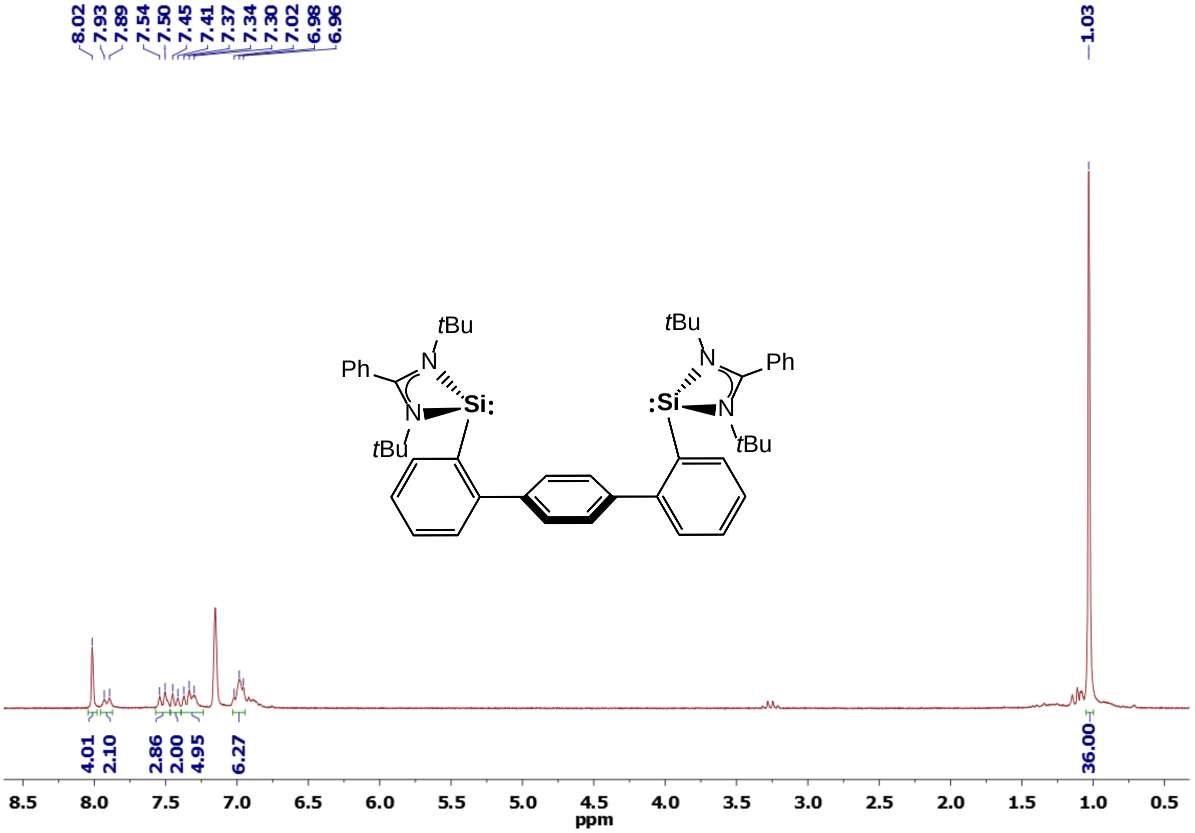

Fig. S5: ^{1}H NMR spectrum of bis(silylene) **3** in C_6D_6 at 298 K.

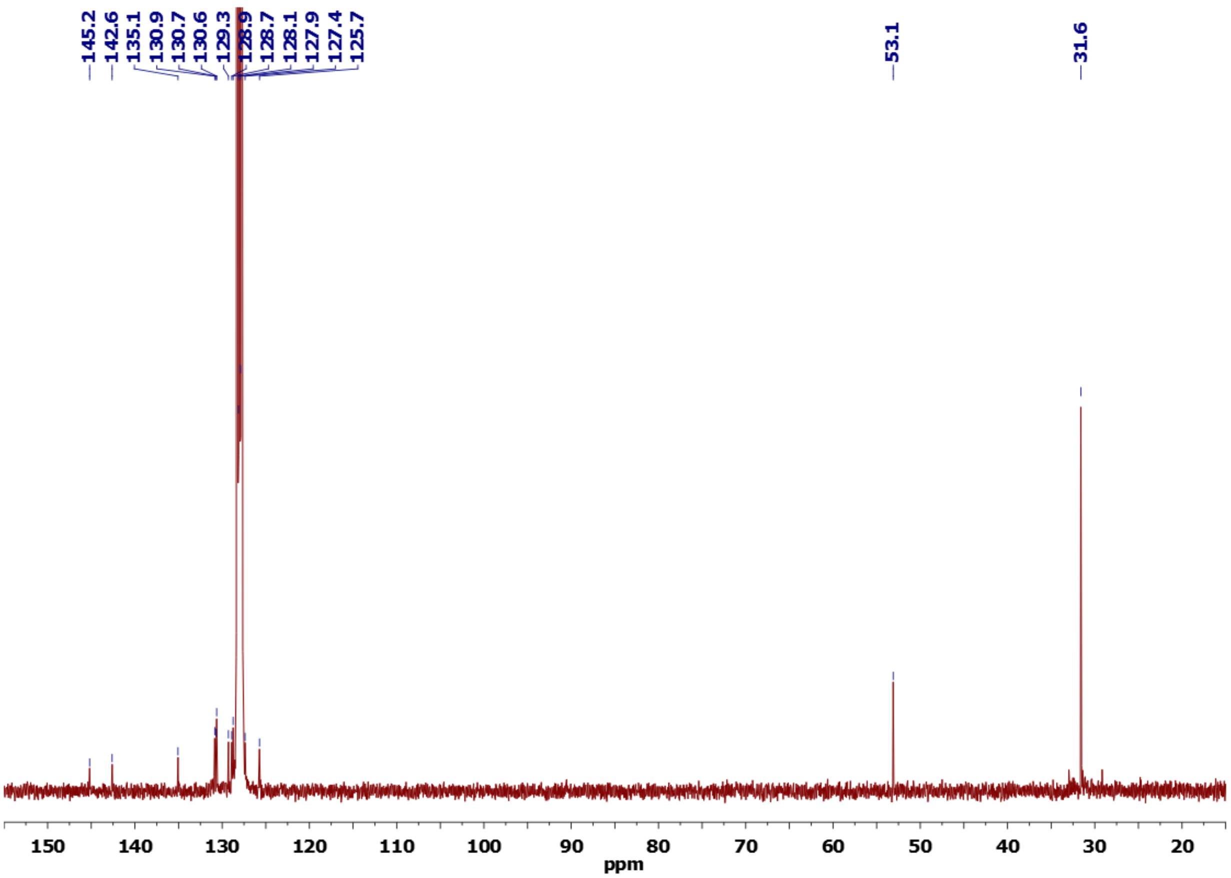

Fig. S6: ^{13}C NMR spectrum of bis(silylene) **3** in C_6D_6 at 298 K.

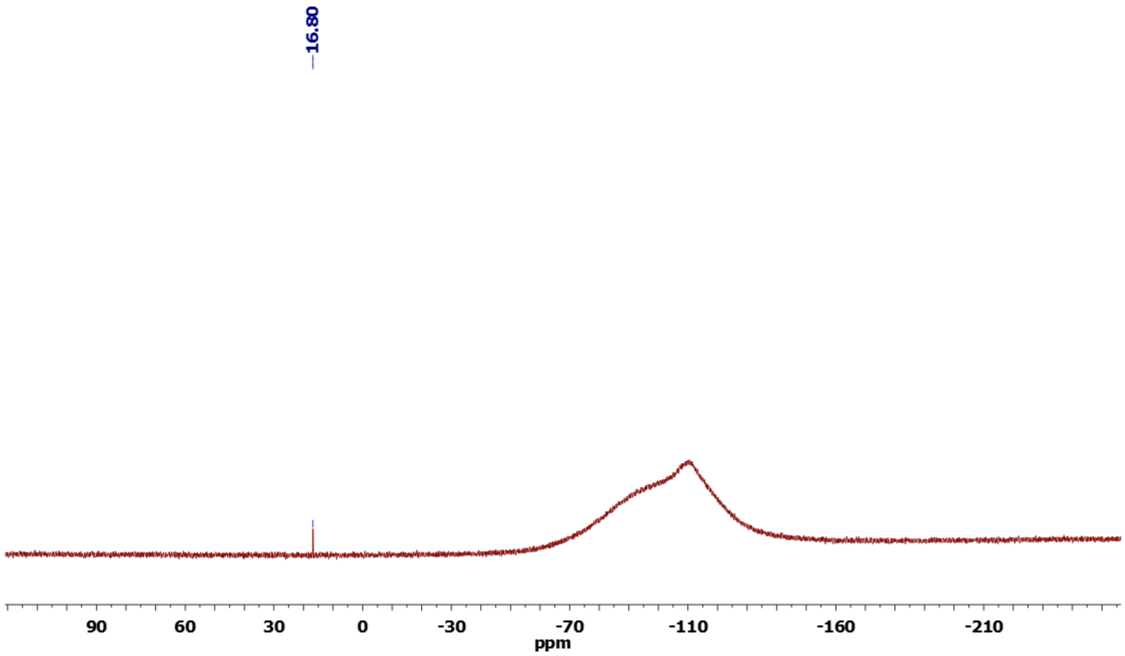

Fig. S7: ^{29}Si NMR spectrum of bis(silylene) **3** in C_6D_6 at 298 K.

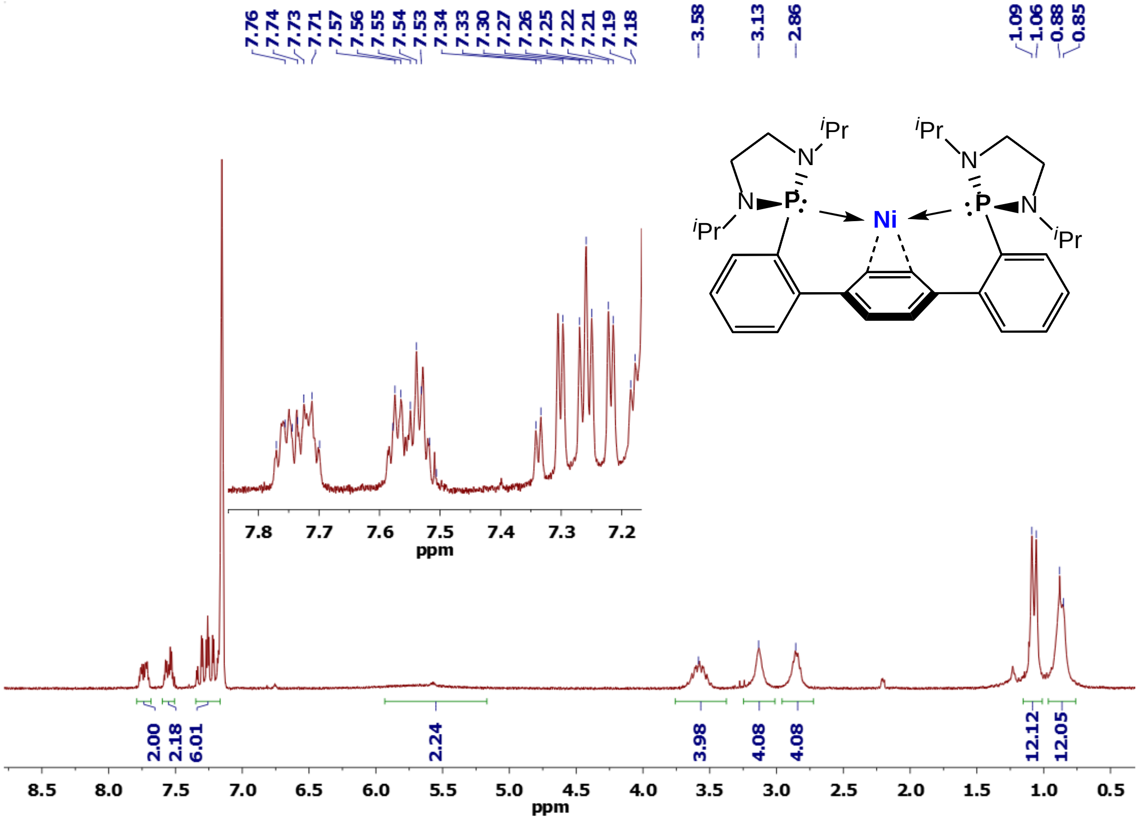

Fig. S8: ^{1}H NMR spectrum of Ni(0) complex **4** in C_6D_6 at 298 K.

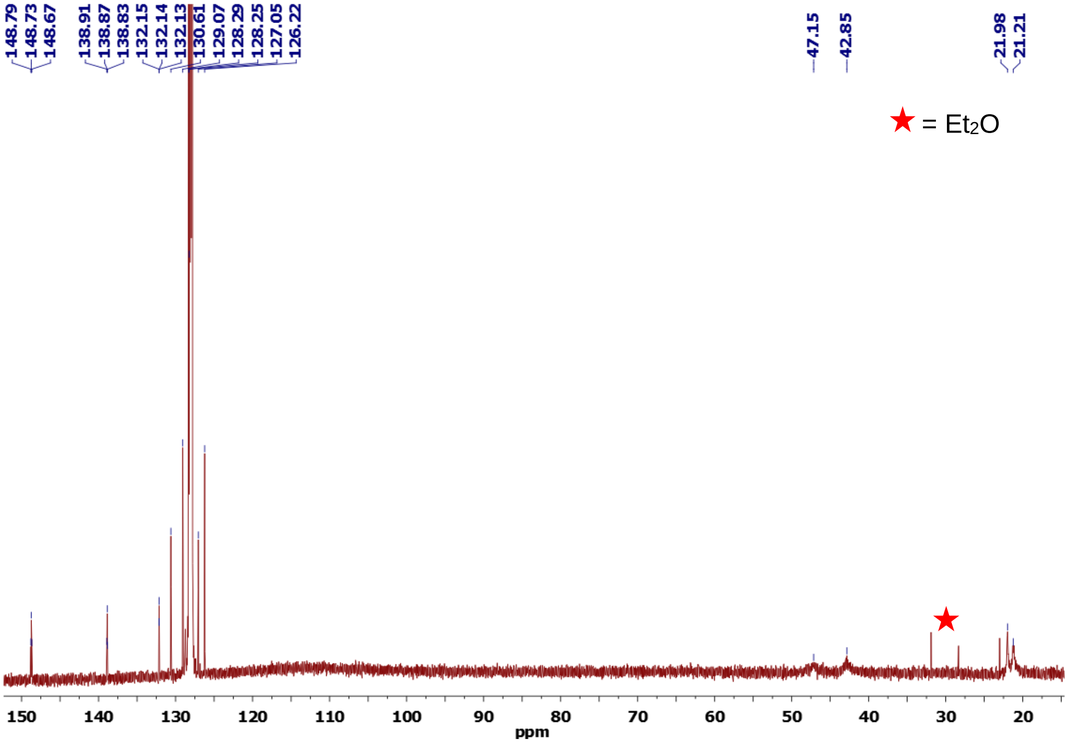

Fig. S9: ^{13}C NMR spectrum of Ni(0) complex **4** in C_6D_6 at 298 K .

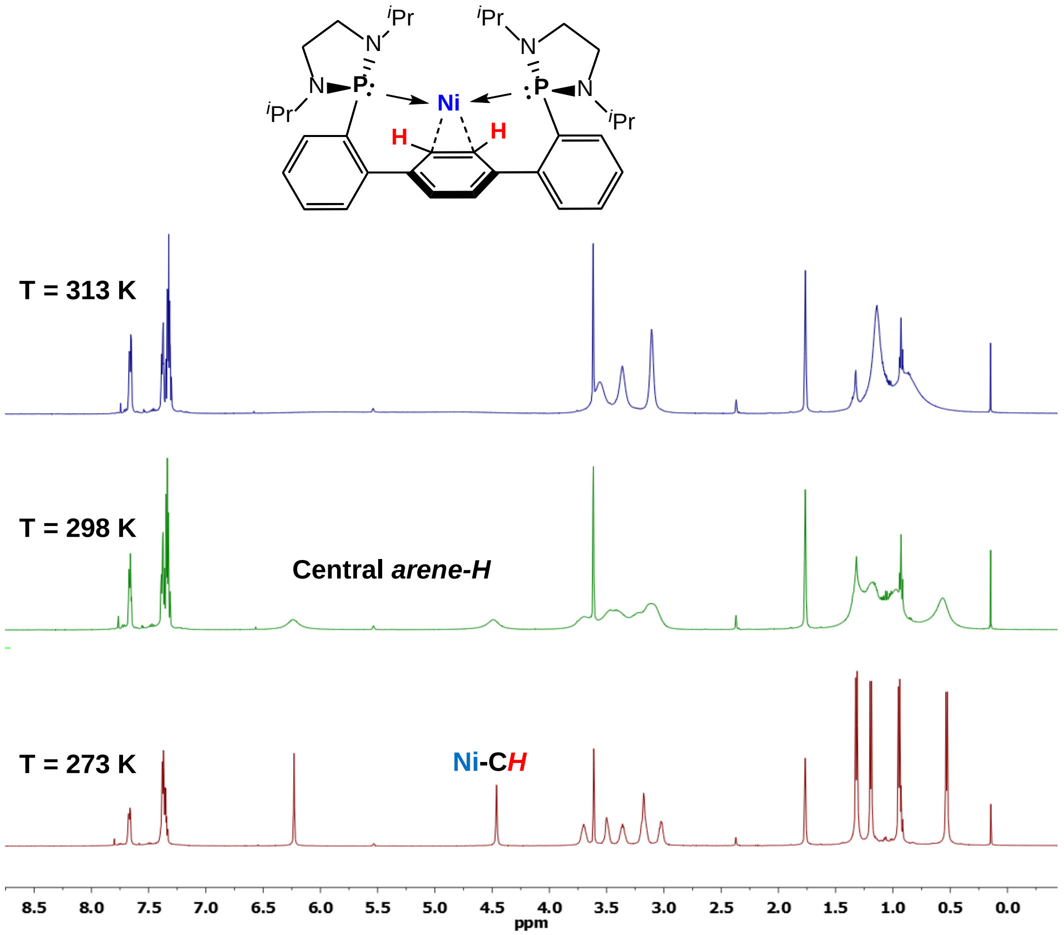

Fig. S10: VT-^{1}H NMR spectrum of Ni(0) complex **4** at variable temperature in d_8-THF (500 MHz).

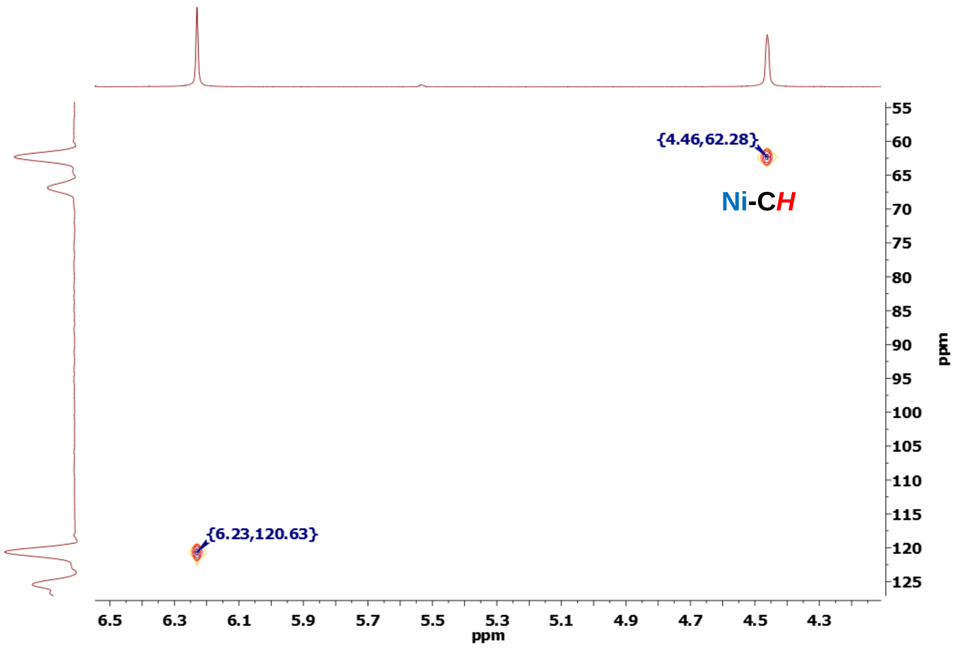

Fig. S11: ^{1}H,^{13}C-HSQC NMR spectrum of Ni(0) complex **4** in d_8-THF at 233 K .

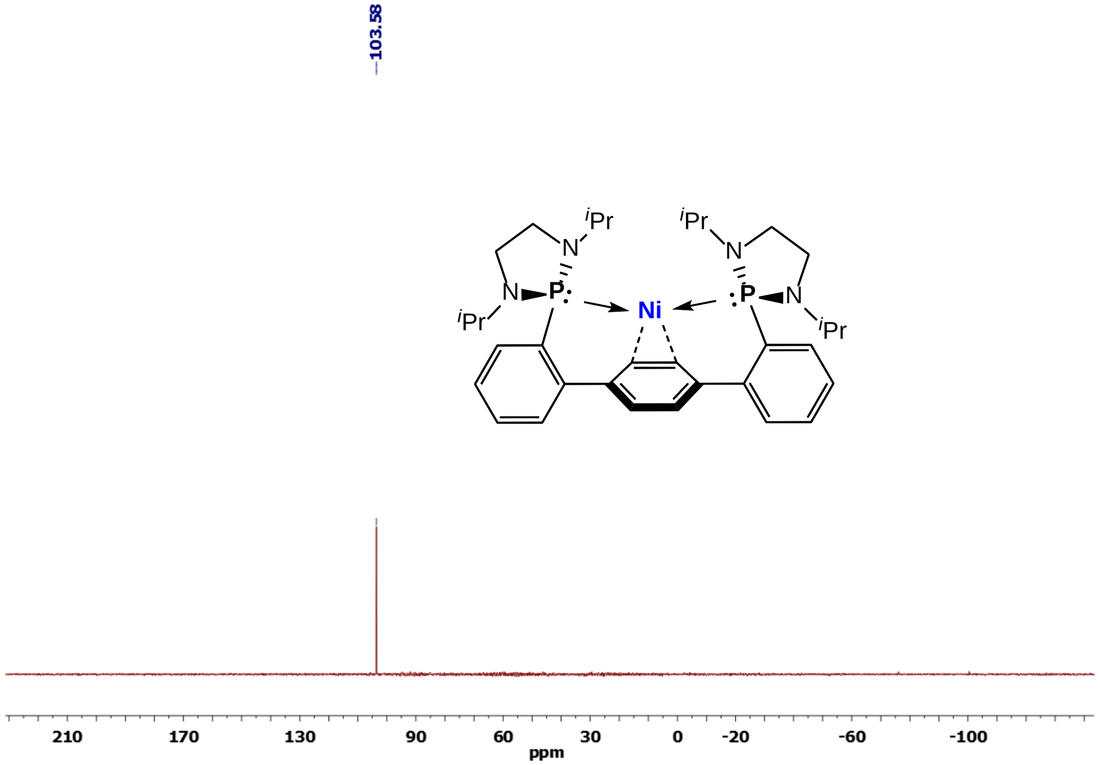

Fig. S12: $^{31}P\{^{1}H\}$ NMR spectrum of Ni(0) complex **4** in C_6D_6 at 298 K.

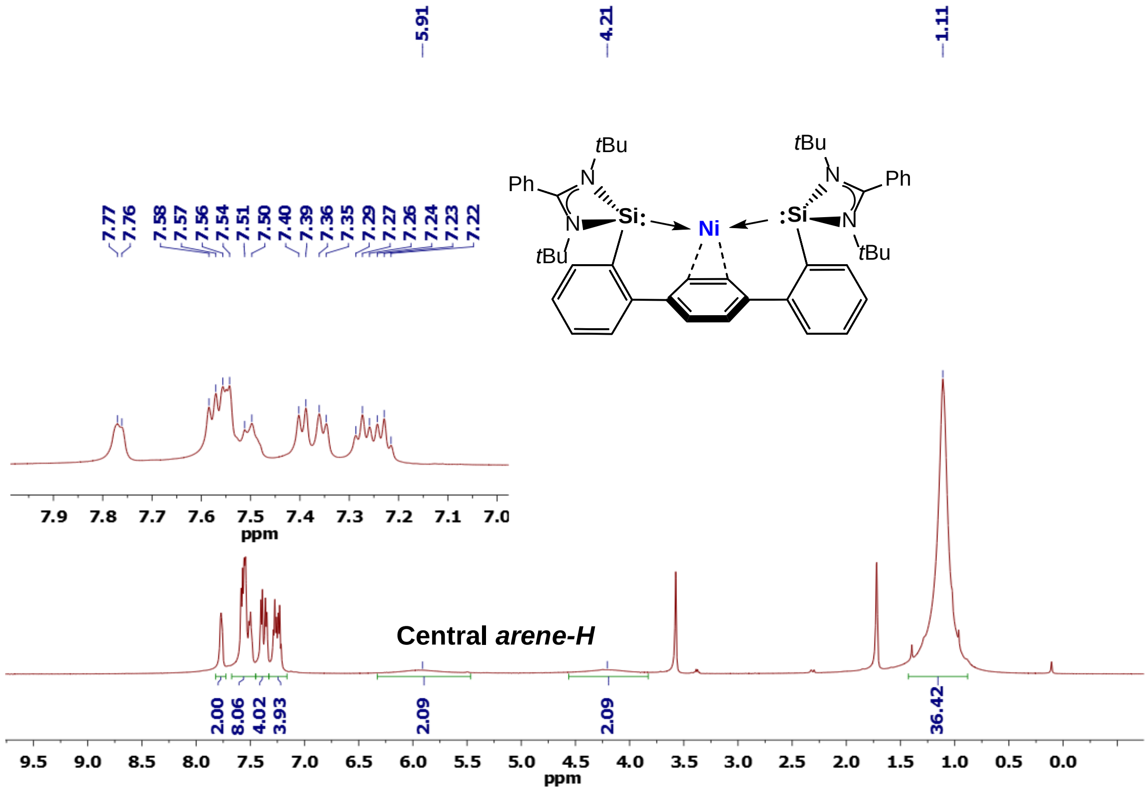

Fig. S13: ^{1}H NMR spectrum of Ni(0) complex **5** in d_8-THF at 298 K.

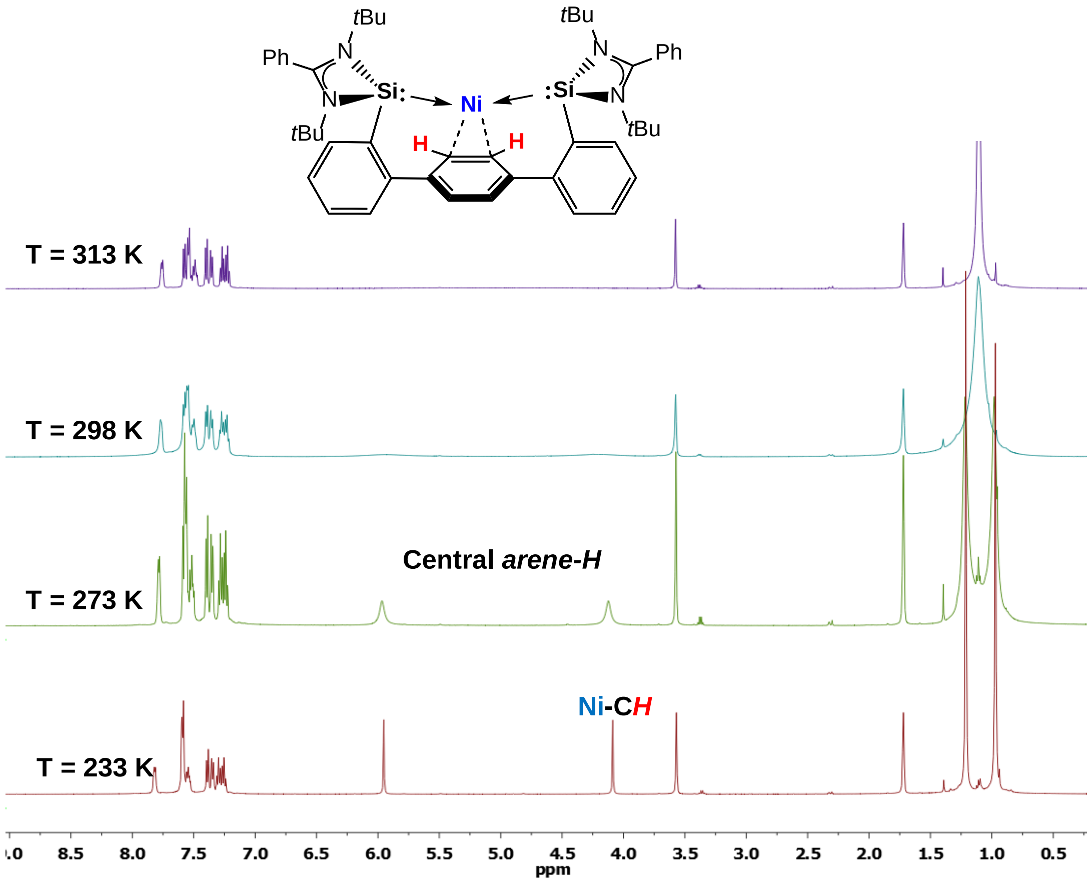

Fig. S14: VT-¹H NMR spectrum of Ni(0) complex **5** at variable temperature in d_8-THF (500 MHz).

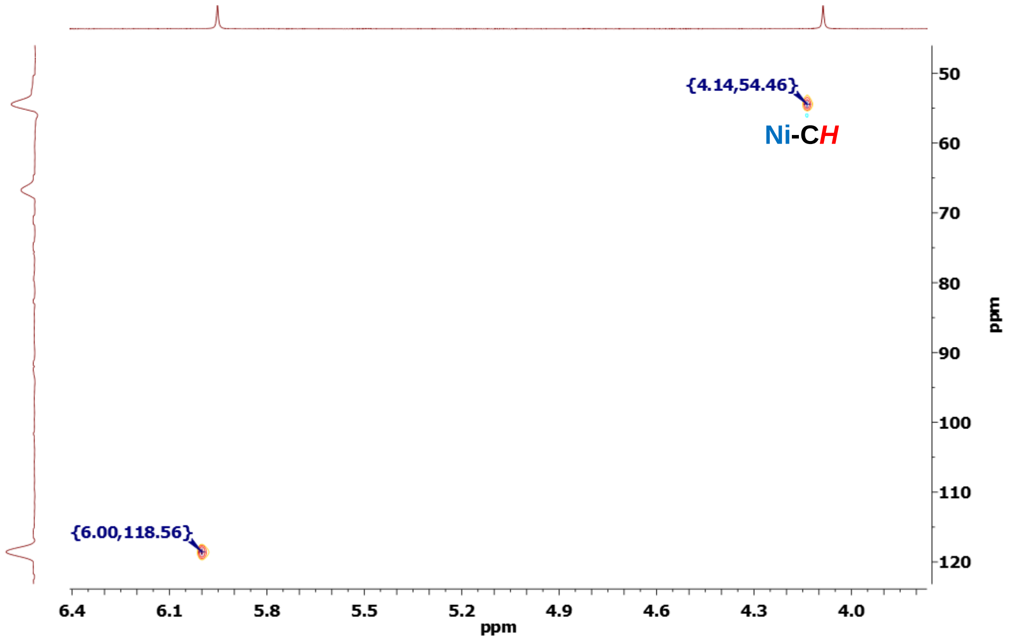

Fig. S15: ¹H,¹³C-HSQC NMR spectrum of Ni(0) complex **5** in d_8-THF at 233 K .

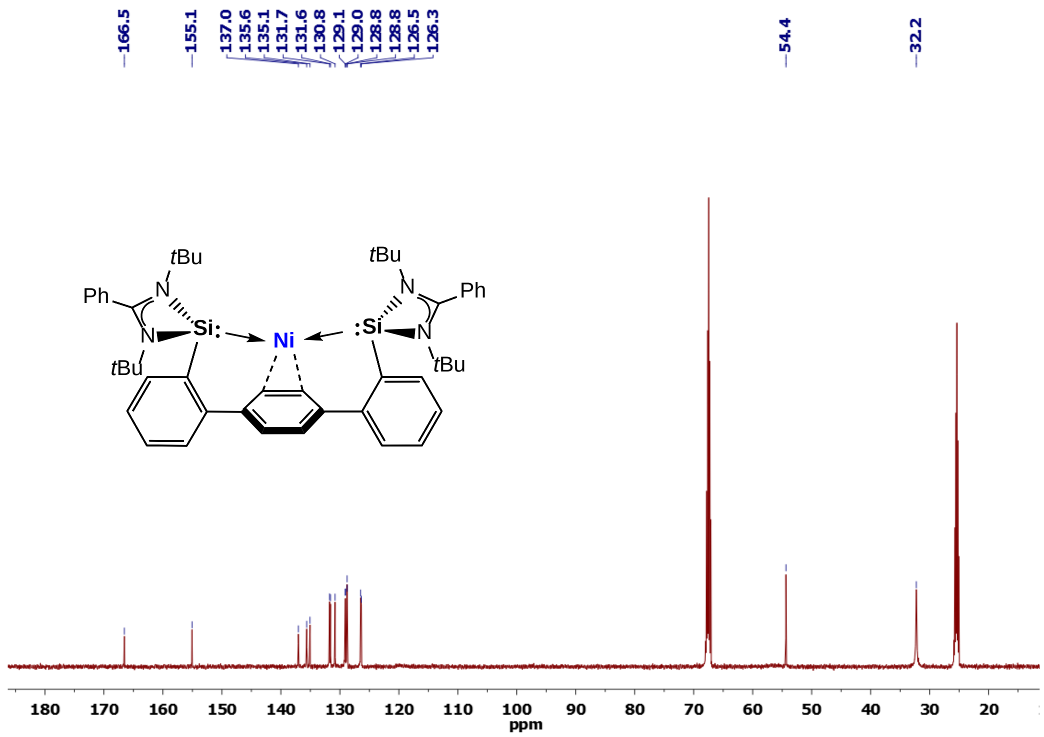

Fig. S16: ^{13}C NMR spectrum of Ni(0) complex **5** in d_8-THF at 298 K .

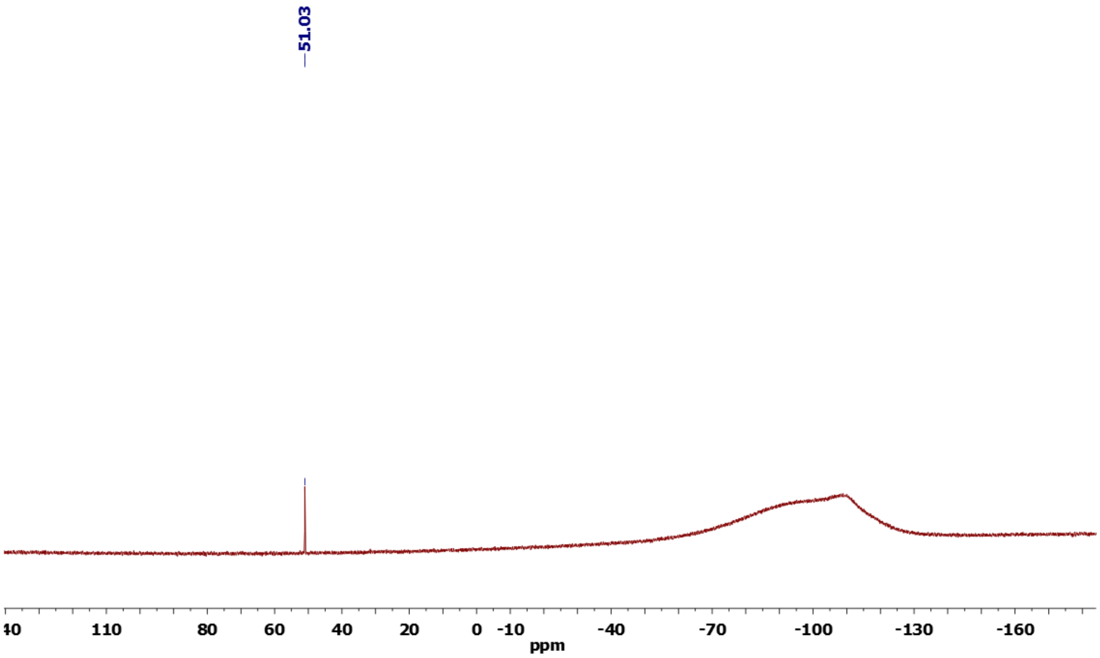

Fig. S17: ^{29}Si NMR spectrum of Ni(0) complex **5** in d_8-THF at 298 K.

4) UV/VIS Spectra:

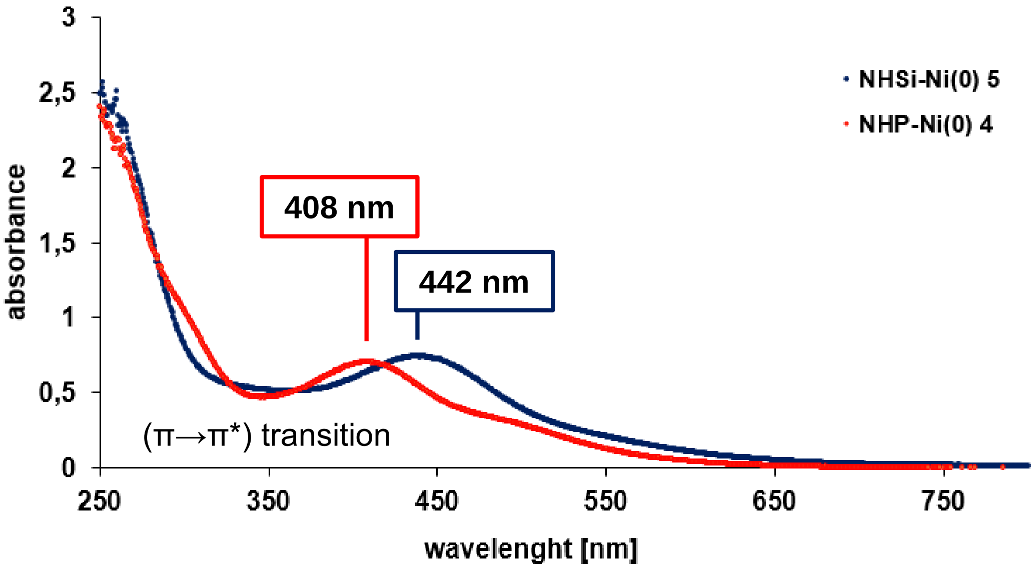

Fig. S18: UV/ VIS spectra recorded in THF at 298 K of **4** and **5**.

Compound	λ_{max} [nm]
Bis(phosphine) Ni(0)-complex **4**	**408**
Bis(silylene) Ni(0)-complex **5**	**442**

5) FT-IR Spectra:

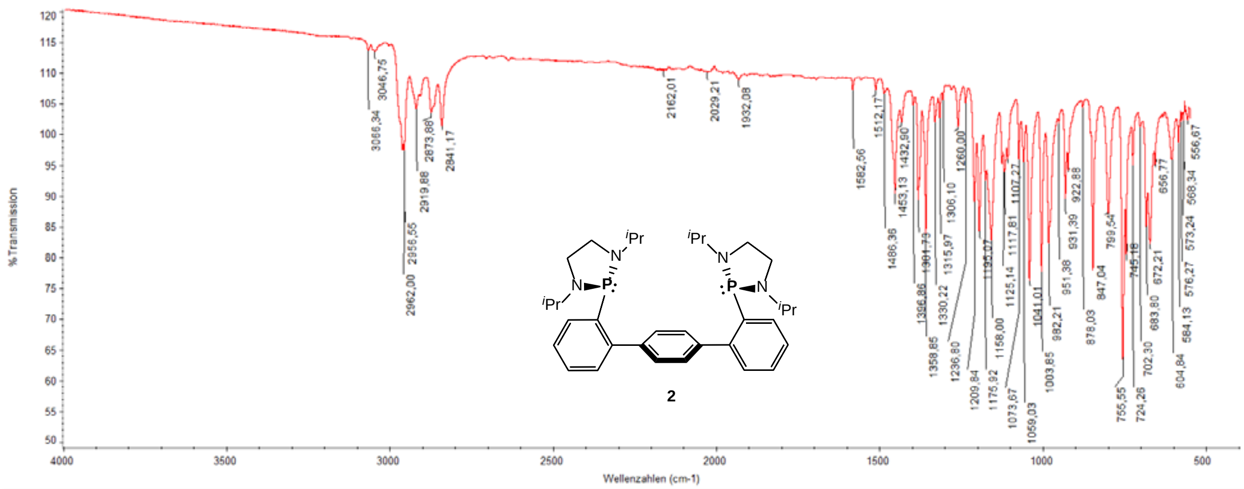

Fig. S19: FT-IR spectrum of compound **2**.

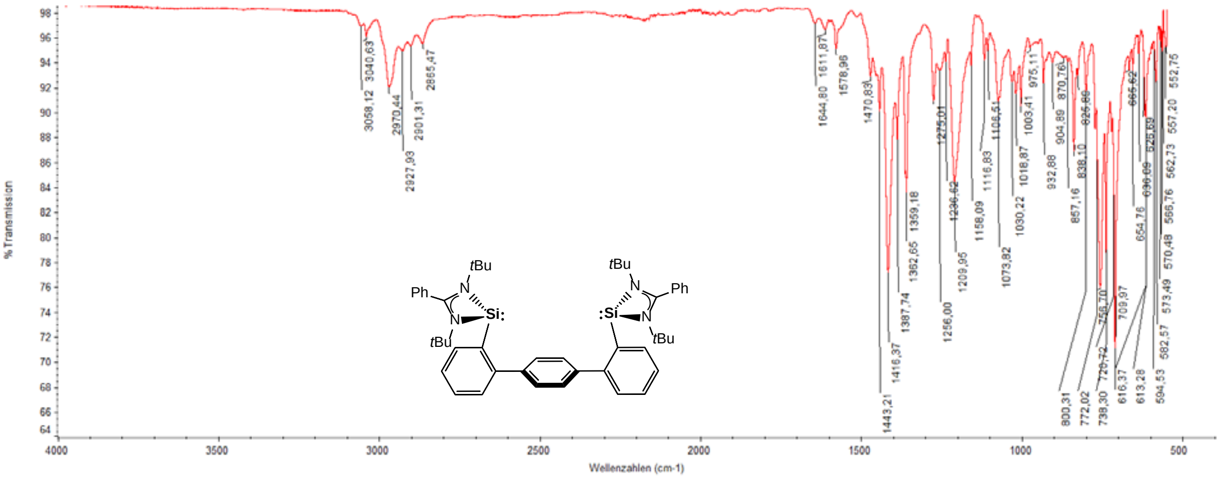

Fig. S20: FT-IR spectrum of compound **3**.

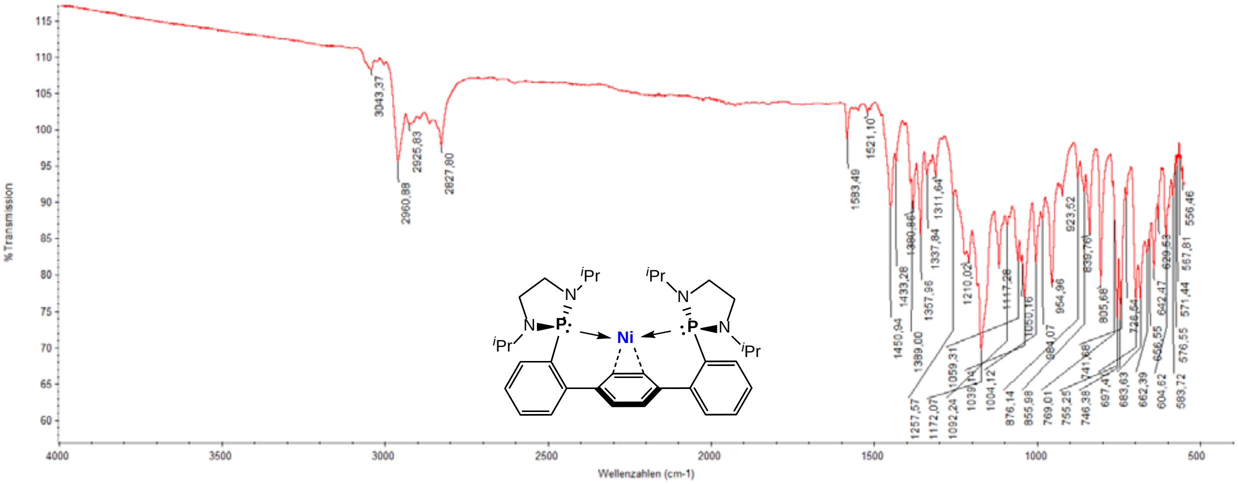

Fig. S21: FT-IR spectum of compound **4**

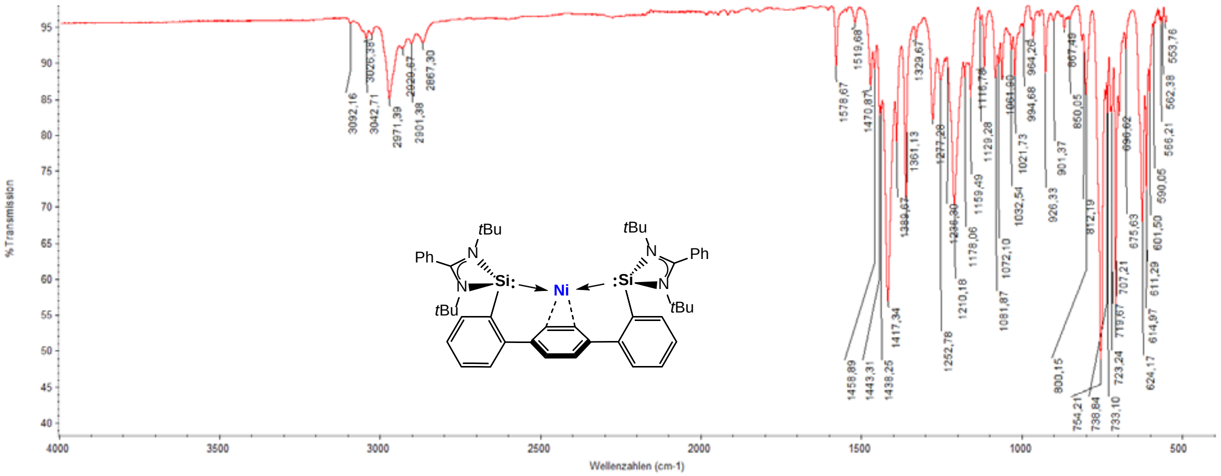

Fig. S22: FT-IR spectrum of compound **5**.

6) ESI-MS Spectra:

Compound **2**

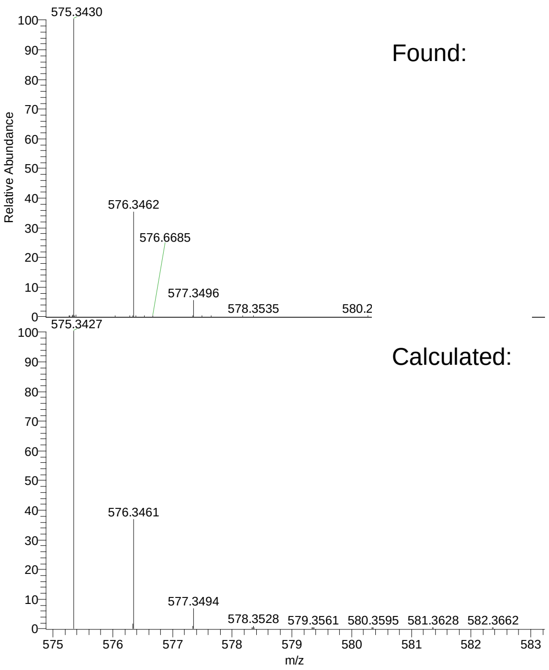

Compound **4**

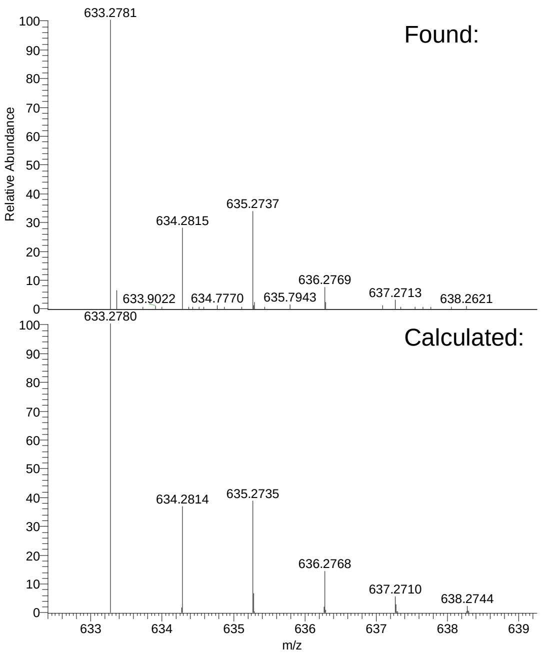

Compound **3**

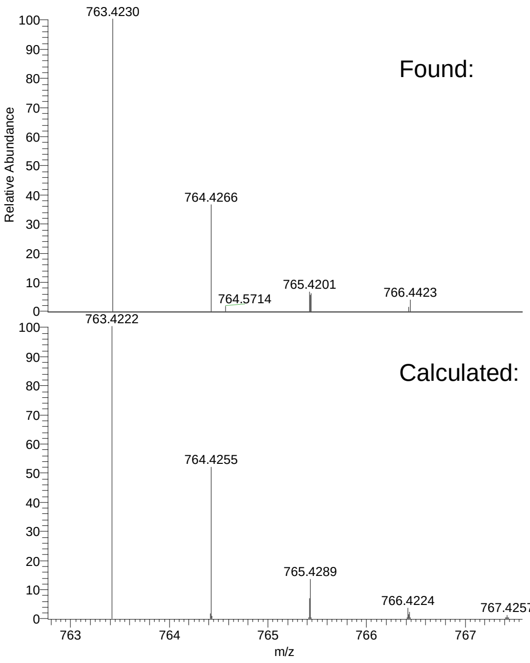

Compound **5**

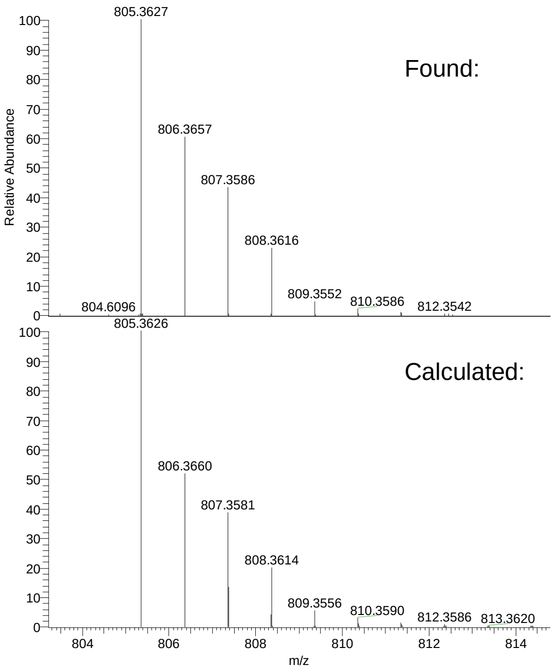

Fig. S23: ESI-MS spectra of compound **2**,**3**,**4** and **5**.

7) Cyclic Voltammetry Measurements

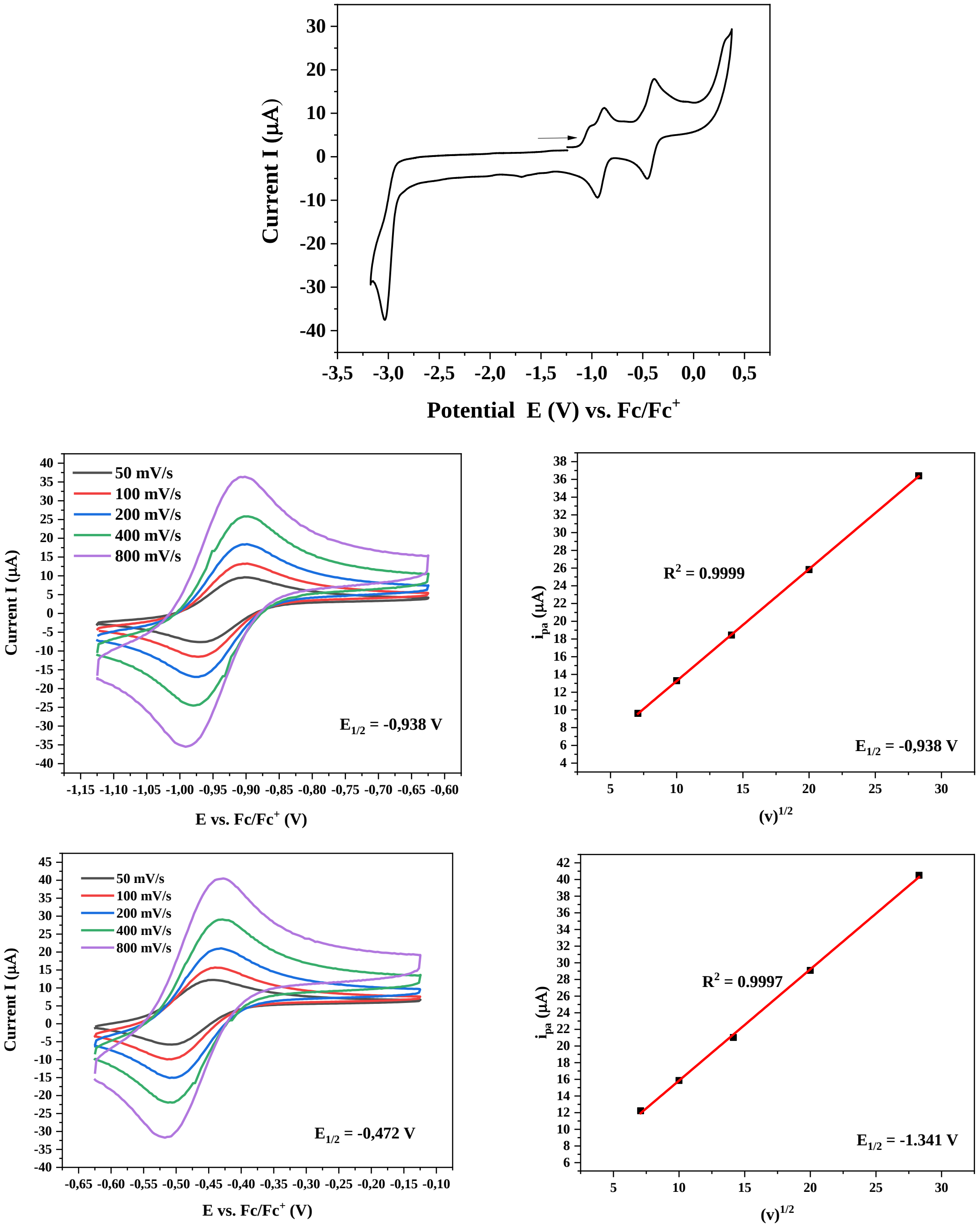

Fig. 24: Top: CV of complex **4** (1 mM in THF/ 0.3 M $TBAPF_6$) recorded at a scan rate v = 100 mV·s^{-1} showed two reversible redox events. Bottom: Reversible redox event at $E^{1/2}$ = -0.938 V and $E^{1/2}$ = - 0.472 V (vs Fc/Fc^+) measured with scan rates v = 50-800 mVs^{-1}.

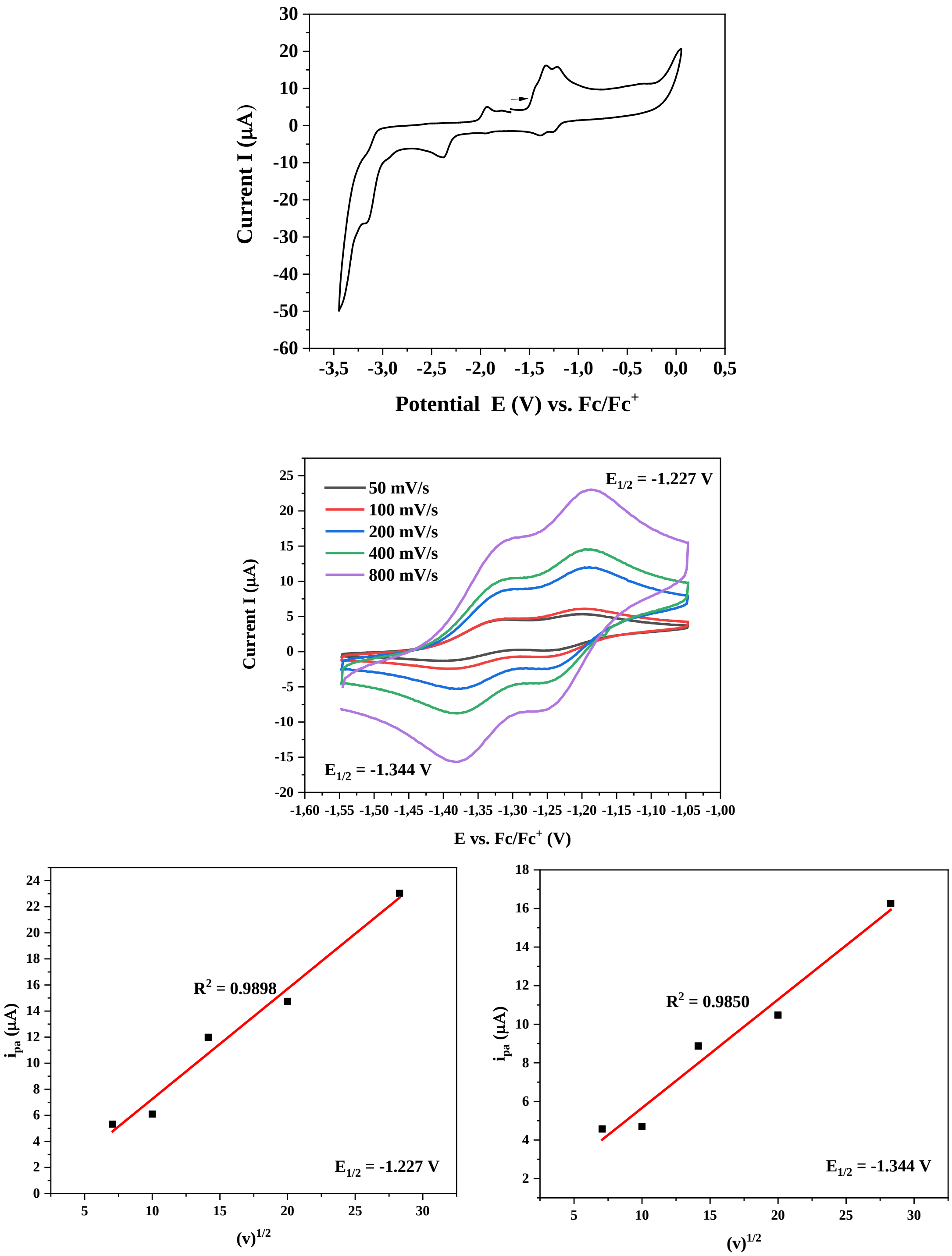

Fig. 25: Top: CV of complex **5** (1 mM in THF/ 0.3 M $TBAPF_6$) recorded at a scan rate v = 100 mV·s^{-1} showed two reversible redox events. Bottom: Reversible redox event at $E^{1/2}$ = -1.227 V and $E^{1/2}$ = - 1.344 V (vs Fc/Fc^+) measured with scan rates v = 50-800 mVs^{-1}.

8) Reversible H_2-activation by bis(silylene)Ni(η^2-arene) 5

A J. Young NMR tube was charged with 5 mg of the Ni(0) complex and 0.45 mL d_8-THF was added into the NMR tube. After three freeze-pump-thaw cycles, the solution was subjected to 1 bar H_2 gas at room temperature upon which no color change did occur. ^{1}H-proton NMR analysis revealed the partial formation of **5-H₂** with a characteristic singlet resonance at δ = - 1.50 ppm (2 H) and δ = + 5.77 ppm for the central arene protons (4 H). A mixture of initial Ni(0) complex **5** and **5-H₂** exist in an equilibrium under these conditions. Removal of H_2 by successive freeze−pump− thaw cycles regenerates **5** quantitatively. This process was repeated and identical results were obtained with no indication of decomposition (S26). The ^{29}Si-NMR shifts were assigned by a ^{1}H,^{29}Si-HMQC NMR spectrum optimized for $^1J_{Si,H}$ = 45 Hz as obtained from ^{29}Si-satelites ($^1J_{Si,H}$ = 44.6 Hz, S27).

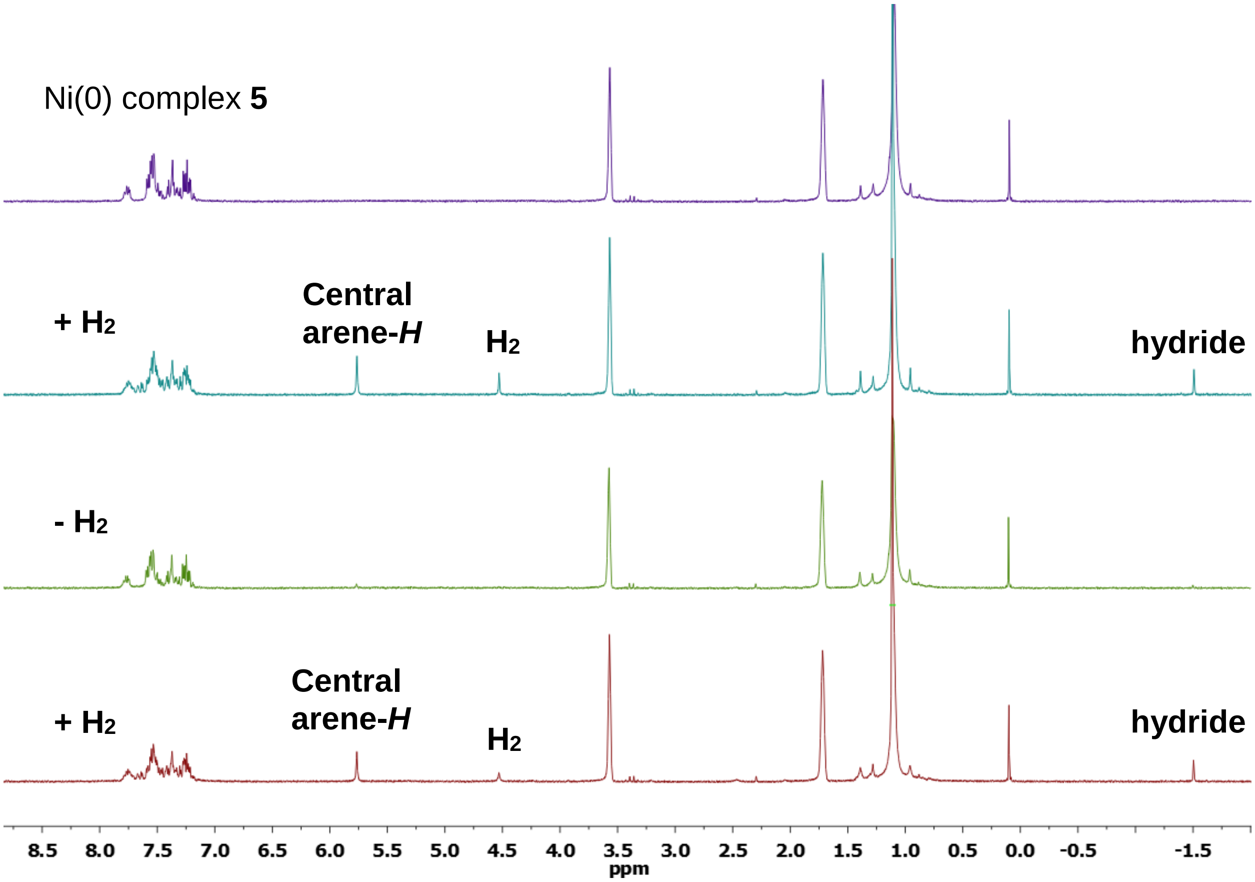

Fig. S26: ^{1}H NMR spectrum of **5** in d_8-THF under 1 bar H_2 at 298 K. Removal of H2 regenerates the initial Ni(0) complex **5** quantitatively.

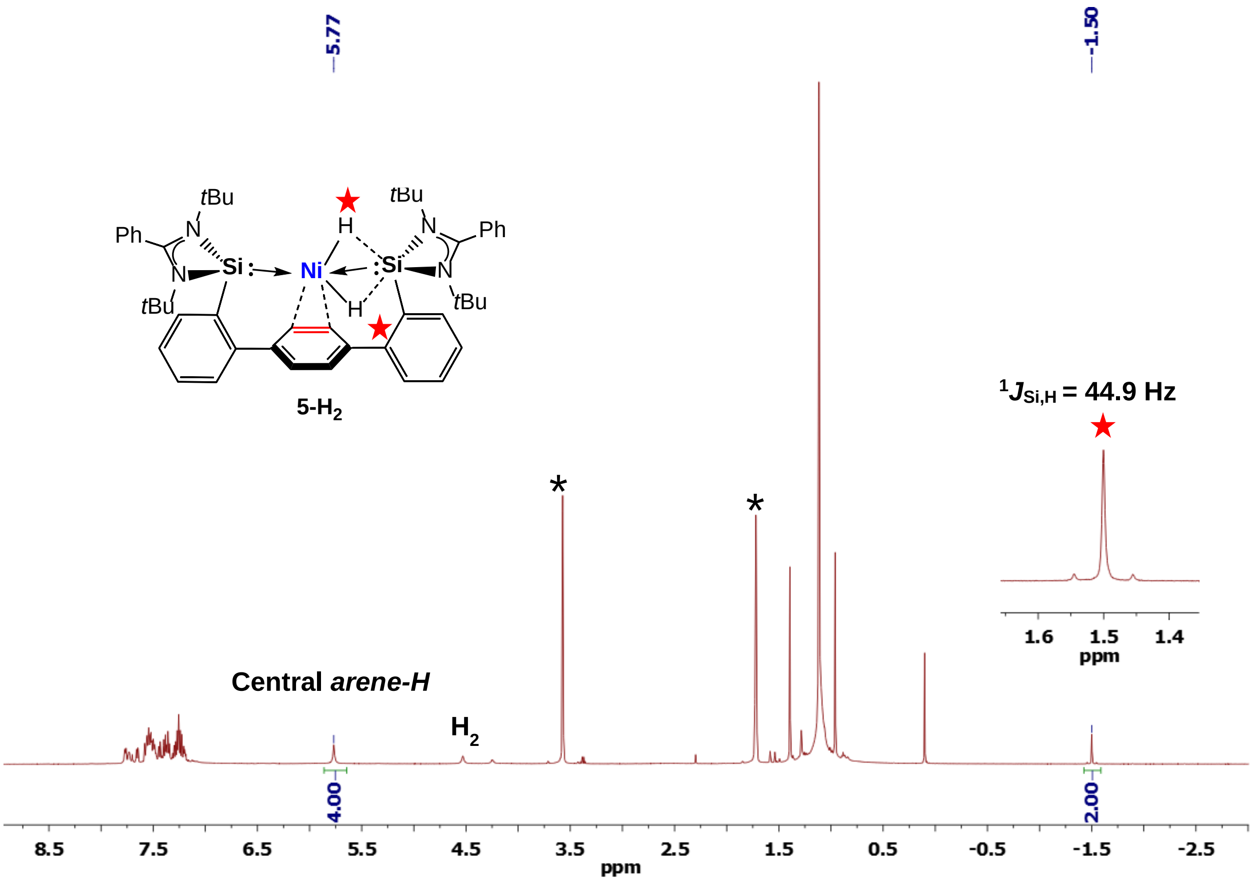

Fig. S27: ^{1}H NMR spectrum of **5** in d$_8$-THF under 1 bar H_2 at 298 K (* = solvent).

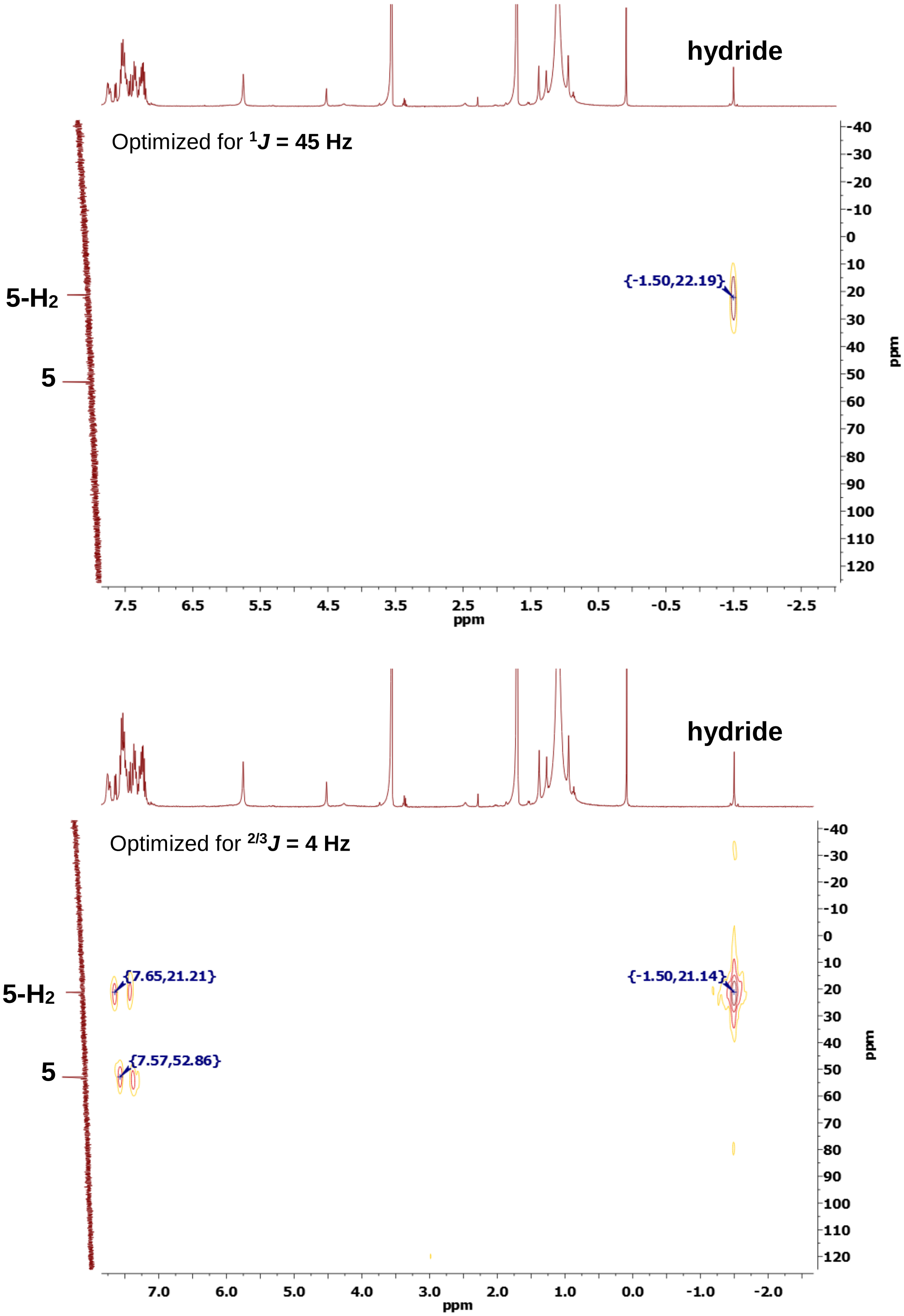

Fig. S28: Left: ^{1}H,^{29}Si-HMQC NMR spectra of **5** in d_8-THF under 1 bar H_2 at 298 K.

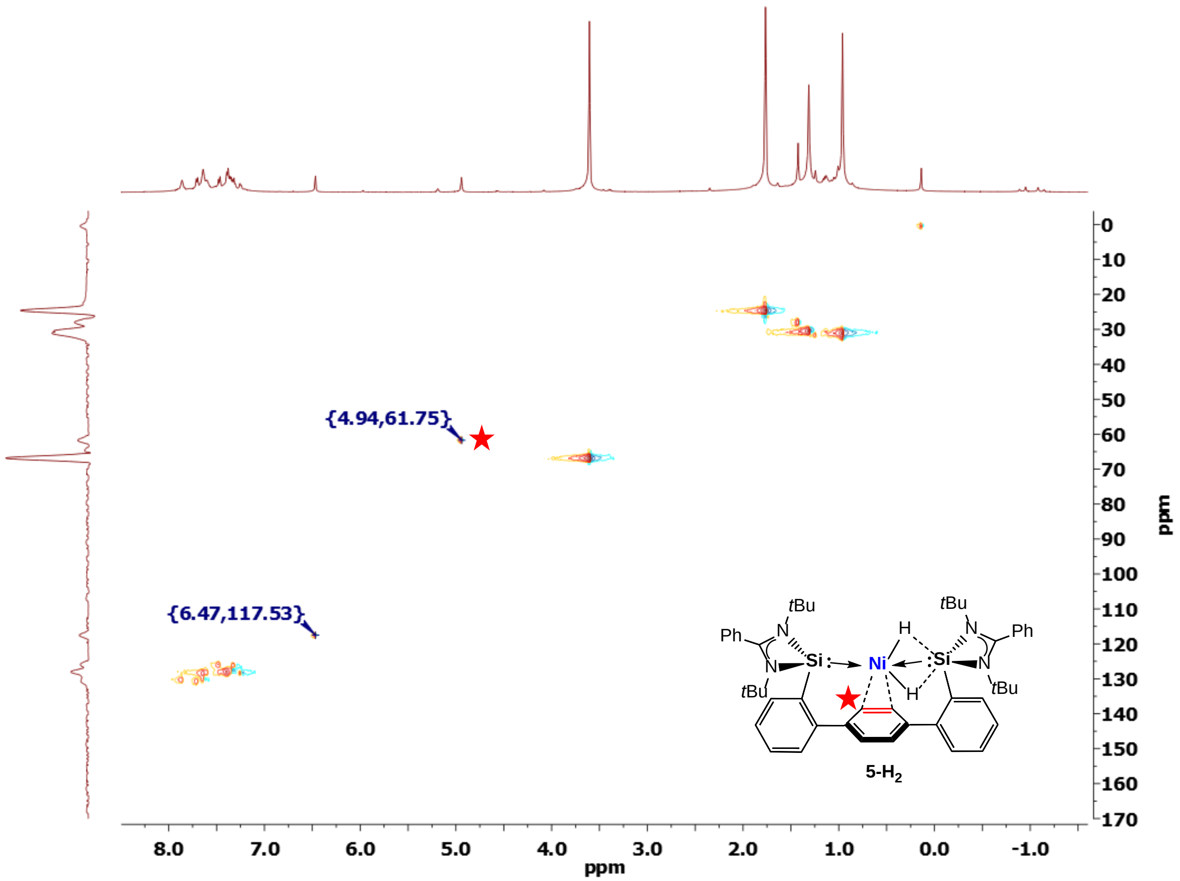

Fig. S29: $^{1}H,^{13}C$-HSQC NMR spectrum of **5** under 1 bar H_2 at 193 K.

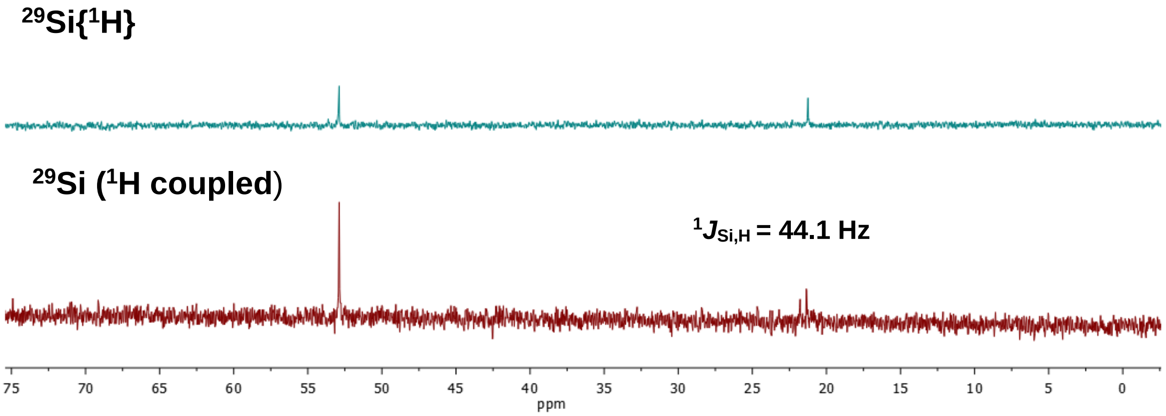

Fig. S30: ^{29}Si NMR spectra of **5** in d_8-THF under 1 bar H_2 at 298 K.

The equilibrium constant k_1/k_{-1} was determined using ferrocene as internal standard and was found to be k_1/k_{-1} = 0.66. Addition of D_2 at – 80 °C to a solution of **5/5-H$_2$** inside a J. Young NMR tube resulted in fast H-D scrambling (δ = 4.50 ppm, $^1J_{H,D}$ = 43 Hz) and the appearance of an additional triplet resonance signal at δ = - 1.24 ppm, $^2J_{H,D}$ = 4.88 Hz) corresponding to **5-HD** (S31).

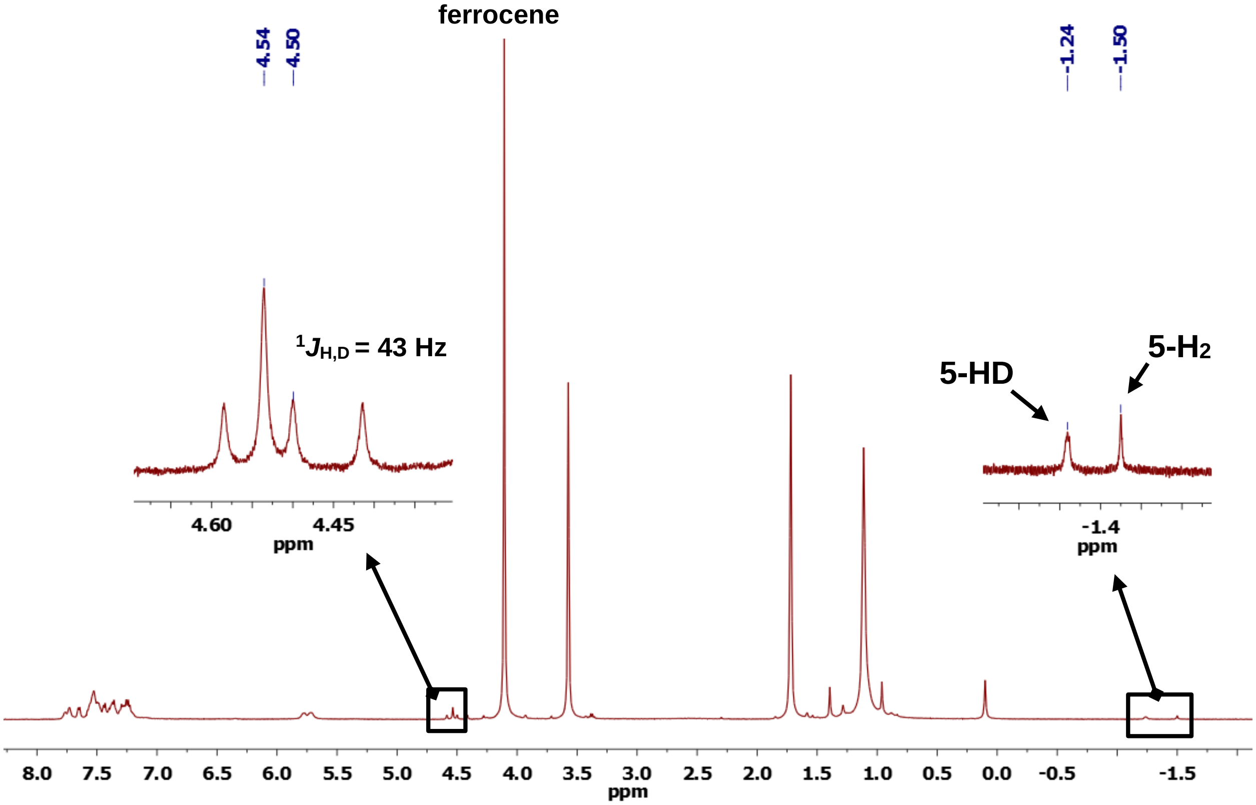

Fig. S31: ^{1}H NMR spectrum of **5** + H_2/D_2 in d_8-THF showing **5-H$_2$** and **5-HD**

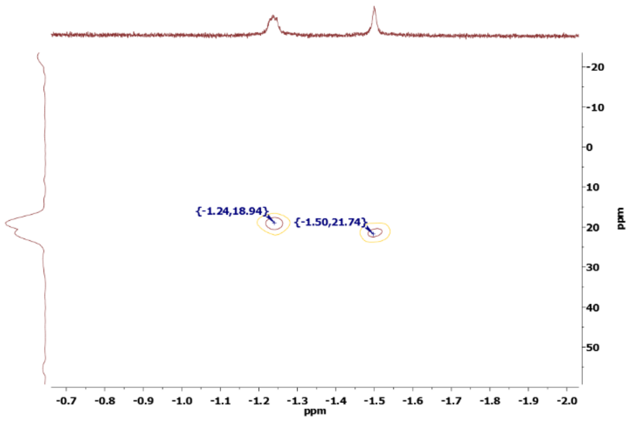

Fig. S32: ^{1}H, ^{29}Si-HMQC NMR spectrum (optimized for $J_{Si,H}$ = 45 Hz) of **5** + H_2/D_2 in d_8-THF.

H,H-EXSY NMR additionally shows the expected cross-signal for the hydride-signal and dissolved H_2 gas in d_8-THF (S33).

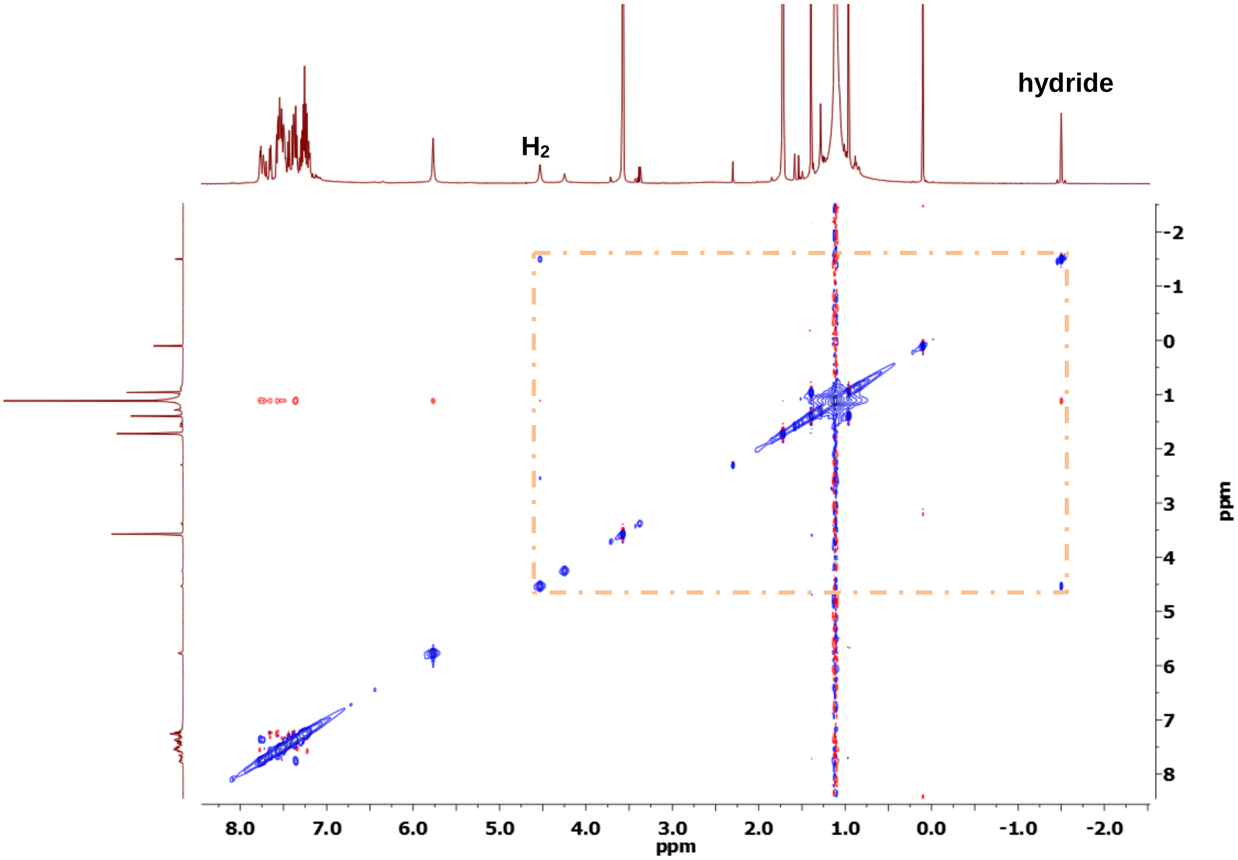

Fig. S33: H,H-EXSY NMR spectrum of **5** in d_8-THF under 1 bar H_2 at 298 K.

T_1 Time Measurement:

T_1 values were measured at 298 K and 193 K on a 500 MHz spectrometer of the corresponding hydride Signal in the ^{1}H proton NMR at δ = - 1.50 ppm in d_8-THF.

Temperature [K]	*T_1* [ms]
298	1080
193	930

Variable temperature ^{1}H NMR analysis was performed of **5** in the range of T = 298 K to T = 198 K in d_8-THF under 1 bar H_2 pressure (left) and in the presence of a mixture of H_2/D_2 (right). The temperature dependency of the hydride resonance signal at δ = -1.50 ppm is shown in Fig. 34. Coalescence was observed at T_c = 233 K. At T = 198 K two doublets resonances are obtained corresponding to two inequivalent H atoms (H^A, H^B) at low temperature which equilibrate at higher temperature.

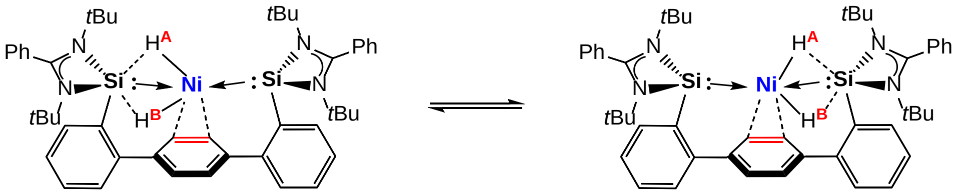

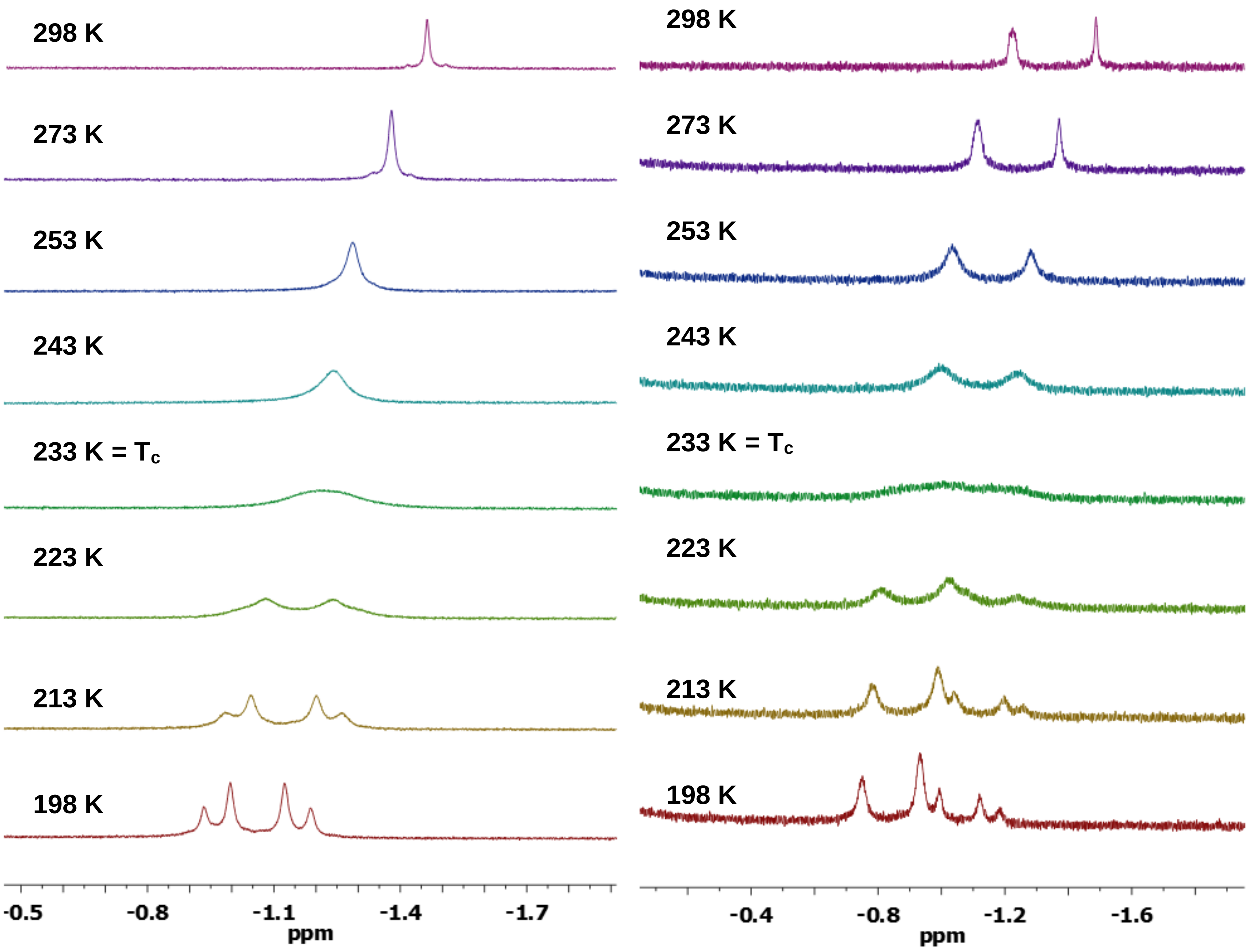

Fig. S34: VT ^{1}H NMR spectrum of **5** under 1 bar H_2 (left) and additional D_2 (right) in d_8-THF.

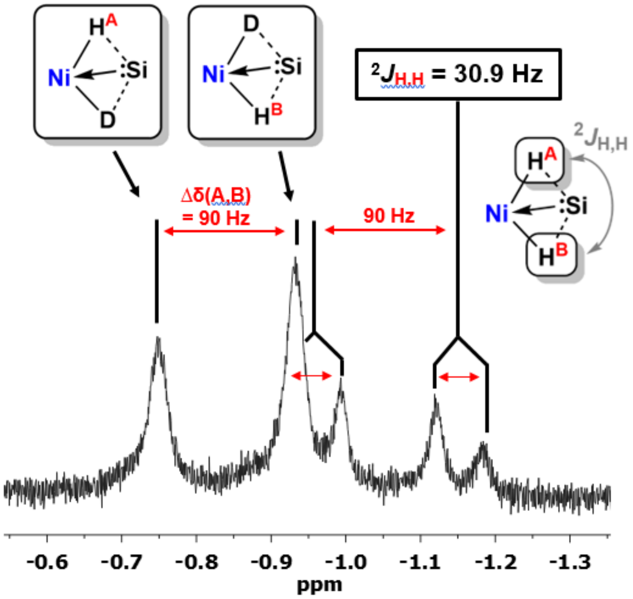

Fig. S35: ^{1}H NMR spectrum of **5** + H_2 and D_2 gas in d_8-THF at 193 K.

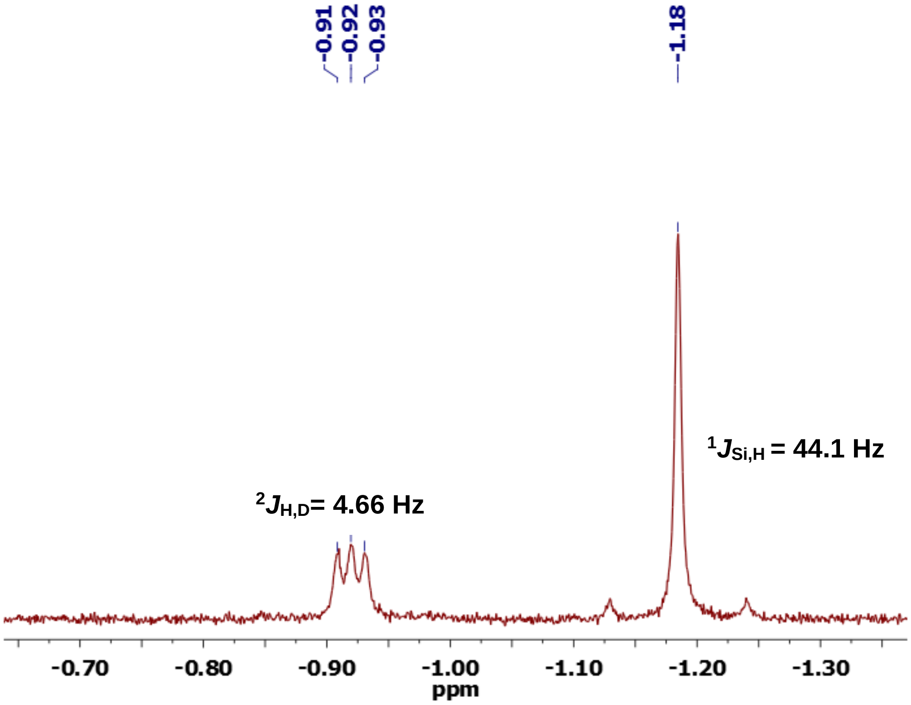

Fig. S36: ^{1}H NMR spectrum of **5** in d_7-toluene under 1 bar H_2 at 298 K.

Calculation of the free energy of activation $\Delta G^{\#}$

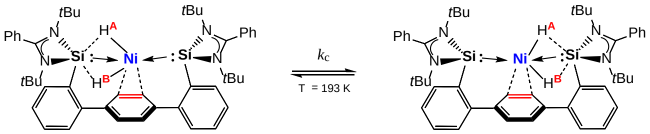

The exchange nuclei have mutual coupling ($J_{H,H}$) and the following expression was used to calculate k_{233} with Δv = 99.5 Hz and $J_{H,H}$ = 30.9 Hz from the ^{1}H NMR spectra obtained at 193 K on a 500 MHz spectrometer:

$$k_{233} = 2.22\sqrt{\Delta v^2 + 6(J_{H,H})^2}$$

$$\boldsymbol{k_{233}} = \mathbf{275\ s^{-1}}$$

The free energy of activation $\Delta G^{\#}$ was calculated based on the following expression with T_c = 233 K and k_{233} = 275 s^{-1}:

$$\Delta G^{\#} = 4.58 \cdot T_c \cdot \left(10.32 + \log\frac{T_c}{k_{233}}\right)$$

$\Delta \boldsymbol{G}^{\#}$ = **10.9 kcal/mol** (44.6 kj/mol)

9) Hydrogenation of Olefins

General Procedure for Olefin Hydrogenation. A stock solution was used containing ferrocene (0.022 mmol/0.45 mL) and catalyst **5** (1.1 µmol/0.45 mL, 2 mol%). From the stock solution, 0.45 mL were taken and transferred into a sealed J. Young NMR tube and olefin (0.054 mmol) was added. Prior to H_2 addition, a ^{1}H NMR was recorded. After three freeze-pump-thaw cycles, the solution was subjected to 1 bar H_2 at room temperature. The reaction progress was further monitored by ^{1}H NMR analysis.

Mercury Drop Test. In a 3 mL vial, norbornene (21.0 mg, 0.223 mmol), ferrocene (17 mg, 0.091 mmol) and catalyst **5** were dissolved in 2 mL C_6D_6. The red solution was transferred into a 25 mL Schlenk tube containing a stir bar. The solution was frozen in liquid nitrogen and mercury (250 mg, 1.15 mmol) was added under a N_2 atmosphere. After three freeze-pump-thaw cycles, the solution was subjected to 1 bar H_2 at room temperature and stirred for 14 h. Conversion was determined by ^{1}H NMR analysis. A full conversion (>99 %) was achieved in the presence of Hg, indicating a homogeneous reaction. However, decomposition was observed resulting in decoloration and formation of a black solid. When Ni(0) complex **5** is exposed to mercury (exc.) inside a sealed J. Young NMR tube. Slow decomposition was observed in the absence and presence of 1 bar H_2 gas in d_8-THF resulting in decoloration and the formation of a black solid. In the absence of mercury, **5** and **5-H$_2$** are stable in solution and show no decomposition within 7 days at room temperature.

Hydrogenation of 1,1-diphenylethylene. In a 3 mL vial, 1,1-diphenylacetylene (48.0 mg, 0.27 mmol) and catalyst **5** (0.5 mol%, 1.1 mg, 1.35 µmol) were dissolved in 2 mL C_6D_6. The red solution was transferred into a 25 mL Schlenk tube containing a stir bar. After three freeze-pump-thaw cycles, the solution was subjected to 1 bar H_2 at room temperature and stirred for 24 h. The mixture was filtered through silica and rinsed with additional Et_2O (1 mL). The solvent was removed affording 1,1-diphenylethane as colorless oil (43 mg, 0.24 mmol, 89 %).

Catalyst comparison in nbe hydrogenation.

Two 3 mL vials were charged with Ni(0) catalyst (1.1 µmol, 2 mol%), ferrocene (0.022 mmol) and norbornene (0.054 mmol). The mixture was dissolved in 0.45 mL C_6D_6 and transferred into a sealed J. Young NMR tube. Prior to H_2 addition, a ^1H NMR was recorded. After three freeze-pump-thaw cycles, the solution was subjected to 1 bar H_2 at room temperature. The reaction progress was further monitored by ^1H NMR analysis. Data points were fitted with a linear function ($R^2 = 0.97$).

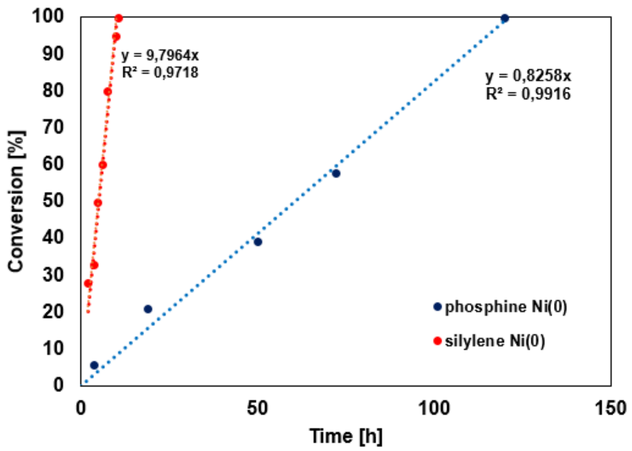

Fig. S37: Reaction profile of Ni(0) catalyzed nbe hydrogenation inside a sealed J. Young NMR tube under 1 bar H_2 at 298 K.

Bis(silylene) complex **5** catalyzes the hydrogenation of norbornene 12 times faster compared to bis(phosphine) complex **4** under identical reaction conditions. Thus, 120 h were needed to reach full conversion using 2 mol% of **4**.

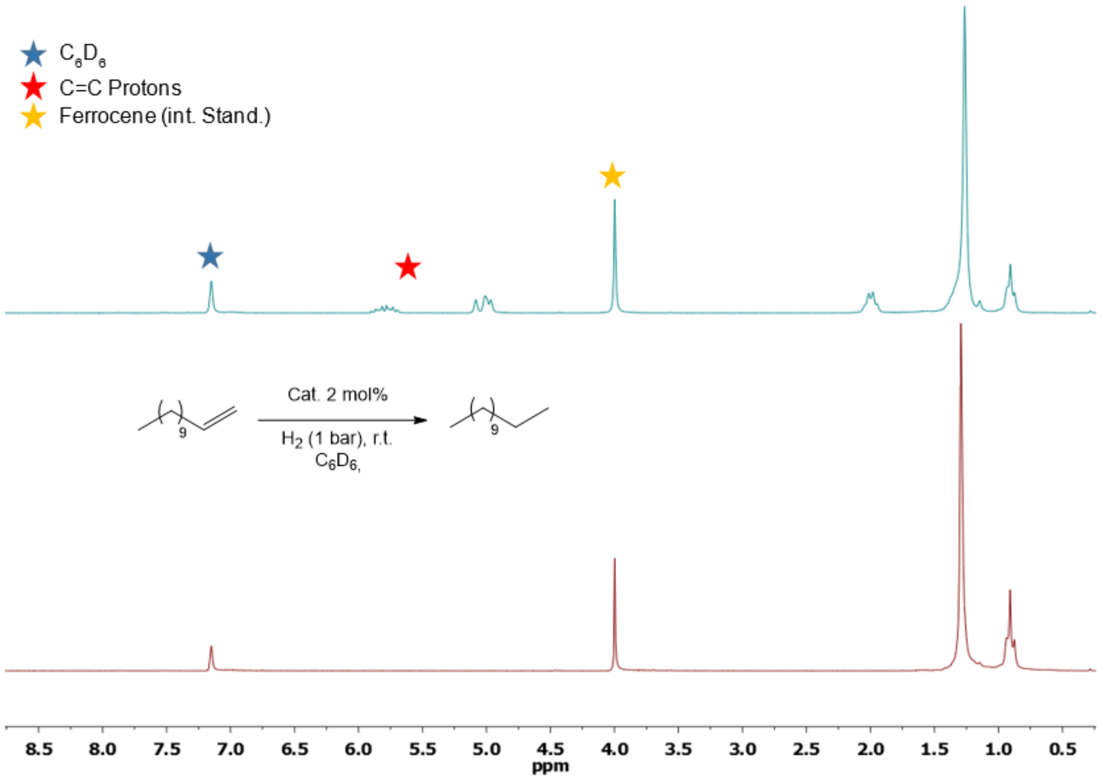

Fig. S38: ^{1}H NMR spectrum of the reaction mixture prior H_2 addition containing dodec-1-ene, ferrocene and 2 mol% catalyst **5** (top). ^{1}H NMR spectrum under 1 bar H_2 after 24 h (bottom**).**

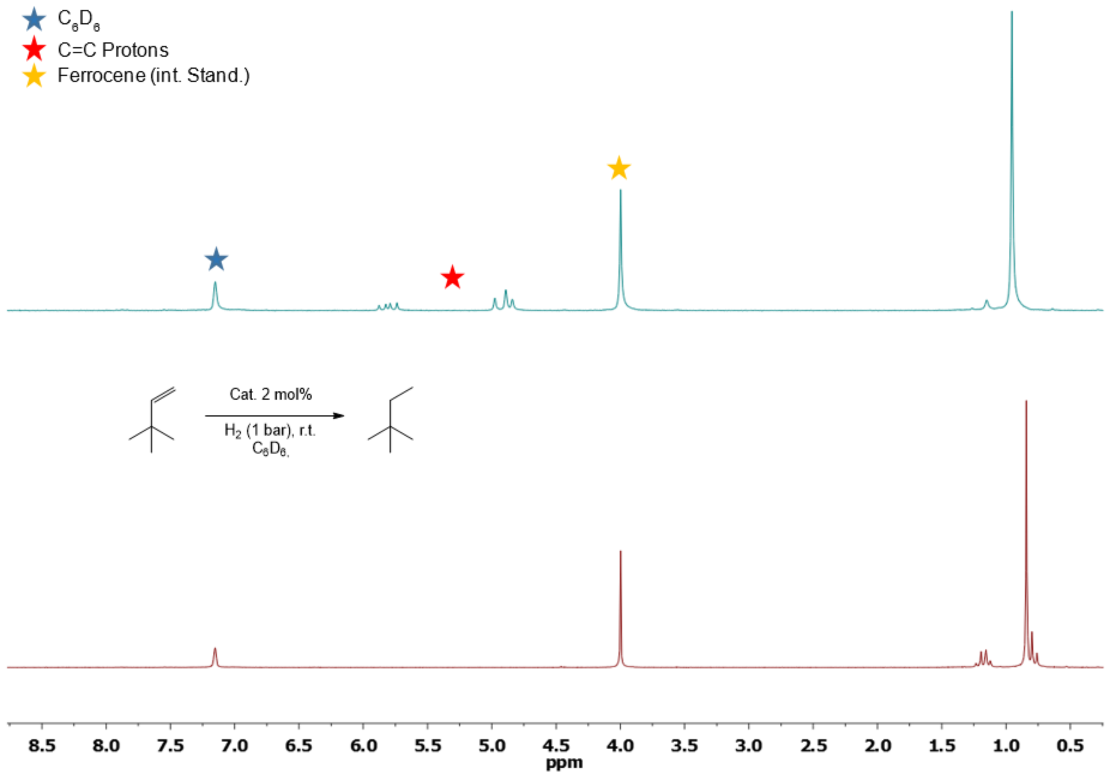

Fig. S39: ^{1}H NMR spectrum of the reaction mixture prior H_2 addition containing 3,3-dimethyl1-1butene, ferrocene and 2 mol% catalyst **5** (top). ^{1}H NMR spectrum under 1 bar H_2 after 3h (bottom**).**

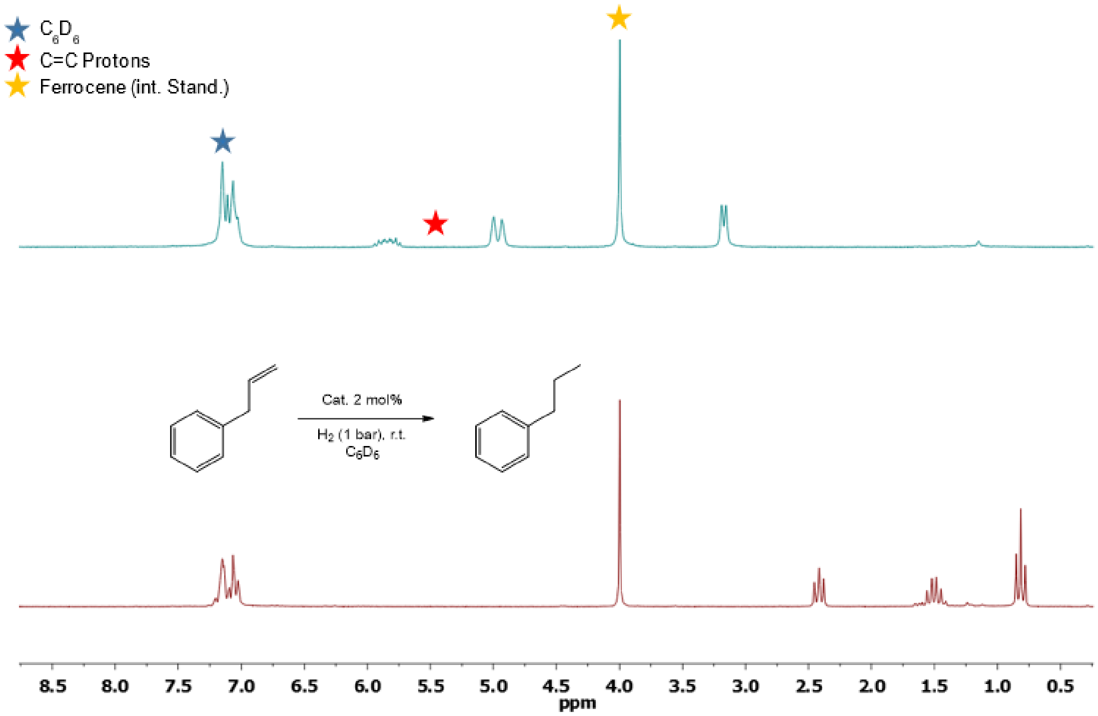

Fig. S40: ^{1}H NMR spectrum of the reaction mixture prior H_2 addition containing allylbenzene, ferrocene and 2 mol% catalyst **5** (top). ^{1}H NMR spectrum under 1 bar H_2 after 5 h (bottom).

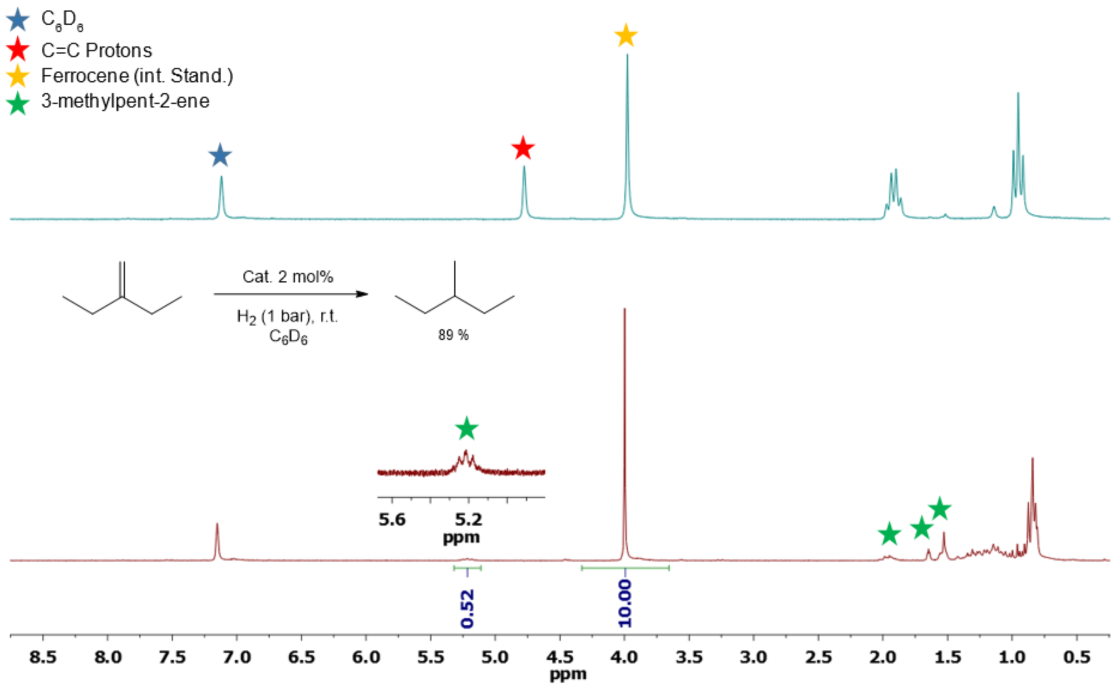

Fig. S41: ^{1}H NMR spectrum of the reaction mixture prior H_2 addition containing 3-methylenepentane, ferrocene and 2 mol% catalyst **5** (top). ^{1}H NMR spectrum under 1 bar H_2 after 18 h (bottom).

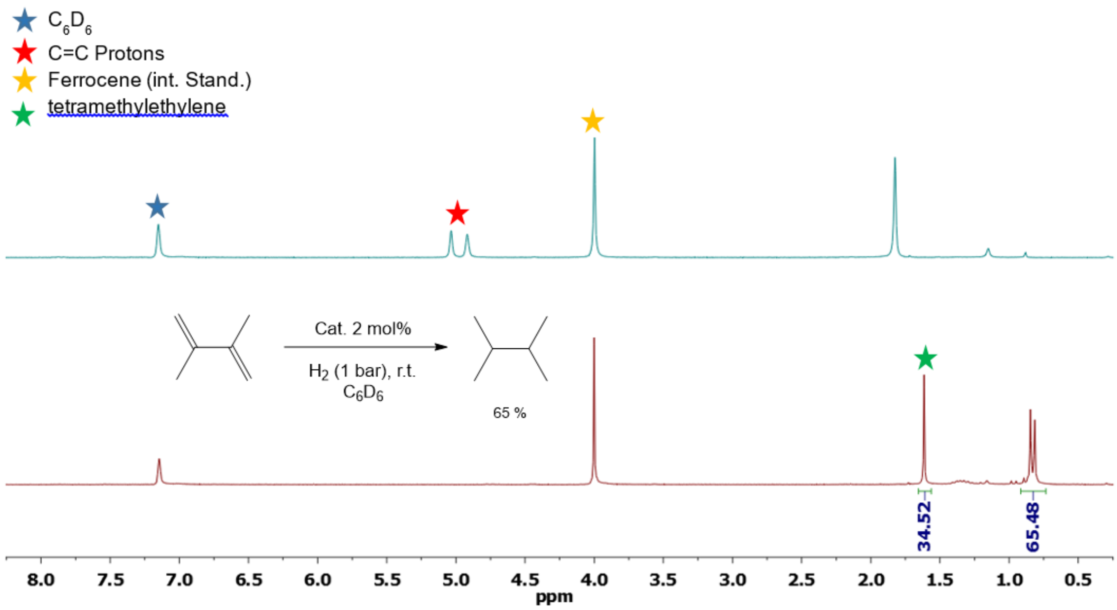

Fig. S42: ^{1}H NMR spectrum of the reaction mixture prior H_2 addition containing 2,3-dimethyl-1,3-butadiene, ferrocene and 2 mol% catalyst **5** (top). ^{1}H NMR spectrum under 1 bar H_2 after 22 h (bottom**).**

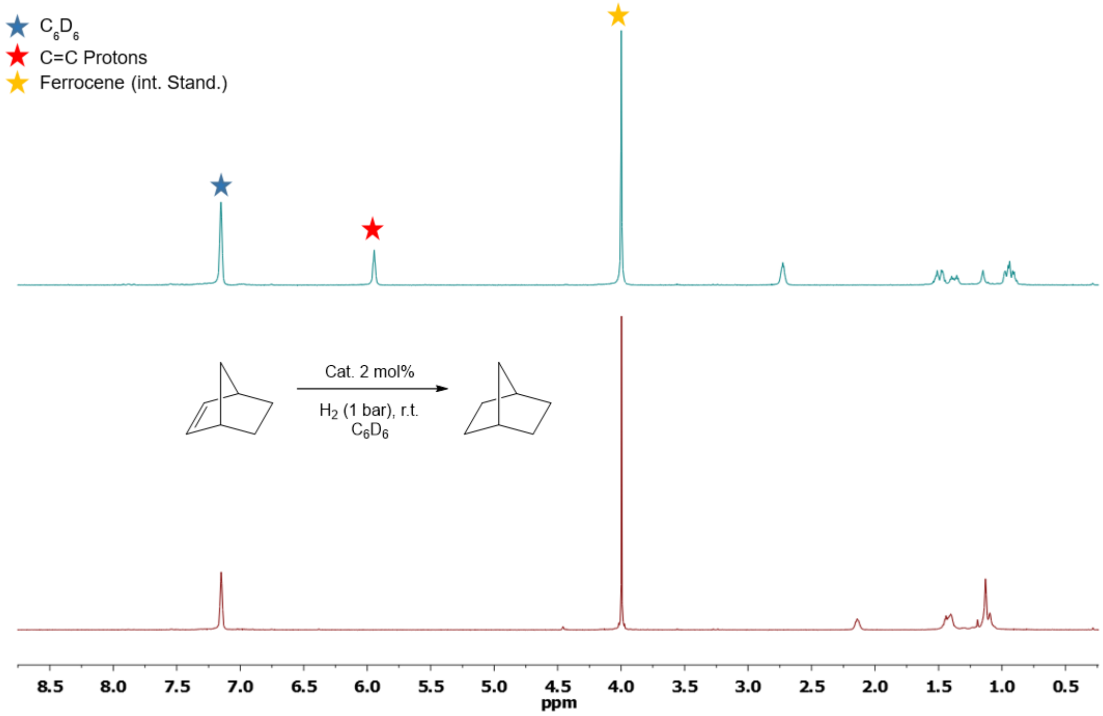

Fig. S43: ^{1}H NMR spectrum of the reaction mixture prior H_2 addition containing norbornene, ferrocene and 2 mol% catalyst **5** (top). ^{1}H NMR spectrum under 1 bar H_2 after 14 h (bottom**).**

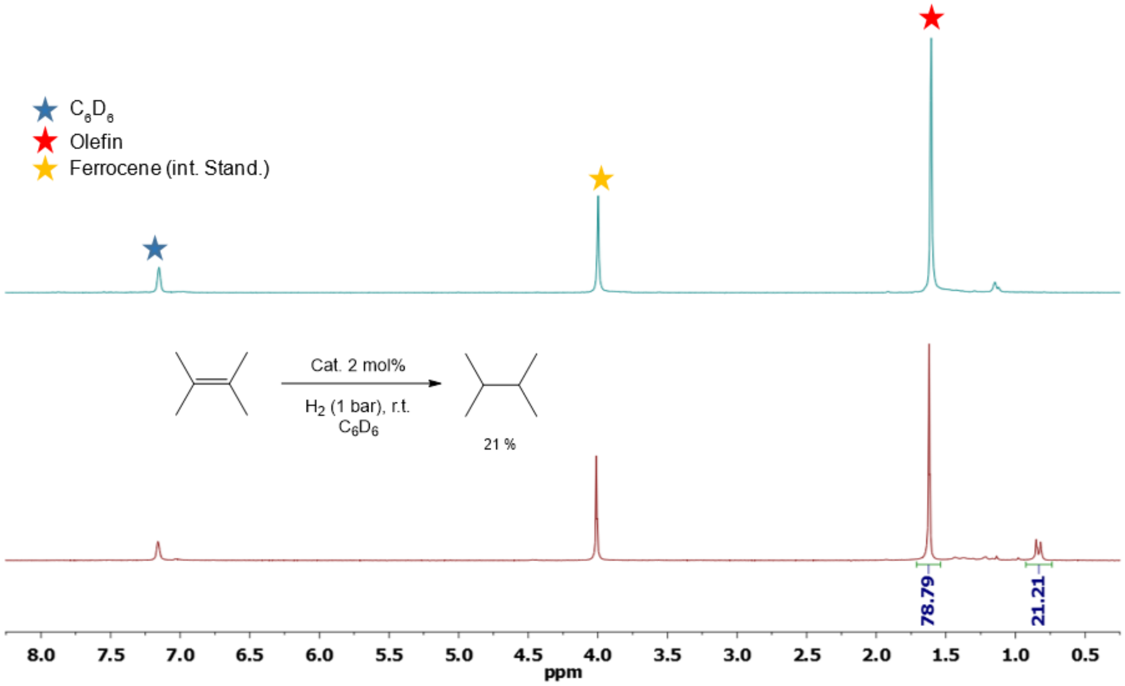

Fig. S44: ^{1}H NMR spectrum of the reaction mixture prior H_2 addition containing tetramethylethylene, ferrocene and 2 mol% catalyst **5** (top). ^{1}H NMR spectrum under 1 bar H_2 after 22 h (bottom**).**

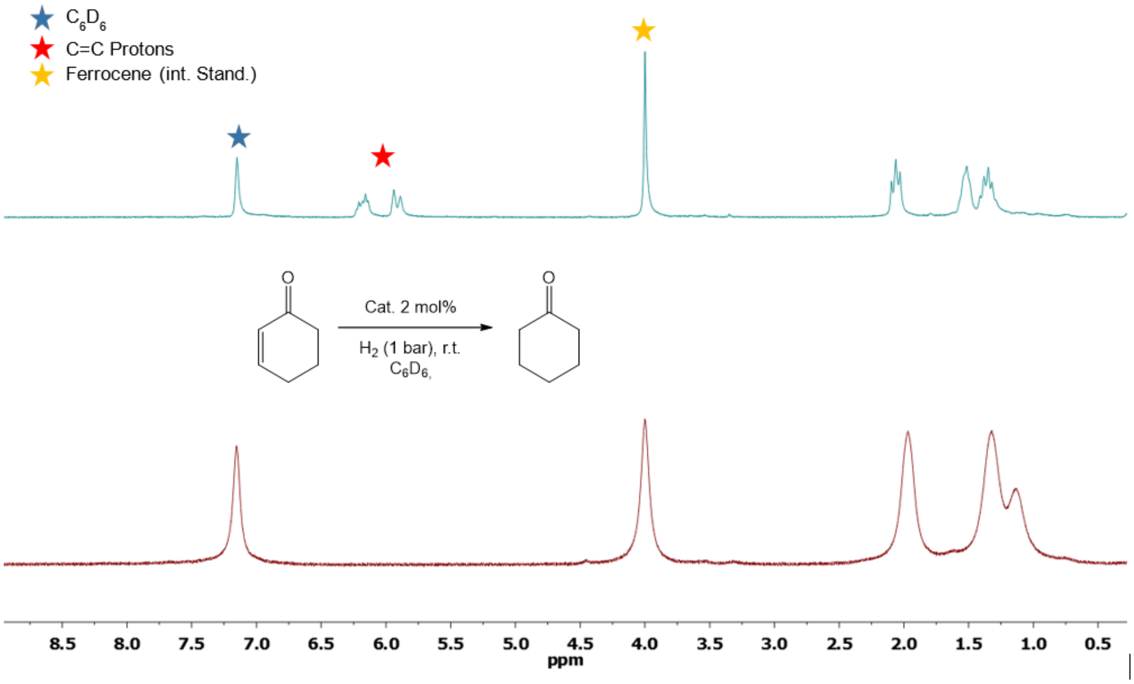

Fig. S45: ^{1}H NMR spectrum of the reaction mixture prior H_2 addition containing 2-cyclohexen-1-one, ferrocene and 2 mol% catalyst **5** (top). ^{1}H NMR spectrum under 1 bar H_2 after 12 h (bottom**).**

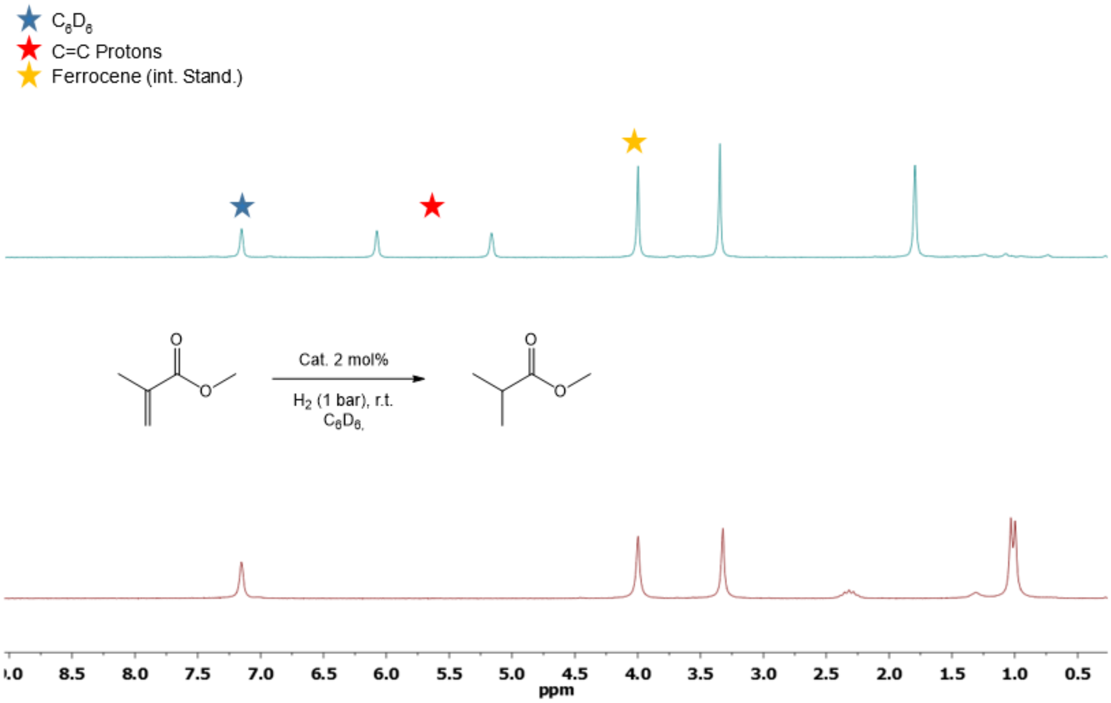

Fig. S46: ^{1}H NMR spectrum of the reaction mixture prior H_2 addition containing methyl methacrylate, ferrocene and 2 mol% catalyst **5** (top). ^{1}H NMR spectrum under 1 bar H_2 after 12 h (bottom**).**

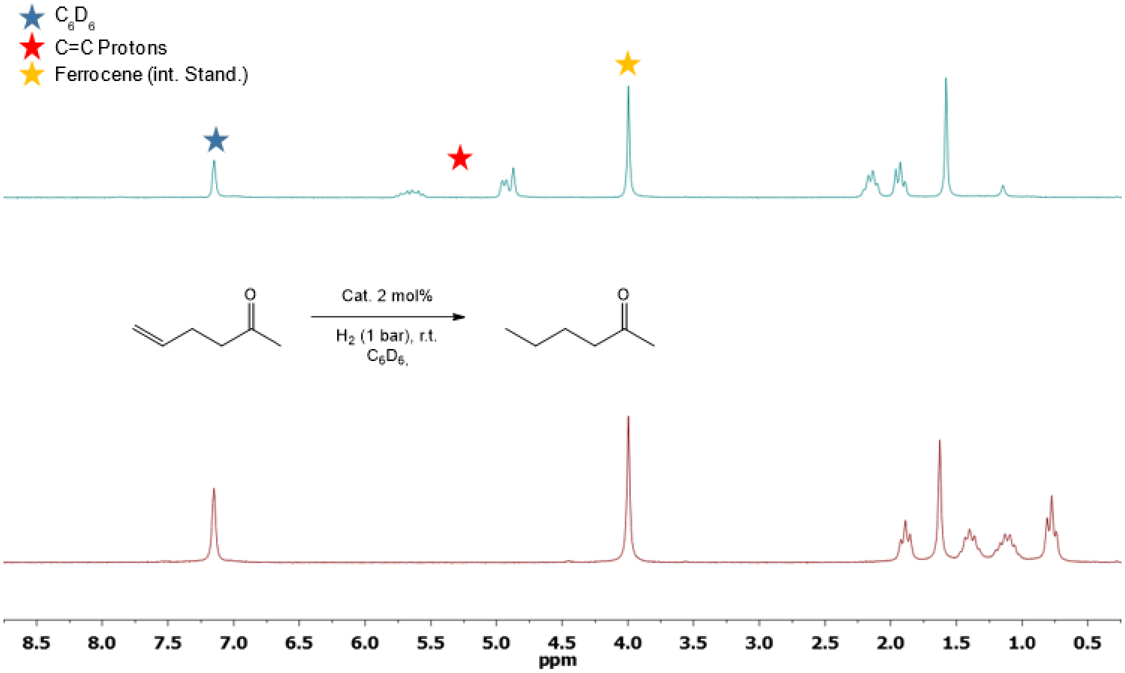

Fig. S47: ^{1}H NMR spectrum of the reaction mixture prior H_2 addition containing 5-hexen-2-one, ferrocene and 2 mol% catalyst **5** (top). ^{1}H NMR spectrum under 1 bar H_2 after 12 h (bottom**).**

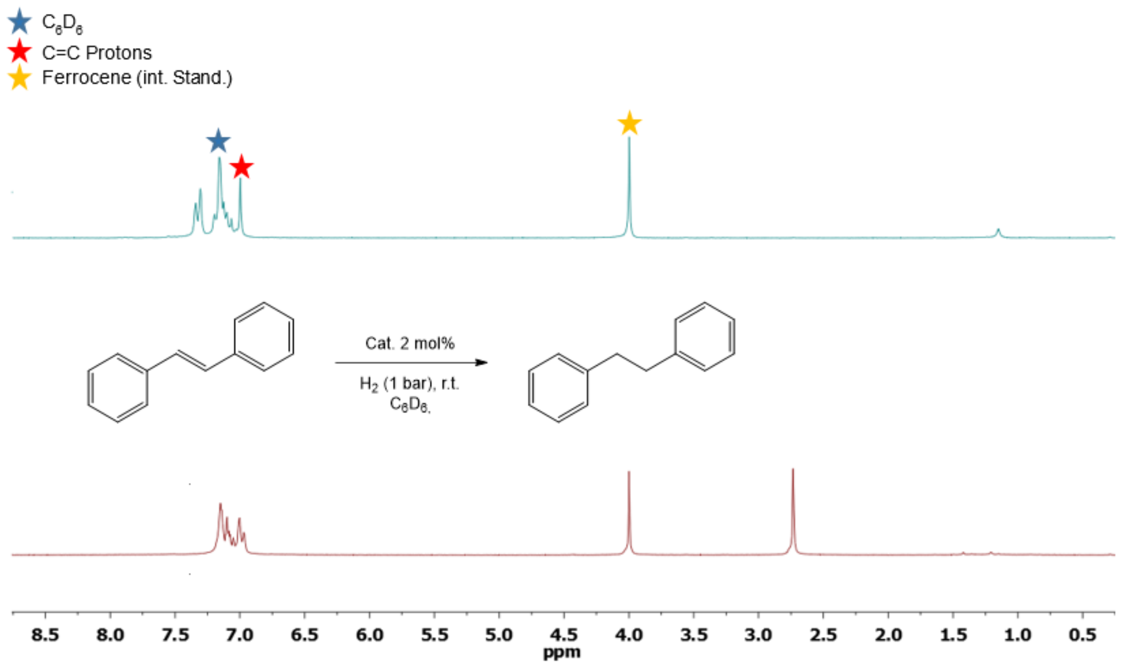

Fig. S48: ^{1}H NMR spectrum of the reaction mixture prior H_2 addition containing *trans*-stilbene, ferrocene and 2 mol% catalyst **5** (top). ^{1}H NMR spectrum under 1 bar H_2 after 72 h (bottom**).**

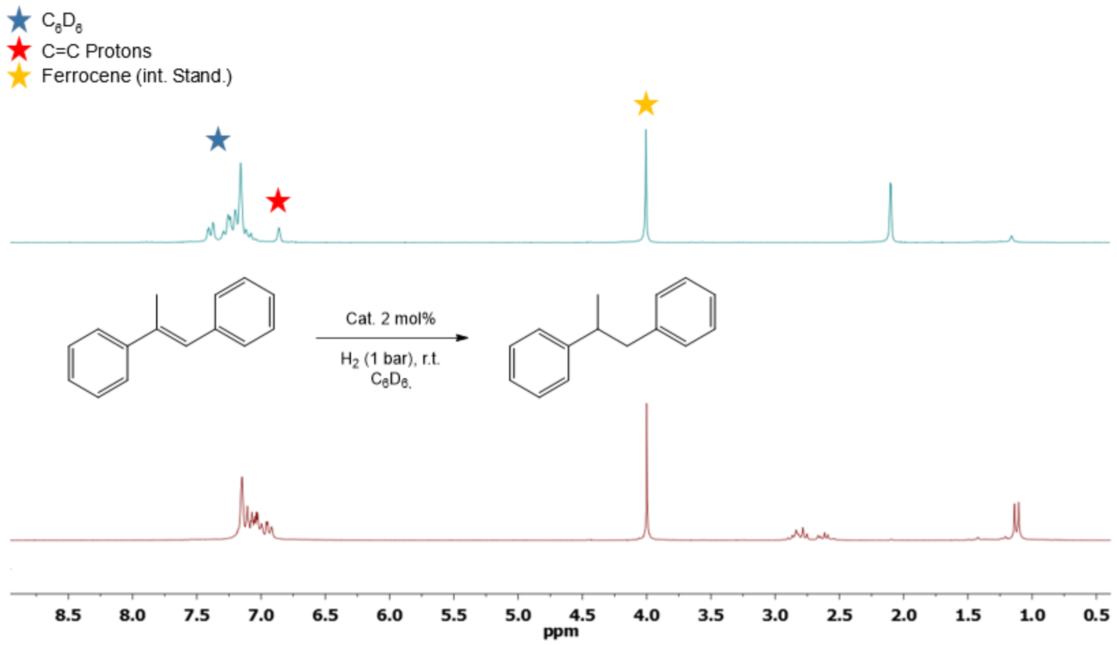

Fig. S49: ^{1}H NMR spectrum of the reaction mixture prior H_2 addition containing *β*-methylstilbene, ferrocene and 2 mol% catalyst **5** (top). ^{1}H NMR spectrum under 1 bar H_2 after 96 h (bottom**).**

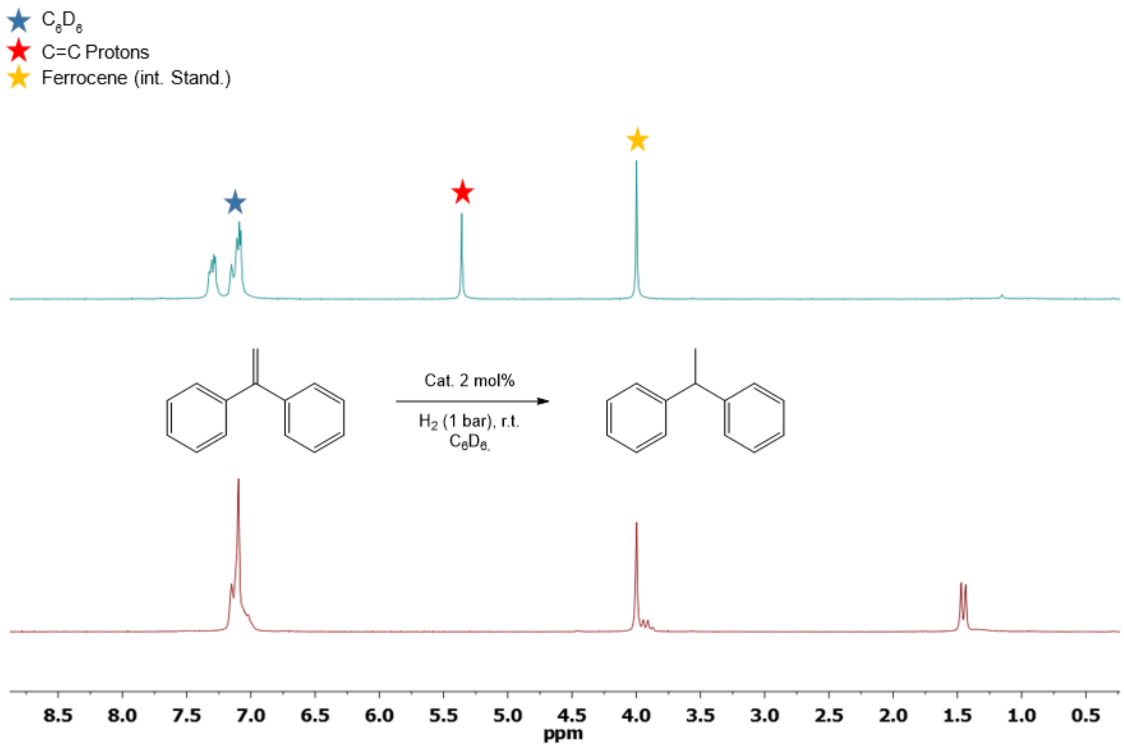

Fig. S50: ^{1}H NMR spectrum of the reaction mixture prior H_2 addition containing 1,1-diphenylethylene, ferrocene and 2 mol% catalyst **5** (top). ^{1}H NMR spectrum under 1 bar H_2 after 18 h (bottom**).**

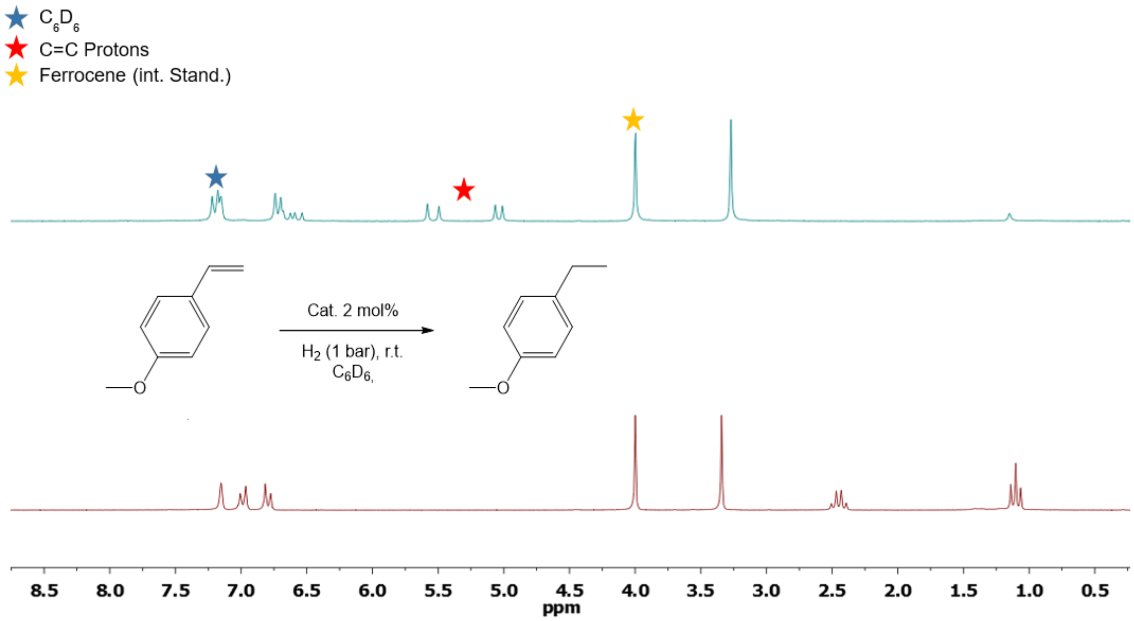

Fig. S51: ^{1}H NMR spectrum of the reaction mixture prior H_2 addition containing 4-methoxystyrene, ferrocene and 2 mol% catalyst **5** (top). ^{1}H NMR spectrum under 1 bar H_2 after 3 h (bottom**).**

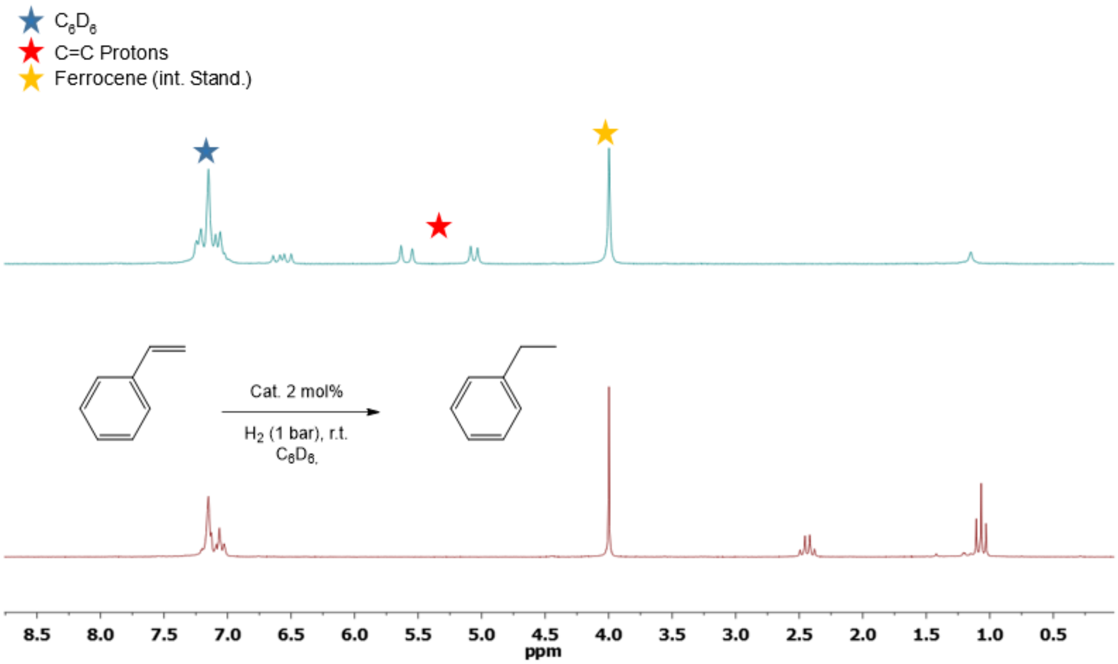

Fig. S52: ^{1}H NMR spectrum of the reaction mixture prior H_2 addition containing styrene, ferrocene and 2 mol% catalyst **5** (top). ^{1}H NMR spectrum under 1 bar H_2 after 8 h (bottom**).**

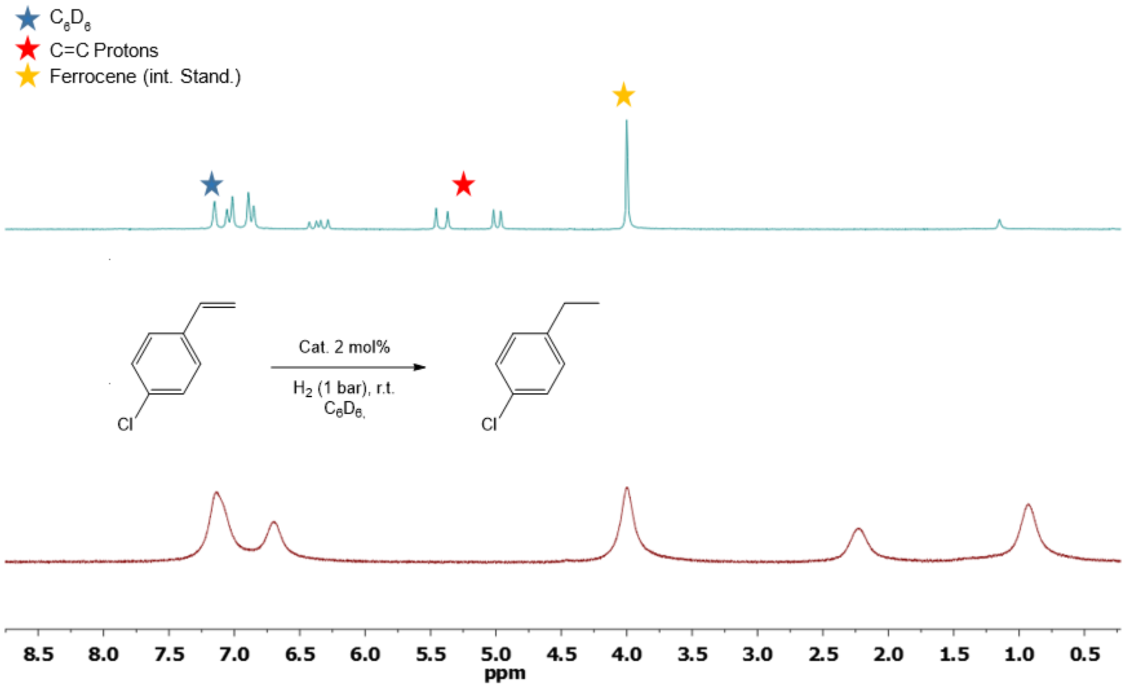

Fig. S53: ^{1}H NMR spectrum of the reaction mixture prior H_2 addition containing 4-chorostyrene, ferrocene and 2 mol% catalyst **5** (top). ^{1}H NMR spectrum under 1 bar H_2 after 14 h (bottom**).**

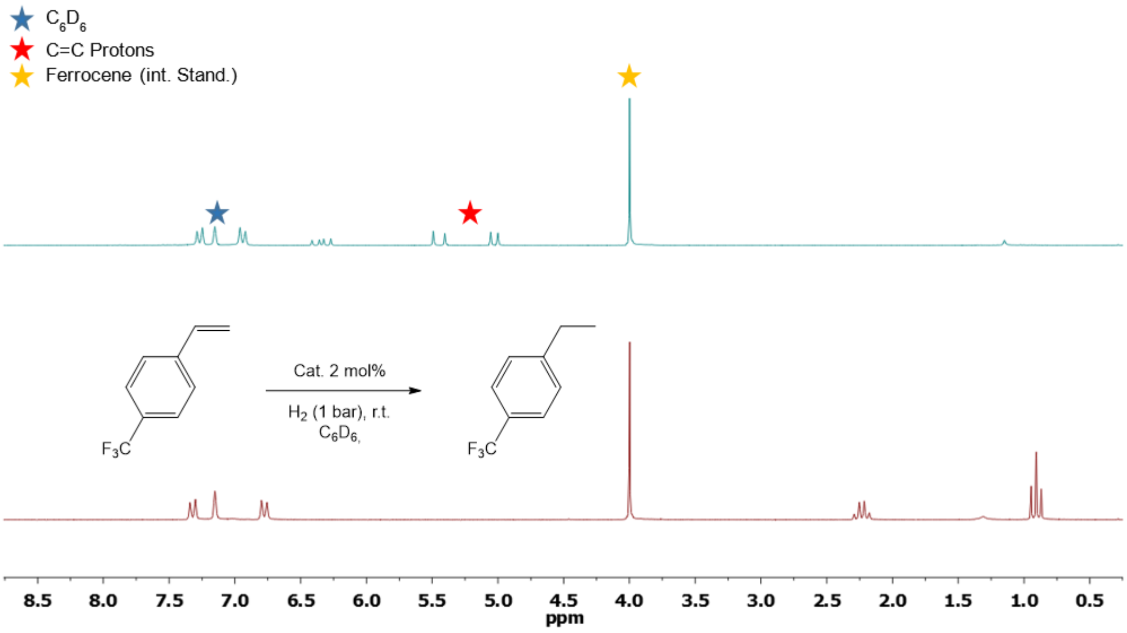

Fig. S54: ^{1}H NMR spectrum of the reaction mixture prior H_2 addition containing4-(trifluoromethyl)styrene, ferrocene and 2 mol% catalyst **5** (top). ^{1}H NMR spectrum under 1 bar H_2 after 16 h (bottom**).**

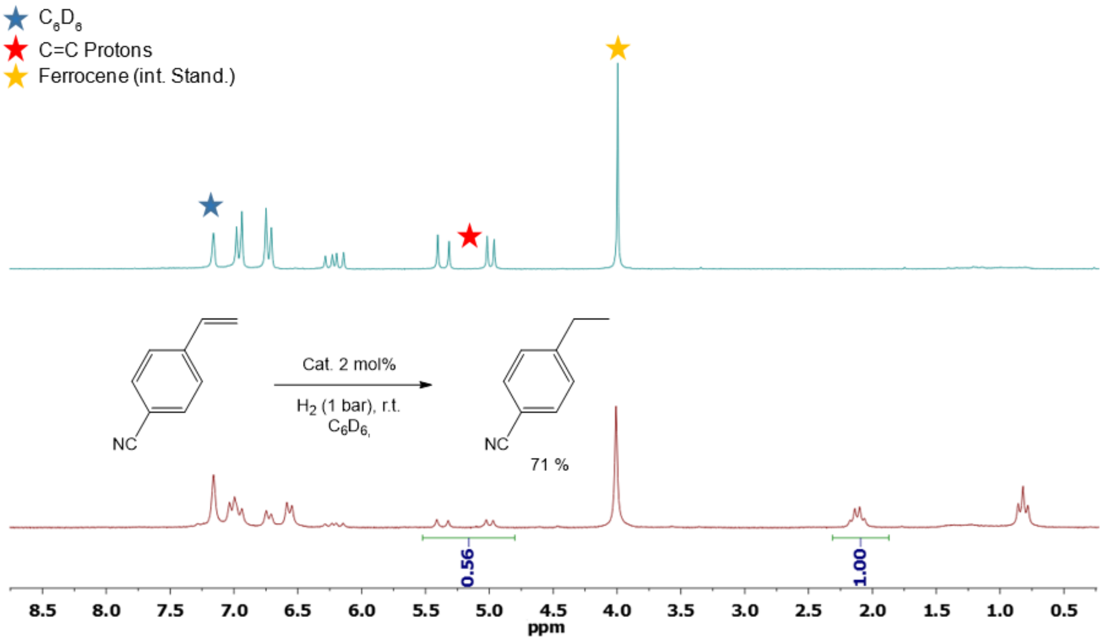

Fig. S55: ^{1}H NMR spectrum of the reaction mixture prior H_2 addition containing 4-(cyano)styrene, ferrocene and 2 mol% catalyst **5** (top). ^{1}H NMR spectrum under 1 bar H_2 after 7 h (bottom**).**

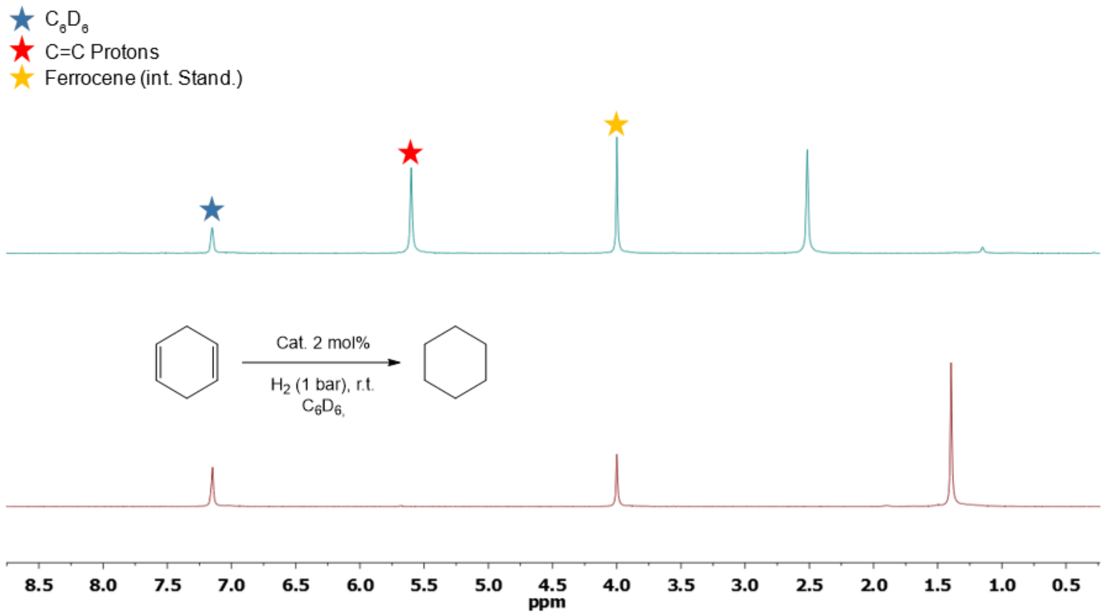

Fig. S56: ^{1}H NMR spectrum of the reaction mixture prior H_2 addition containing 1,4-cyclohexadiene, ferrocene and 2 mol% catalyst **5** (top). ^{1}H NMR spectrum under 1 bar H_2 after 28 h (bottom).

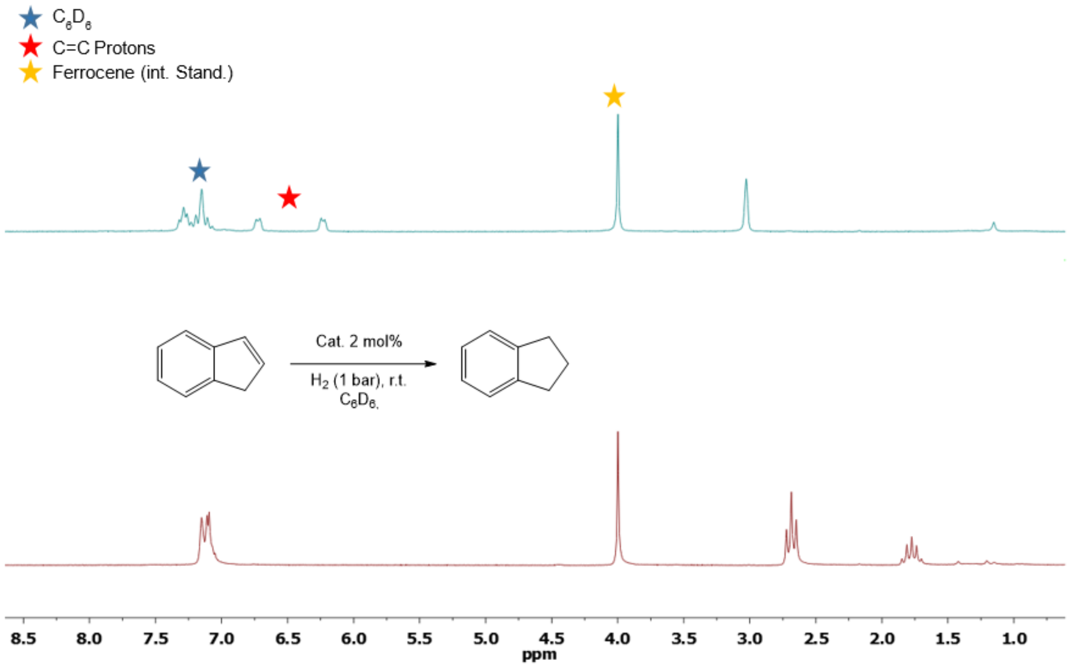

Fig. S57: ^{1}H NMR spectrum of the reaction mixture prior H_2 addition containing 1*H*-Indene, ferrocene and 2 mol% catalyst **5** (top). ^{1}H NMR spectrum under 1 bar H_2 after 42 h (bottom).

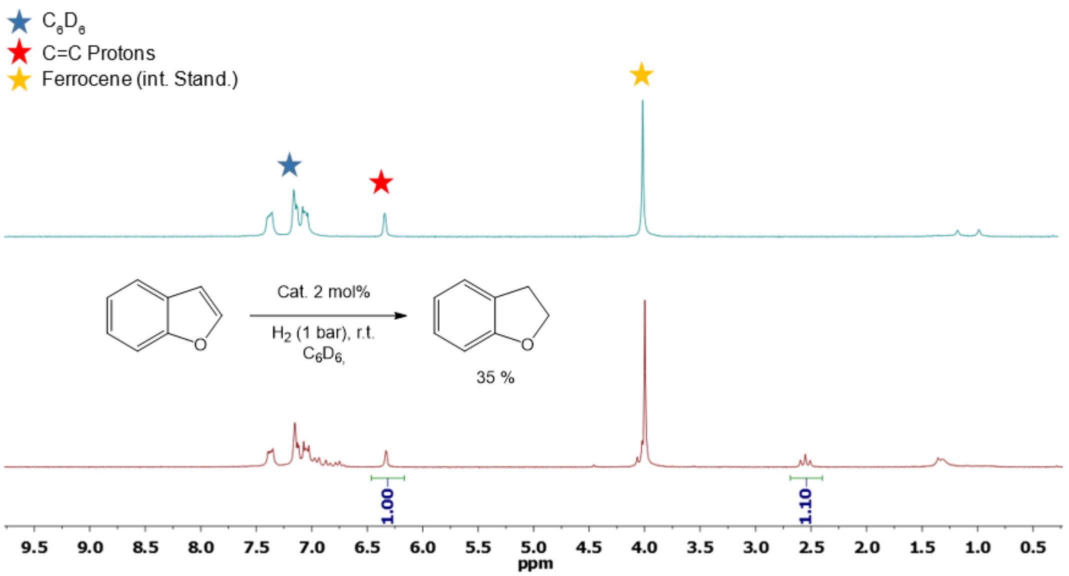

Fig. S58: ^{1}H NMR spectrum of the reaction mixture prior H_2 addition containing benzofurane, ferrocene and 2 mol% catalyst **5** (top). ^{1}H NMR spectrum under 1 bar H_2 after 72 h (botto

10) Mechanistic study / kinetic analysis:

Sub-stoichiometric experiments using 5 and norbornene.

A J. Young NMR tube was charged with 5.6 mg of the Ni(0) complex, 3.3 mg of norbornene (Nbe) and 0.45 mL d_8-THF was added into the NMR tube resulting in a homogeneous dark red solution. ^{1}H-proton NMR analysis revealed the partial formation of **5-nbe** with a characteristic pattern in the hydride region at δ = - 0.32 ppm and δ = - 0.52 ppm similar to those of literature known Ni(nbe) adducts. [S2]

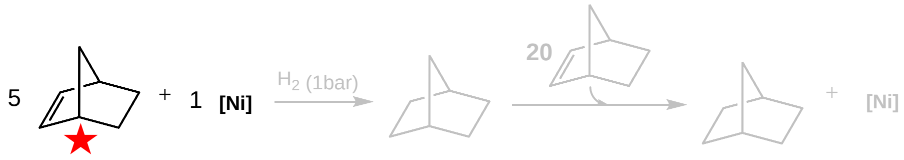

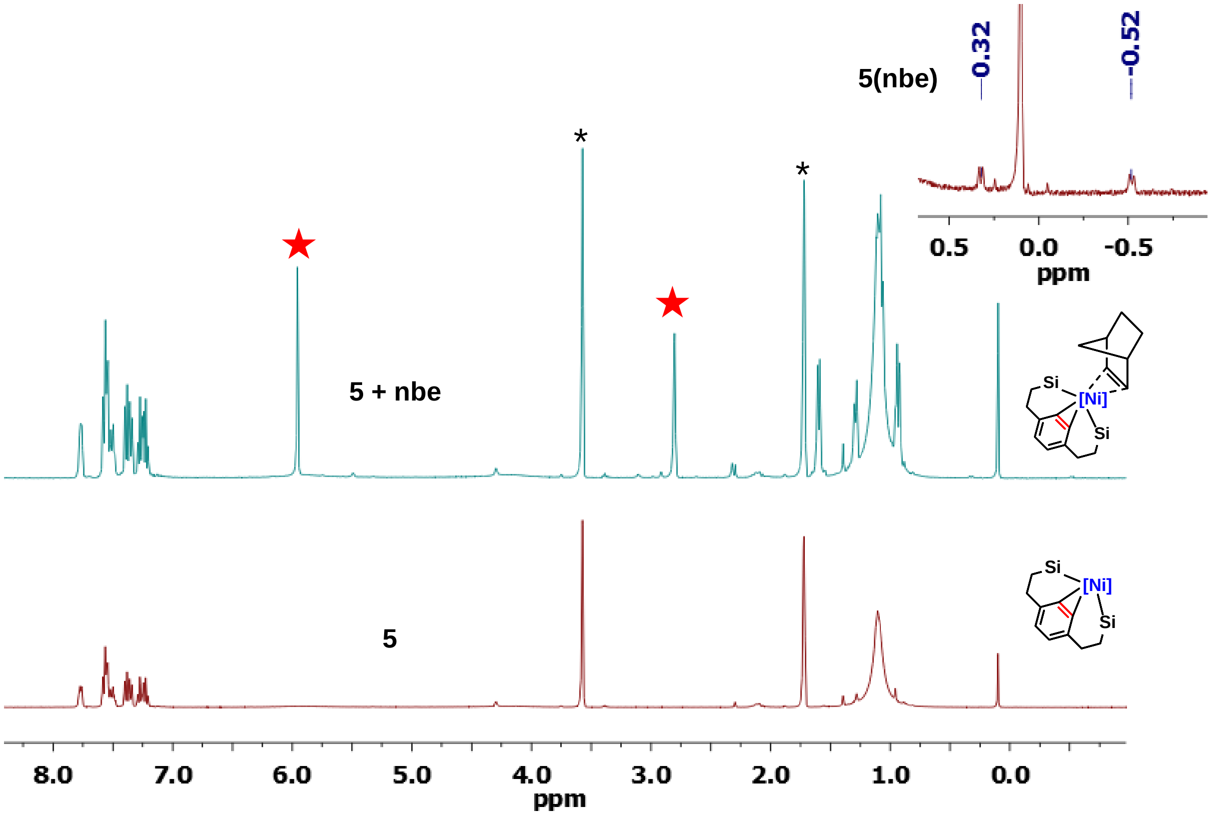

Fig. S59: ^{1}H NMR spectrum of **5** + 5 equiv. norbornene in d_8-THF at 298 K (* = solvent).

After three freeze-pump-thaw cycles, the solution was subjected to 1 bar H_2 gas at room temperature upon which no color change did occur. ^{1}H-proton NMR analysis revealed the partial formation of **5-H₂**. Hydrogenation reaction under these conditions is very slow and 2 hours were needed to reach full conversion. Integration of the resonances indicates a ratio of **5(nbe)** (σ = - 0.32/-0.52 ppm, 2H) and **5(H₂)** (2H) of 3:1 (S63) suggesting **5-nbe** as catalytic active resting state. The hydride-shift was obtained at δ = - 1.49 ppm (**5-H₂**) exhibiting sharp ^{29}Si satellites ($^1J_{Si,H}$ = 44.6 Hz) after full conversion.

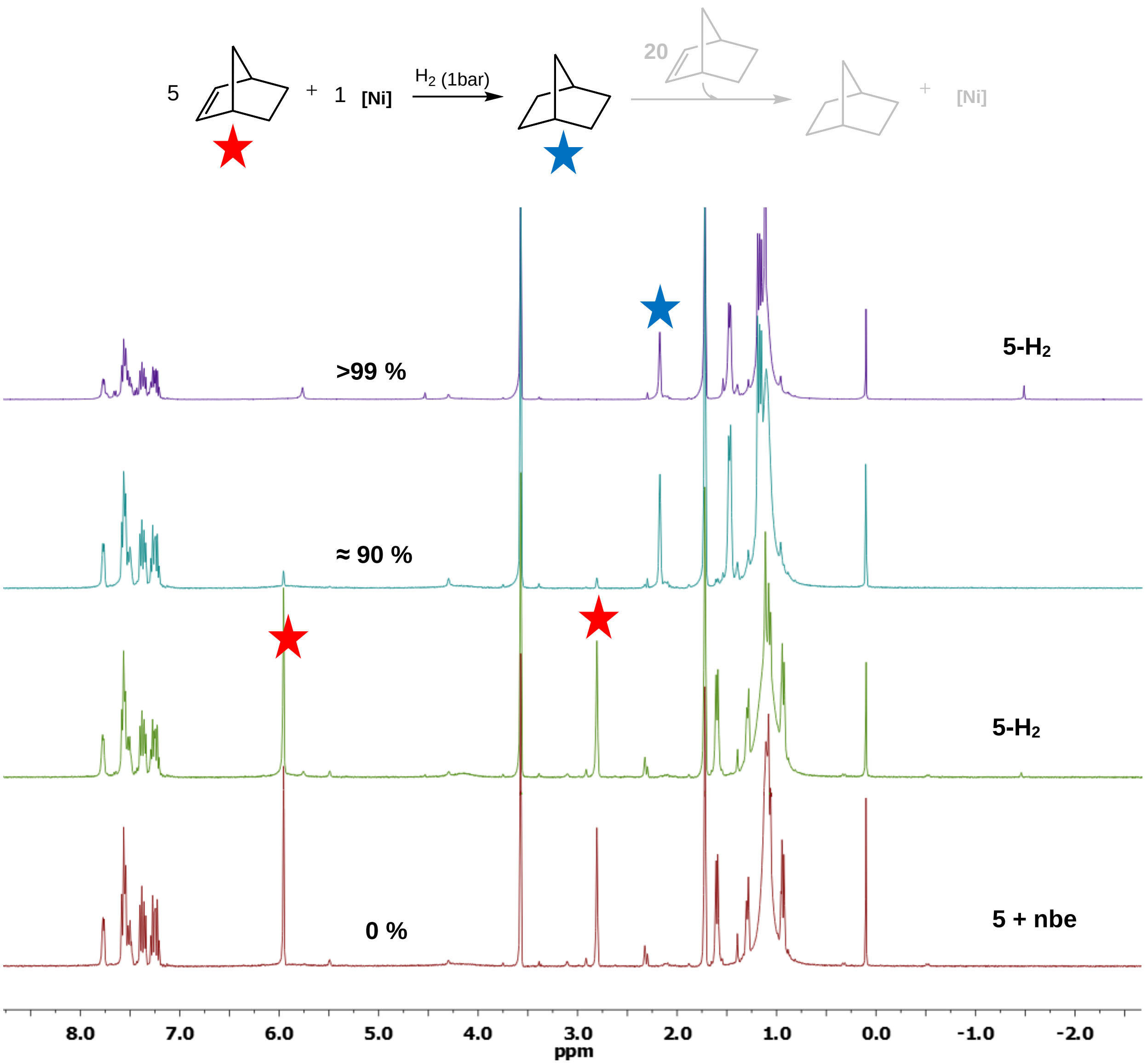

Fig. S60: ^{1}H NMR spectrum of **5** + 5 equiv. norbornene in d_8-THF at 298 K under 1 bar H_2 pressure.

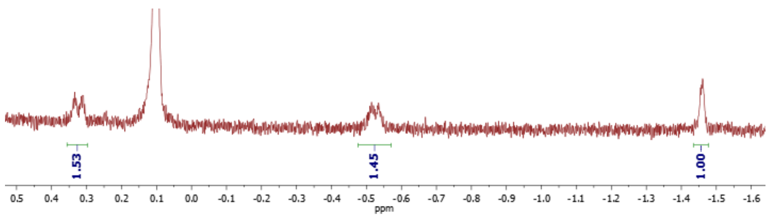

Fig. S61: Hydridic region of the ^{1}H NMR spectrum of **5** + 5 equiv. norbornene in d_8-THF at 298 K under 1 bar H_2 pressure.

Then, 20 equiv. of Nbe was added and the experiment was repeated. After full conversion, the same results were obtained with no observable catalyst degradation.

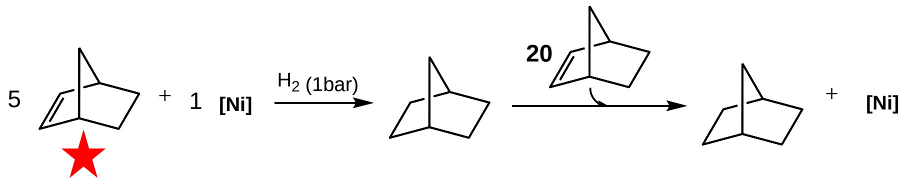

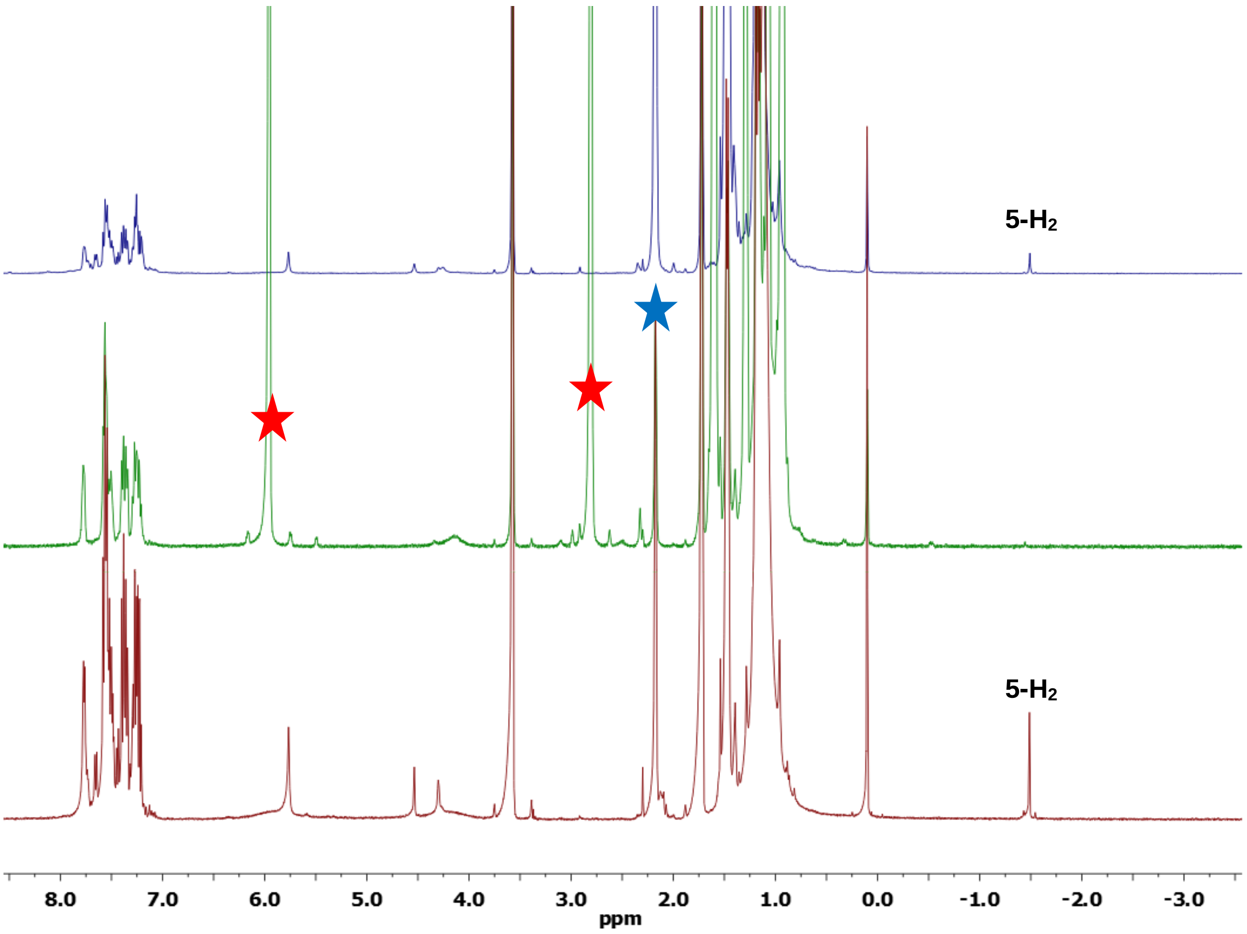

Fig. S62: ^{1}H NMR spectrum of **5** + 20 equiv. norbornene in d_8-THF at 298 K under 1 bar H_2 pressure.

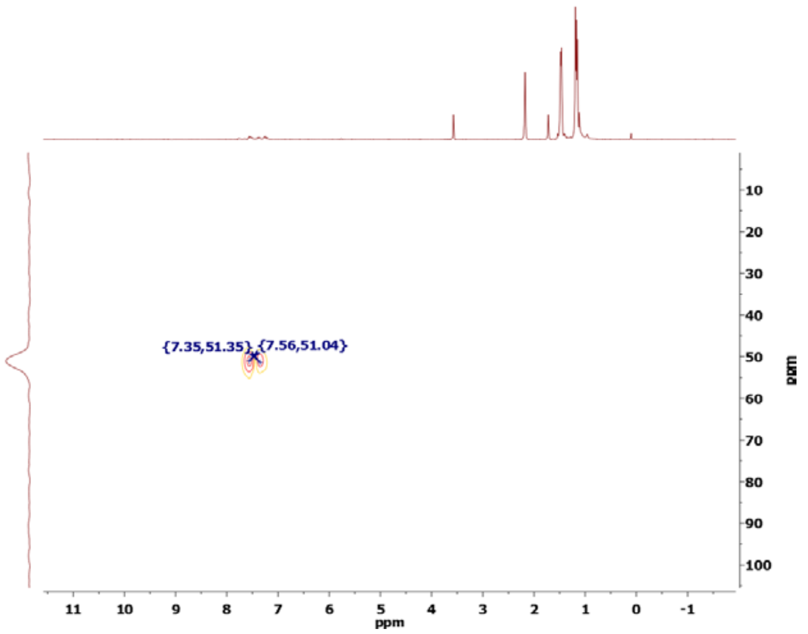

Fig. S63: ^{1}H, ^{29}Si HMQC NMR spectrum (optimized for $J_{Si,H}$ = 4 Hz) after hydrogenation of 25 equiv. of norbornene with catalyst **5** as only detectable specie.

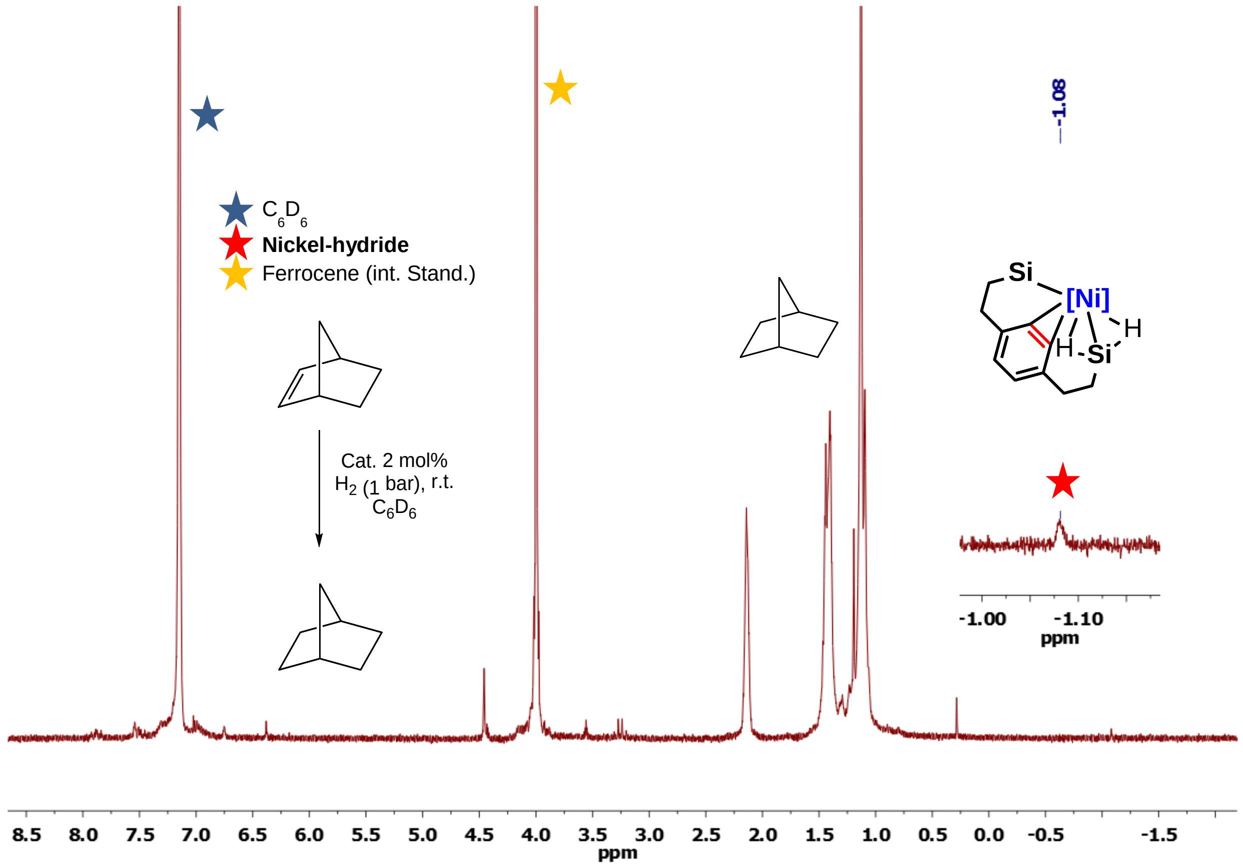

Fig. S64: ^{1}H NMR spectrum of norbornene hydrogenation using 2 mol% of catalyst **5** after full conversion showing no decomposition and the appearance of **5-H$_2$**.

Kinetic isotope effect:

Two NMR-samples were prepared, each containing 2 mol% of **5** (0.8 mg, 1.1 µmol) from a stock solution in C_6D_6 containing ferrocene as internal standard (0.022 mmol, 4 mg) and norbornene (0.054 mmol, 5 mg). ^{1}H-NMR spectra were obtained at regular intervals approx. 1 h and the NMR tubes were shaken after each measurement. The concentration of norbornene was plotted against time and the data points were fitted with a linear function (R^2 = 0.98/0.99).

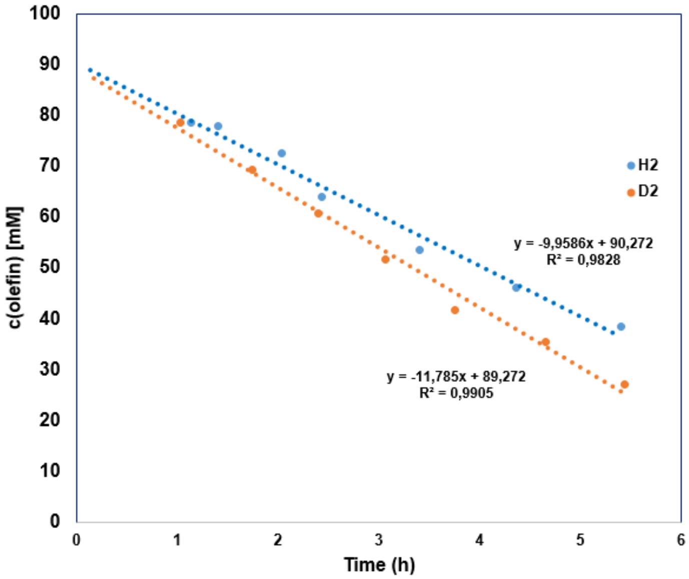

Fig. S65: Reaction profile of nbe reduction using H_2/D_2 for KIE determination.

The KIE was calculated using the following equation:

$$KIE = \frac{k_H}{k_D} = \frac{-9.96}{-11.78} = 0.85$$

TOF / TON measurement

A mixture of norbornene (**9f**, 1.07 mmol), ferrocene (0.43 mmol, internal standard) and **5** (1.07 µmol) was prepared inside a 3 mL vial and dissolved in 3 mL C_6D_6. The obtained red solution was transferred to a 25 mL Schlenk tube containing a stir bar. After three freeze-pump-thaw cycles, the solution was subjected to 1 bar H_2 at room temperature. The stir bar rotation was set at 1400 min^{-1}. The reaction progress was monitored by ^{1}H NMR spectroscopy by removal of 0.1 mL from the reaction mixture.

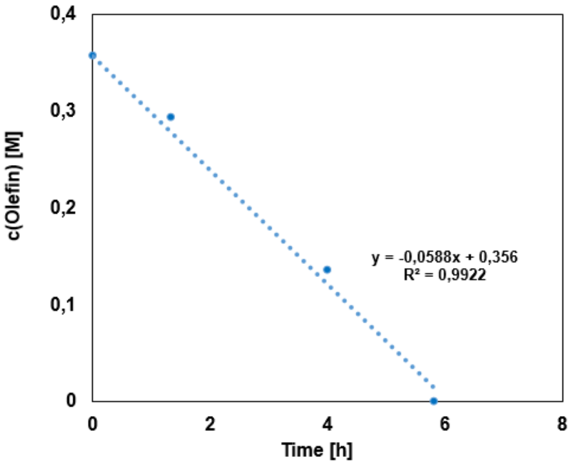

Fig. S66: Reaction profile of nbe hydrogenation using 0.1 mol% cat. **5** in C_6D_6 inside a Schlenk tube.

A full conversion was achieved after 5.8 h using 0.1 mol% of catalyst **5** resulting in an TON of TON = 1000 and TOF = 170 h^{-1}, respectively. The same experiment was conducted using 3.72 mmol norbornene in a 100 mL Schlenk tube resulting in a full conversion after 22 h (TON = 3700) with a comparable turn over frequency of TOF = 160 h^{-1}.

When 3,3-dimethyl-1-butene (**9a**) is employed as olefin (4.35 mmol, 250 mL Schlenk tube), a TON value of TON = 4350 was achieved within 14 h (conv. >99 %, TOF = 320 h^{-1}). For the TON_{MAX} determination, 1.2 mL (9.31 mmol) 3,3-dimethyl-1-butene (**9a**) in d_8-THF (1.0 mL) was exposed to 1 bar H_2. After 48 h, a TON value of TON_{MAX} = 9800 was achieved (conv. 98 %).

Determination of Reaction order in [olefine], [Ni]

Two NMR-samples were prepared, each containing 2 mol% of **5** (0.8 mg, 1.1 µmol) from a stock solution in C_6D_6 containing ferrocene as internal standard (0.022 mmol, 4 mg) and 5 / 2.5 mg of norbornene. Prior to H_2 addition, a ^{1}H NMR was recorded. After addition of H_2, ^{1}H-NMR spectra were obtained at intervals of approx. 1 h. The concentration of norbornene was plotted against time (8 hours) and the data points were fitted with a linear function ($R^2 > 0.98$).

c_0 (Nbe)	TON
104 mM	27
51 mM	25

For both concentrations of nbe, a very similar TON was observed within 8 hours. We conclude, that the reaction is 0. order in [Olefin]. The TOF value after >99% conversion was the same (3.2 h^{-1}). We conclude, that the reaction is 1. order in [**5**].

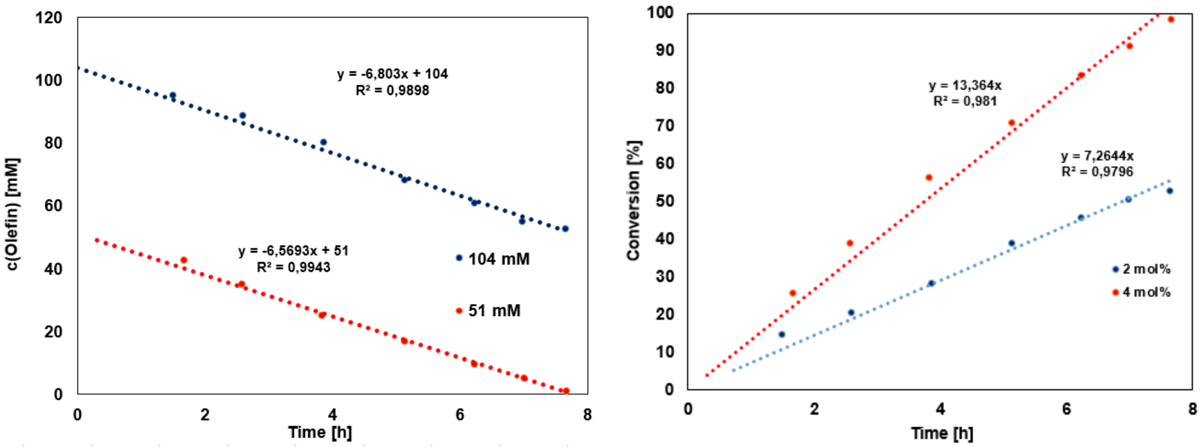

Fig. S67: Rate dependence on the olefin concentration c(olefin) which is 0. order and 1. Order in [**5**].

Determination of Reaction order in [H_2]

Three solutions were prepared, each containing 0.5 mol% of **5** (1.1 µmol) from a stock solution in C_6D_6 (1.5 mL) containing ferrocene as internal standard (0.088 mmol, 16 mg) and 20 mg of norbornene. The solutions were subjected to different H_2 pressures (1.0, 2.0, 3.0 bar) inside a Schlenk tube containing a stir bar after three freeze-pump-thaw cycles. During hydrogenation, the H_2-cylinder was directly attached to the Schlenk tube to ensure a constant H_2 pressure. After t = 10 min, 0.1 mL of the reaction mixture were removed and analyzed by ^{1}H NMR spectroscopy. The conversion of norbornene was plotted against the H_2-pressure and the data points were fitted with a linear function. A linear correlation was obtained ($R^2 = 0.98$). We conclude, that the reaction is 1. order in [H_2].

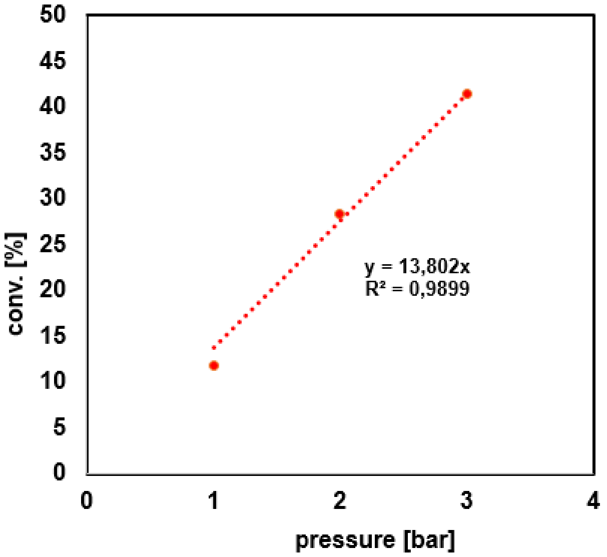

Fig. S68: Obtained alkane concentration at different H_2 pressure after t = 10 min.

Calculation of the experimental date law:

The overall reaction rate can be expressed by the following equation based on our experimental findings discussed above:

$$[Alkane]_t = -\,k[Ni][H_2]$$

The Henry`s constant used for the calculation of [H_2] in benzene is k_H = 341 Mpa.[21]

Pressure [Mpa]	X_i	Conc. H_2 [mol/L]
0.1	$2{,}931 \cdot 10^{-4}$	$3.42 \cdot 10^{-3}$
0.2	$5{,}86 \cdot 10^{-4}$	$6.84 \cdot 10^{-3}$
0.3	$8{,}79 \cdot 10^{-4}$	$10.26 \cdot 10^{-3}$

With [Ni] = 0.00018 mol/L and *k* = 0.7451 L/mol·min.

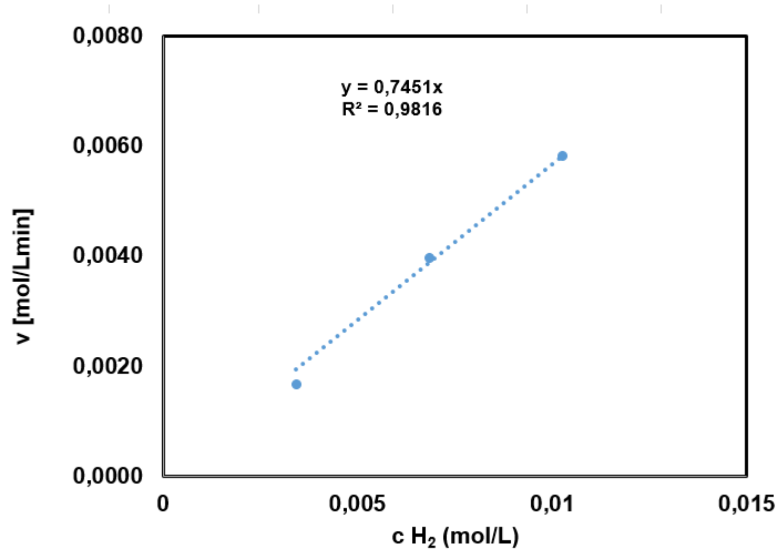

Fig. S69: Obtained data using different H_2 pressure after 10 min.

The overall experimental rate law is $v = k[\mathbf{5}][H_2]$ where k = 69 $M^{-1} \cdot sec^{-1}$.

Hammet-Plot analysis

Four solutions were prepared in a vial, each containing 0.5 mol% of **5** (1.1 µmol) from a stock solution in C_6D_6 (1.5 mL) containing ferrocene as internal standard (0.088 mmol, 16 mg) and *para*-substituted styrene derivatives (0.216 mmol). The solutions were transferred into a 10 mL Schlenk tube containing a stir bar. After three freeze-pump-thaw cycles, the solution was subjected to 1 bar H_2 at room temperature. The reaction progress was monitored by ^{1}H NMR spectroscopy by removal of 0.1 mL from the reaction mixture within the first 3 hours. Data points were fitted with a linear function ($R^2 = 0.99$).

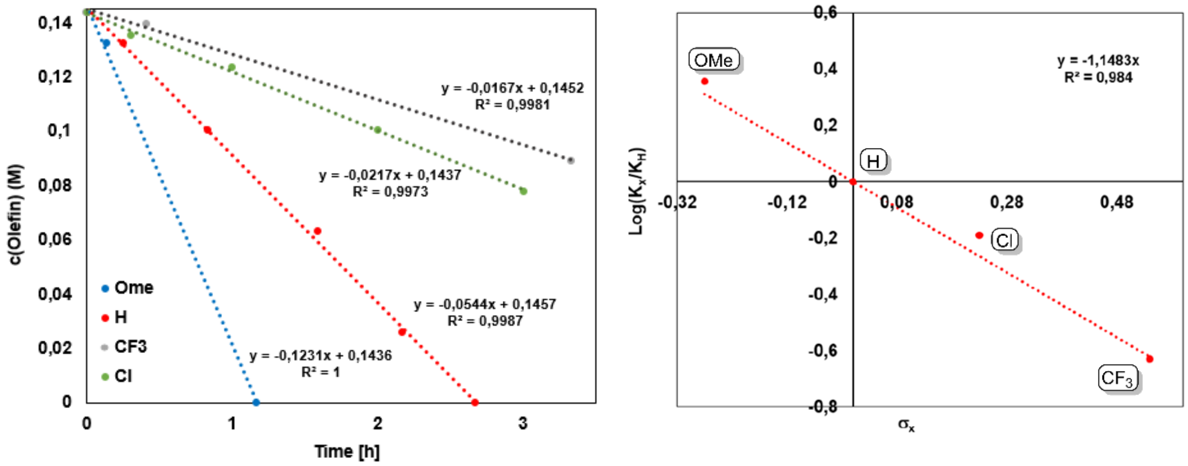

Fig. S70: Generated Hammet-Plot from conversion of different substituted *para*-styrene derivatives.

Solvent effect

Two vials were prepared, each containing 0.5 mol% of **5** (0.8 mg, 1.1 µmol) and ferrocene as internal standard (0.088 mmol, 16 mg). After dissolving the mixture in C_6D_6 / d_8-THF (1.5 mL), 4-methoxystyrene (0.216 mmol) was added and the solutions were transferred into a 10 mL Schlenk tube containing a stir bar. After three freeze-pump-thaw cycles, the solution was subjected to 1 bar H_2 at room temperature. The reaction progress was monitored by ^{1}H NMR spectroscopy by removal of 0.1 mL from the reaction mixture. Full conversion was indicated by a color change from orange to red as the catalyst is regenerated after full conversion. ^{1}H NMR analysis revealed a full hydrogenation of the olefin. [olefin] was plotted against time and the data points were fitted with a linear function (R^2 = 0.99).

Solv.	Time [min], conv. > 99%	TON	TOF [h^{-1}]
C_6D_6	62	200	200
d_8-THF	43	200	334

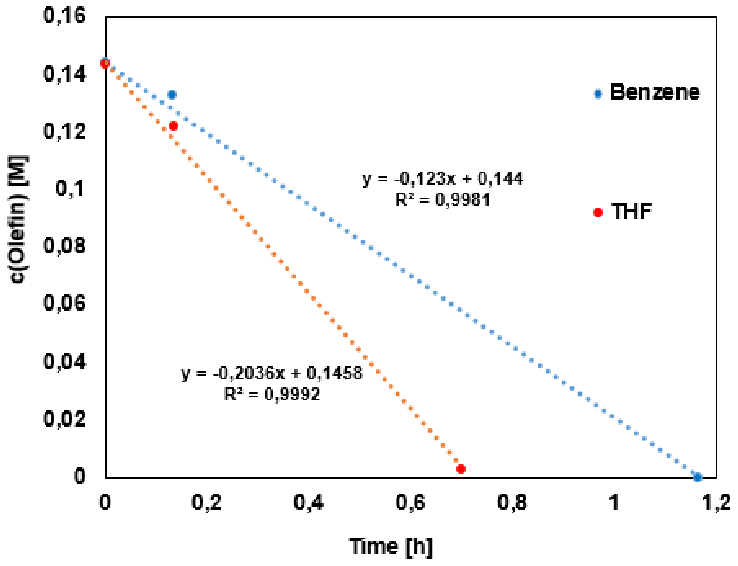

Fig. S71: Determination of solvent effect using benzene and THF on 4-methoxystyrene hydrogenation.

Cycling Experiment on 3,3-dimethyl-1-butene hydrogenation

A stock solution was used containing ferrocene (0.022 mmol/0.45 mL) and catalyst **5** (1.1 µmol/0.45 mL, 2 mol%). From the stock solution, 0.45 mL were taken and transferred into a sealed J. Young NMR tube and 3,3-dimethyl-1-butene (6.9 µL, 0.054 mmol) was added. Prior to H_2 addition, a ^{1}H NMR spectrum was recorded. After three freeze-pump-thaw cycles, the solution was subjected to 1 bar H_2 at room temperature. The reaction progress was further monitored by ^{1}H NMR analysis. After 3 h, full conversion was achieved (Cycle 1). The experiment was repeated upon addition of another portion of 3,3-dimethyl-1-butene (6.9 µL, 0.054 mmol) (Cycle 2) resulting in a quantitative hydrogenation after 3 h. After the second portion, no catalyst degradation was observed leading to the observation of **5-H$_2$**.

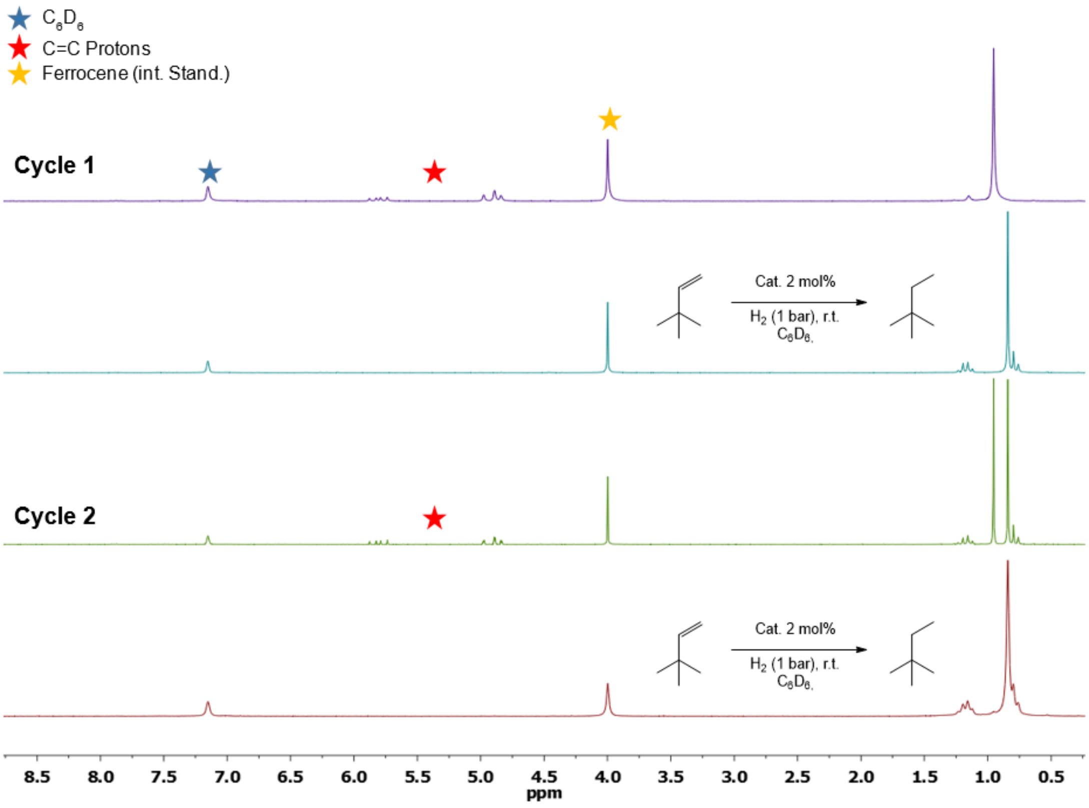

Fig. S72: ^{1}H NMR spectrum of the reaction mixture prior H_2 addition containing 3,3-dimethyl-1-butene, ferrocene and 2 mol% catalyst **5** (top). After complete hydrogenation (t = 3 h), 50 equiv. of olefin was added and hydrogenated within 3 hours.

11) Crystallographic Data

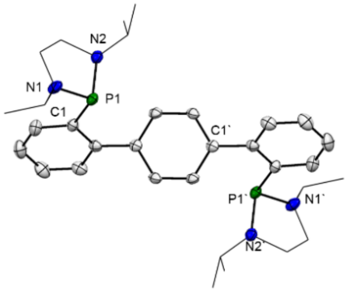

Empirical formula	$C_{38} H_{48} N_4 P_2$	
Formula weight	574.70	
Temperature	150(2) K	
Wavelength	1.54184 Å	
Crystal system	triclinic	
Space group	P-1	
Unit cell dimensions	a = 7.5552(5) Å	α = 76.651(5)°.
	b = 9.5169(6) Å	β = 84.110(5)°.
	c = 11.8279(6) Å	γ = 72.543(6)°.
Volume	788.85(9) $Å^3$	
Z	1	
Density (calculated)	1.210 Mg/m^3	
Absorption coefficient	1.463 mm^{-1}	
F(000)	310	
Crystal size	0.640 x 0.220 x 0.120 mm^3	
Theta range for data collection	3.843 to 67.486°.	
Index ranges	-8<=h<=8, -10<=k<=11, -14<=l<=14	
Reflections collected	4857	
Independent reflections	2831 [R(int) = 0.0156]	
Completeness to theta = 67.42°	99.7 %	
Absorption correction	Semi-empirical from equivalents	
Max. and min. transmission	1.00000 and 0.45220	
Refinement method	Full-matrix least-squares on F^2	
Data / restraints / parameters	2831 / 0 / 185	
Goodness-of-fit on F^2	1.050	
Final R indices [I>2sigma(I)]	R_1 = 0.0377, wR_2 = 0.1020	
R indices (all data)	R_1 = 0.0396, wR_2 = 0.1039	
Largest diff. peak and hole	0.339 and -0.364 e.$Å^{-3}$	

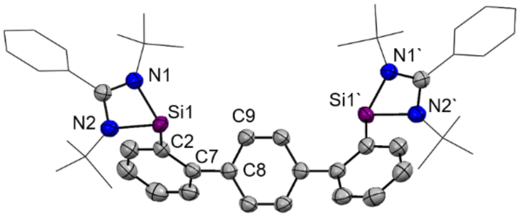

Empirical formula	$C_{48} H_{58} N_4 Si_2$	
Formula weight	747.16	
Temperature	150(2) K	
Wavelength	0.71073 Å	
Crystal system	monoclinic	
Space group	C2/c	
Unit cell dimensions	a = 25.0720(15) Å	α = 90°.
	b = 9.1667(4) Å	β = 99.889(6)°.
	c = 19.7603(11) Å	γ = 90°.
Volume	4716.67(13) Å^3	
Z	4	
Density (calculated)	1.109 Mg/m^3	
Absorption coefficient	0.115 mm^{-1}	
F(000)	1608	
Crystal size	0.210 x 0.060 x 0.030 mm^3	
Theta range for data collection	2.092 to 26.230°	
Index ranges	-31<=h<=30, -11<=k<=11, -23<=l<=24	
Reflections collected	16755	
Independent reflections	4440 [R(int) = 0.0793	
Completeness to theta = 67.42°	99.9 %	
Absorption correction	Semi-empirical from equivalents	
Max. and min. transmission	1.00000 and 0.45220	
Refinement method	Full-matrix least-squares on F^2	
Data / restraints / parameters	4440 / 66 / 302	
Goodness-of-fit on F^2	1.023	
Final R indices [I>2sigma(I)]	R_1 = 0.0632, wR_2 = 0.1412	
R indices (all data)	R_1 = 0.1185, wR_2 = 0.1746	
Largest diff. peak and hole	0.337 and -0.198 e.Å^{-3}	

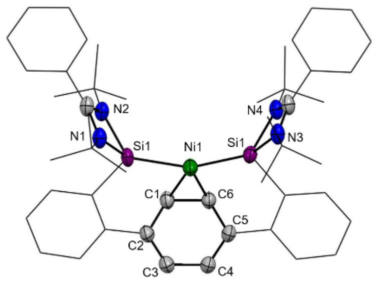

Empirical formula	C_{55} H_{66} N_4 Ni Si_2	
Formula weight	898.01	
Temperature	150.00(10) K	
Wavelength	1.54184 Å	
Crystal system	monoclinic	
Space group	P21/m	
Unit cell dimensions	a = 9.1662(5) Å	α = 90°.
	b = 26.4418(13) Å	β = 90.567(5)°.
	c = 9.8935(6) Å	γ = 90°.
Volume	2397.8(2) $Å^3$	
Z	2	
Density (calculated)	1.244 Mg/m^3	
Absorption coefficient	1.366 mm^{-1}	
F(000)	960	
Crystal size	0.32 x 0.08 x 0.08 mm^3	
Theta range for data collection	4.47 to 67.49°.	
Index ranges	--10<=h<=10, -29<=k<=31, -7<=l<=11	
Reflections collected	7952	
Independent reflections	4355 [R(int) = 0.0390]	
Completeness to theta = 67.42°	98.8 %	
Absorption correction	Semi-empirical from equivalents	
Max. and min. transmission	0.8986 and 0.6721	
Refinement method	Full-matrix least-squares on F^2	
Data / restraints / parameters	4355 / 549 / 529	
Goodness-of-fit on F^2	1.194	
Final R indices [I>2sigma(I)]	R_1 = 0.0982, wR_2 = 0.2212	
R indices (all data)	R_1 = 0.1221, wR_2 = 0.2417	
Largest diff. peak and hole	3.847 and -0.989 e.$Å^{-3}$	

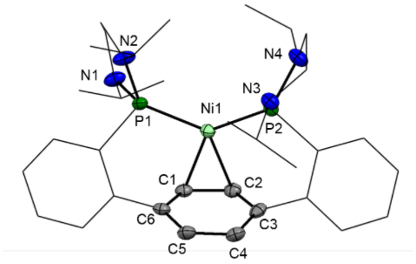

Empirical formula	$C_{34} H_{48} N_4 Ni P_2$	
Formula weight	633.41	
Temperature	150(2) K	
Wavelength	1.54184 Å	
Crystal system	triclinic	
Space group	P-1	
Unit cell dimensions	a = 10.1955(3) Å	α = 76.412(3)°.
	b = 10.3773(3) Å	β = 80.446(3)°.
	c = 16.0898(6) Å	γ = 88.451(3)°.
Volume	1631.60(9) $Å^3$	
Z	2	
Density (calculated)	1.289 Mg/m^3	
Absorption coefficient	1.998 mm^{-1}	
F(000)	676	
Crystal size	0.340 x 0.320 x 0.190 mm^3	
Theta range for data collection	2.864 to 67.499°.	
Index ranges	-12<=h<=12, -12<=k<=12, -	
19<=l<=19Reflections collected	10913	
Independent reflections	5876 [R(int) = 0.0211]	
Completeness to theta = 67.42°	99.8 %	
Absorption correction	Semi-empirical from equivalents	
Max. and min. transmission	1.00000 and 0.45220	
Refinement method	Full-matrix least-squares on F^2	
Data / restraints / parameters	5876 / 0 / 390	
Goodness-of-fit on F^2	1.050	
Final R indices [I>2sigma(I)]	R_1 = 0.0326, wR_2 = 0.0856	
R indices (all data)	R_1 = 0.0348, wR_2 = 0.0870	
Largest diff. peak and hole	0.311 and -0.417 e.$Å^{-3}$	

12) References:

[S1] A. Velian, S. Lin, A. J. M. Miller, M. W. Day, T. Agapie, *J. Am. Chem. Soc.* **2010**, 132, 18, 6296-6297.

[S2] J. S. Bair, Y. Schramm, A. G. Sergeev, E. Clot, O. Eisenstein, J. F. Hartwig, *J. Am. Chem. Soc.* **2014**, 136, 13098-13101.
